# Adaptive Coordination Behavior of Bisphosphanylphosphanido‐Ligands Toward Group 2, 11 and 12 Metal Ions

**DOI:** 10.1002/chem.202500406

**Published:** 2025-03-11

**Authors:** Dennis Langgut, Clemens Bruhn, Rudolf Pietschnig

**Affiliations:** ^1^ Institute for Chemistry University of Kassel Heinrich-Plett-Straße 40 34132 Kassel Germany

**Keywords:** Phosphanes, Alkaline earth metals, Coinage metals, NMR spectroscopy, X-ray diffraction

## Abstract

A series of alkaline earth metal complexes (Mg−Ba) with the anionic ferrocenylene‐bridged bisphosphanylphosphanide ligand [Fe(C_5_H_4_PC_4_H_9_)_2_P]^−^ has been prepared by metalation of the corresponding bisphosphanylhydrophosphane (Fe(C_5_H_4_PC_4_H_9_)_2_PH). The resulting complexes have been characterized by multinuclear NMR spectroscopy and SC‐XRD. Furthermore, the closely related zinc phosphanide, and rare, mononuclear coinage metal phosphanides were synthesized and investigated for comparison. The range of coordination modes observed for the phosphanide ligand included mono‐, bi‐, and tridentate modi and their respective combinations, whereas no *μ*_2_‐bridging of the phosphanide center has been observed. The value of the ^1^ *J*_PP_ coupling constant was found to be a good probe to track the coordination motif consistent with the situation in solid state.

## Graphical abstract

Combination of 2, 11 and 12 metal ions with a bisphosphanylphosphanide counter anion affords versatile and adaptive coordination motifs, for which NMR parameters (^1^ *J*_PP_) serve as an easy and reliable diagnostic tool consistent with the situation in solid state as corroborated by single crystal X‐ray crystallography.

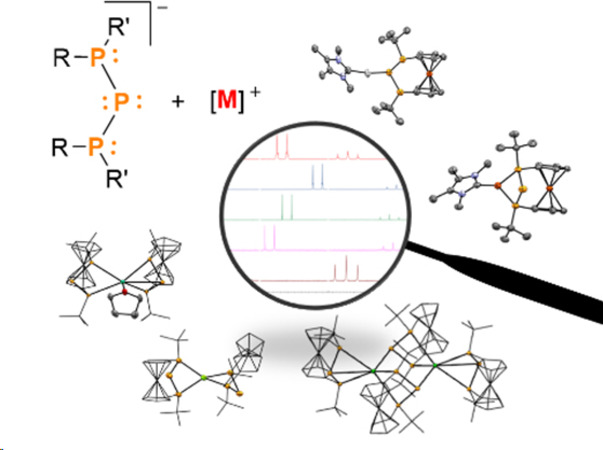

## Introduction

**Figure 1:**
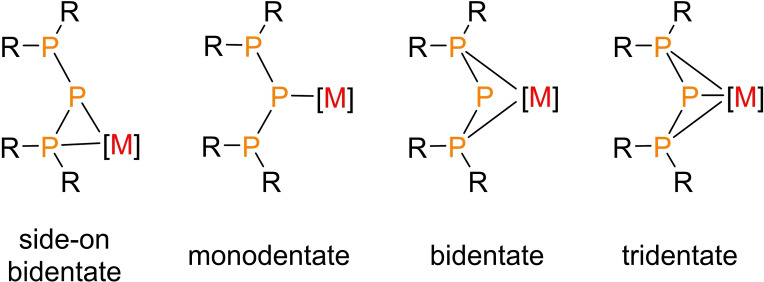
Schematic representation of possible coordination modes featuring metal [M] complexes with (R_2_P)_2_ P^–^‐ligands. Formal charges and lone pairs are not shown.

Catenated oligophosphanes are known for their structural diversity and have been the subject of ongoing research for decades.[^1^, ^2^, ^3^] (Oligo)phosphanides are their anionic counterparts which can act as bridging ligands owing to the presence of two geminal electron pairs on at least one phosphorus atom, giving rise to a broad variety of coordination patterns.[^4^, ^5^, ^6^, ^7^] In addition, phosphanido ligands can adopt either a planar or a pyramidal geometry depending on whether π‐bonding is possible or not.^[8]^ If the substituents adjacent to the formally charged phosphanide atom are Lewis‐basic as well, such as in bisphosphanylphosphanido [(R_2_P)_2_P^–^]‐ligands, many more coordination modes are possible, often depending on the nature of the metal center, the basicity of the participating phosphorus atoms and their substitution pattern. Several recent publications revolving around (R_2_P)_2_P^–^ ligands show this coordinative flexibility, with the ligands often being coordinated side‐on,[^9^, ^10^, ^11^, ^12^, ^13^] monodentate,[^14^, ^15^, ^16^, ^17^, ^18^, ^19^] bidentate,[^17^, ^18^, ^19^, ^20^, ^21^, ^22^] or tridentate[^18^, ^23^] (Figure 1) and sometimes entailing P−P bond cleavage.[^11^, ^18^]

Our group developed a stereochemically constrained triphosphanyl scaffold **1‐H** in which the geometrically flexible ferrocenylene‐bridge limits the number of diastereomers originating from the presence of P‐stereogenic centers.^[24]^ Upon deprotonation of the central P‐atom by various strong bases, a series of alkali metal phosphanides has been prepared. The latter all showed a bidentate coordination of the alkali metal ion by the terminal, formally uncharged P‐atoms, surprisingly, without direct interaction of the formal anionic atom with its counter cation.^[20]^ This phenomenon has been observed for some transition metals as well.[^17^, ^18^, ^19^, ^21^, ^22^] Inspired by these findings, we set out to explore how coordination of such phosphanides would change towards a series of alkaline earth metal ions of increasing size. Similarly, we prepared diamagnetic coinage metal as well as zinc complexes for structural investigation supported by NMR spectroscopy, to gain further insights into the multifaceted chemistry of phosphanides.

## Results and Discussion

### Alkaline Earth and Zinc Phosphanides

**Scheme 1:**
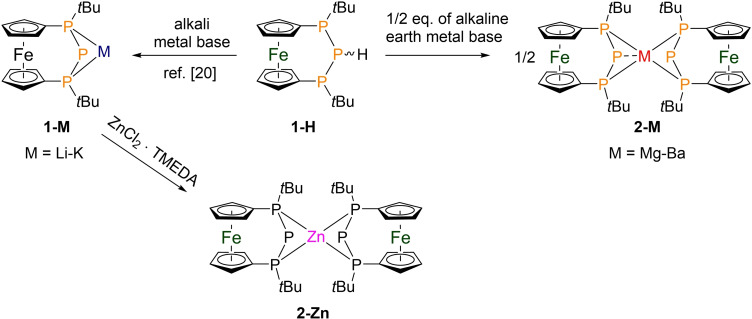
Synthetic access to **2‐M** via metalation of a diastereomeric mixture of **1‐H** with alkaline earth metal bases (M=Mg: Mg(CH_2_SiMe_3_)_2_; M=Ca−Ba: M(N(SiMe_3_)_2_)_2_). Similar deprotonation utilizing alkali metal bases (*n*‐BuLi; NaH, KO*t*‐Bu) has been reported before.^[20]^ The corresponding zinc phosphanide **2‐Zn** was prepared by salt‐metathesis reaction starting from **1‐Li**.

To further explore the coordination preferences of bisphosphanylphosphanide, we have chosen alkaline earth metal dications (Ae^2+^), particularly, because they are redox innocent and connected via the diagonal relationship with the alkali metals for which we had explored the coordination motifs in a previous study.^[20]^ Moreover, for the heavier Ae elements, (n–1) d‐orbitals may be involved in chemical bonding starting from Ca. This has been shown to influence covalency in chemical bonds, resulting in unusual geometries and increased solubility in organic solvents.[^25^, ^26^, ^27^, ^28^, ^29^] With magnesium featuring a diagonal relationship with the lightest alkali metal lithium, we limited our investigations to the common alkaline earth metals Mg, Ca, Sr and Ba, excluding beryllium owing to potential toxicity issues.^[30]^ As a general synthetic strategy, the straightforward metalation of secondary phosphanes with strong bases is well‐established[^31^, ^32^, ^33^] and also proved successful in our case (Scheme 1). According to ^31^ P NMR spectroscopy, nearly quantitative yields were achieved, with volatile compounds forming as by‐products. After removing the latter in vacuum, followed by a washing step with *n*‐pentane, the corresponding alkaline earth phosphanides were isolated in excellent purity.

**Figure 2:**
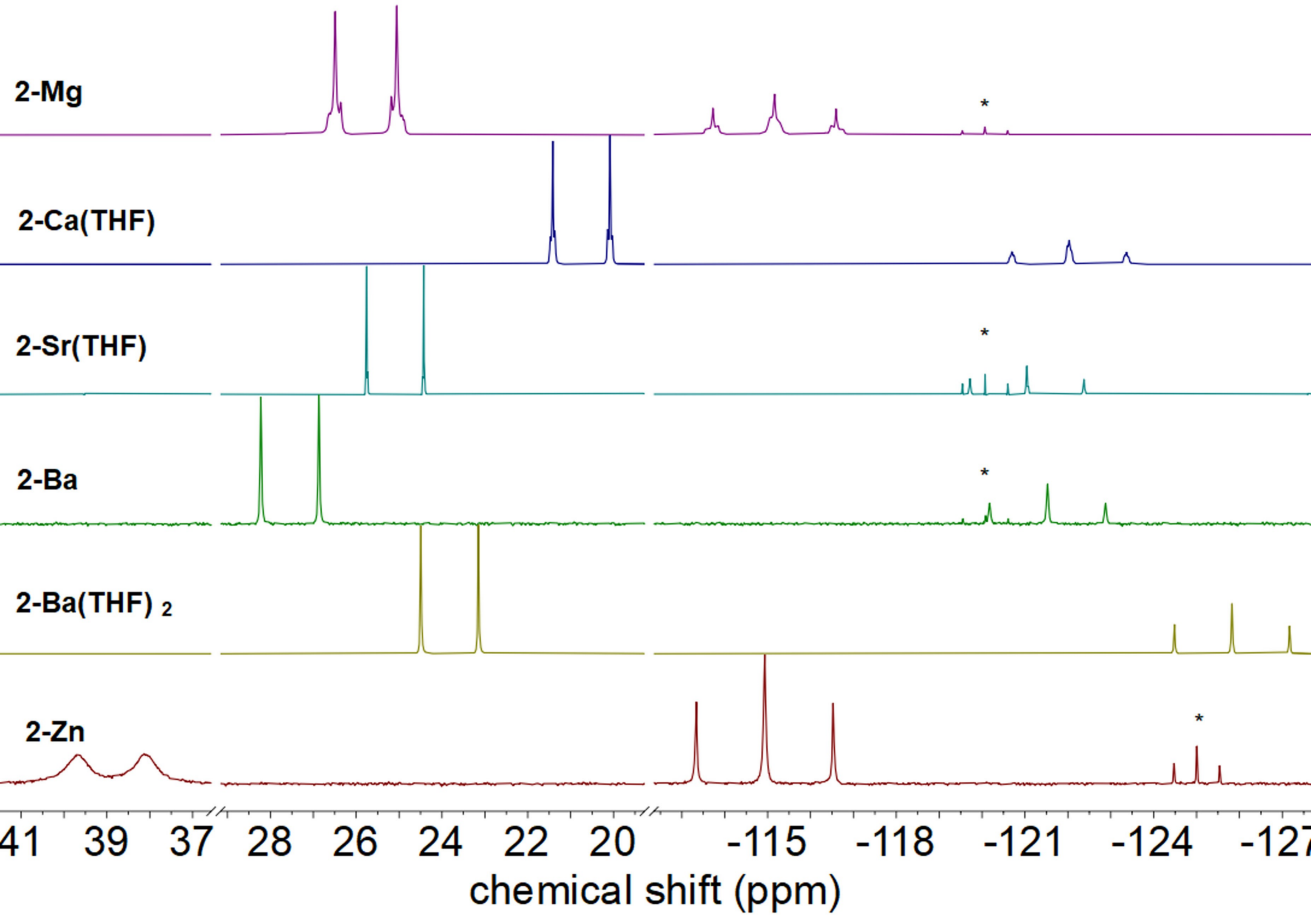
Sections from the ^31^P{^1^H} NMR spectra of compounds **2‐Mg**‐**2‐Ba(THF)_2_** (solvent: toluene‐*d*_8_) and **2‐Zn** (solvent: C_6_D_6_). The asterisk (*) marks the resonance of the starting material **1‐H**.

As established in previous work, the ferrocene backbone favours the *meso*‐isomer in which the stereogenic outer phosphorus atoms adopt opposite absolute configuration. This feature is retained in the corresponding phosphanide derived from **1‐H**, which increases the molecular symmetry resulting in relatively simple coupling patterns in the ^31^P NMR spectra. Consequently, both chemically equivalent terminal P‐atoms give rise to a doublet while the central, formal phosphanide P‐atom gives rise to a triplet. This was observed for the corresponding symmetric alkali metal phosphanides,[^20^, ^34^] and also holds true for the alkaline earth metal phosphanides reported herein. However, a closer look at the ^31^P NMR spectra of **2‐Mg**, **2‐Ca(THF)**, and **2‐Sr(THF)** reveals signals of higher order, likely due to ^3^ *J*_PP_‐coupling between P‐atoms over the metal ions (Figure 2). When comparing NMR‐spectroscopic data, it needs to be considered that the preparation of **2‐Ca(THF)** was done by deprotonation of **1‐H** using Ca(N(SiMe_3_)_2_)_2_ ⋅ 2 THF, resulting in the coordination of one molecule of THF to the central metal atom both in solution and in solid state. Moreover, the metalation using Sr(N(SiMe_3_)_2_)_2_ suffered from poor reproducibility in non‐polar solvents and required THF as reaction medium, also leading to the coordination of one THF molecule to the central Sr^2+^ ion of **2‐Sr(THF)**. The signals of the coordinated THF can be identified in the respective ^1^H NMR spectra (see Figure S7 and Figure S11). Furthermore, the solvent‐free barium phosphanide (**2‐Ba**) could be characterized, in addition to its solvate incorporating two THF molecules per barium (**2‐Ba(THF)_2_**). Therefore, these compounds are particularly suitable for direct comparison.

The value of the ^3^ *J*_PP_ coupling constant decreases continuously from **2‐Mg** to **2‐Ba**, and as a result, no such higher order resonances can be resolved for **2‐Ba** anymore. This well resolved coupling pattern is remarkable, since in other cases frequently broad signals have been observed.[^31^, ^32^, ^35^, ^36^, ^37^, ^38^, ^39^, ^40^] The latter observation has been attributed to rapid ligand exchange or dynamic monomer‐dimer equilibria, in which the phosphanide centers act as bridging ligand, depending on solvent and temperature. The monomeric nature of these complexes in solution is further corroborated by ^1^H‐DOSY NMR measurements (see Figure S17) in non‐polar solvents. Remarkably, the chemical shift of the formally negatively charged central P‐atom hardly varies, whereas the coordinating terminal P‐atoms show a slight downfield shift with increasing radius of the metal atom. This observation is consistent with previous data and can be rationalized by an increased electron density at the donor atoms and an enhancement of the ionicity of the M−P bond with the increasing electropositive nature (and ionic radius) of the group 2 metal.[^32^, ^35^, ^38^, ^41^] A tendency to larger differences in chemical shifts of the terminal and the central P‐atoms |Δ(δP_central_‐δP_terminal_)| for larger M^2+^ ions can be found, as well as generally larger differences when compared to the corresponding alkali metal phosphanides.^[20]^ Owing to similar ionic radii and electronegativities,^[42]^ zinc is known to have some similarities with the lighter Ae metals, notably magnesium. Therefore, we explored the coordination behaviour of Zn^2+^ to the bisphosphanylphosphanide‐ligand under discussion as well. To this end, **1‐Li** was reacted with ZnCl_2_ ⋅ TMEDA in a salt‐metathesis reaction. The ^31^P NMR spectra of compound **2‐Zn** in C_6_D_6_ confirms the trends found for the alkaline earth metal phosphanides: While the resonance of the formal phosphanide atom is barely influenced, the signals of the terminal P‐atoms are shifted towards lower field and the value of the ^1^ *J*_PP_ coupling constant even exceeds that of **2‐Mg** (see Table 1). By contrast to the previously discussed systems, the doublet is broadened, which can be attributed to a fast reversible dechelatation of one phosphine ligand in solution that has been proposed for similar systems before.[^43^, ^44^] The ^31^P NMR data are summarized in Table 1. Notably, **2‐Mg** stands out of the other alkaline earth phosphanides featuring values very close to **2‐Zn**, thus underlining the above‐mentioned similarity between the two types of ions.

**Table 1:** Selected NMR data of the alkaline earth metal (**2‐Mg**‐**2‐Ba**) bisphosphanylphosphanides along with their protonated congener **1‐H**, the lithiated **1‐Li** and the corresponding zinc analogue **2‐Zn**.

	**1‐H** ^[a][24]^	**2‐Mg** ^[b]^	**2‐Ca(THF)** ^[b,e]^	**2‐Sr(THF)** ^[b,e]^	**2‐Ba** ^[b]^	**2‐Ba(THF)_2_** ^[b,d]^	**2‐Zn** ^[c]^	**1‐Li** ^[c][20]^
δ^31^P_central_ [ppm]	−74.3, −125.1	−115.2	−122.0	−121.0	−121.5	−125.8	−114.9	−116.4
δ^31^P_terminal_ [ppm]	−9.8, −6.5	25.8	20.8	25.1	27.5	23.8	38.9	14.9
|^1^ *J*_PP_| [Hz]	294, 107	290	270	268	274	272	321	271
|Δ(δP_central_‐δP_terminal_)| [ppm]	64.5, 118.6	141.0	142.8	146.1	149.0	149.6	153.8	131.3
δ^13^C(*ipso*‐Cp) [ppm]	74.8, 74.8	85.8	87.0	86.4	85.4	85.7	85.6	91.5

[a] The values for both *cis‐* and *trans*‐isomers are given. Solvent: C_6_D_6_. [b] Solvent: toluene‐*d*_8_. [c] Solvent: C_6_D_6_. [d] The metal ion is additionally coordinated by two THF molecules. [e] The metal ion is additionally coordinated by one THF molecule.

Upon metalation, the resonance of the *ipso‐*C‐atoms of the cyclopentadienyl‐rings are notably shifted to lower field (see Table 1), in line with previous observations.[^32^, ^35^, ^38^, ^41^] However, a simple correlation with the radii of the metal ions cannot be found. Additionally, the value of the ^1^ *J*_PC_ coupling constant, which has been shown to be associated with the chemical shift of the P‐atoms,^[41]^ is difficult to determine, as the corresponding carbon resonance features a complex multiplet pattern in the ^13^C NMR spectrum.

The influence of additional solvent coordination can be evaluated when comparing **2‐Ba** and **2‐Ba(THF)_2_**. While no significant differences can be observed in the ^1^H and ^13^C NMR spectra, a pronounced high‐field shift of the ^31^P NMR resonances is noticeable for **2‐Ba(THF)_2_** with the shift difference |Δ(δP_central_‐δP_terminal_)| remaining nearly constant (see Table 1). The shielding effect can be rationalized by a reduced *Lewis*‐acidity of the metal ion upon coordination of further donor ligands like THF.

**Figure 3:**
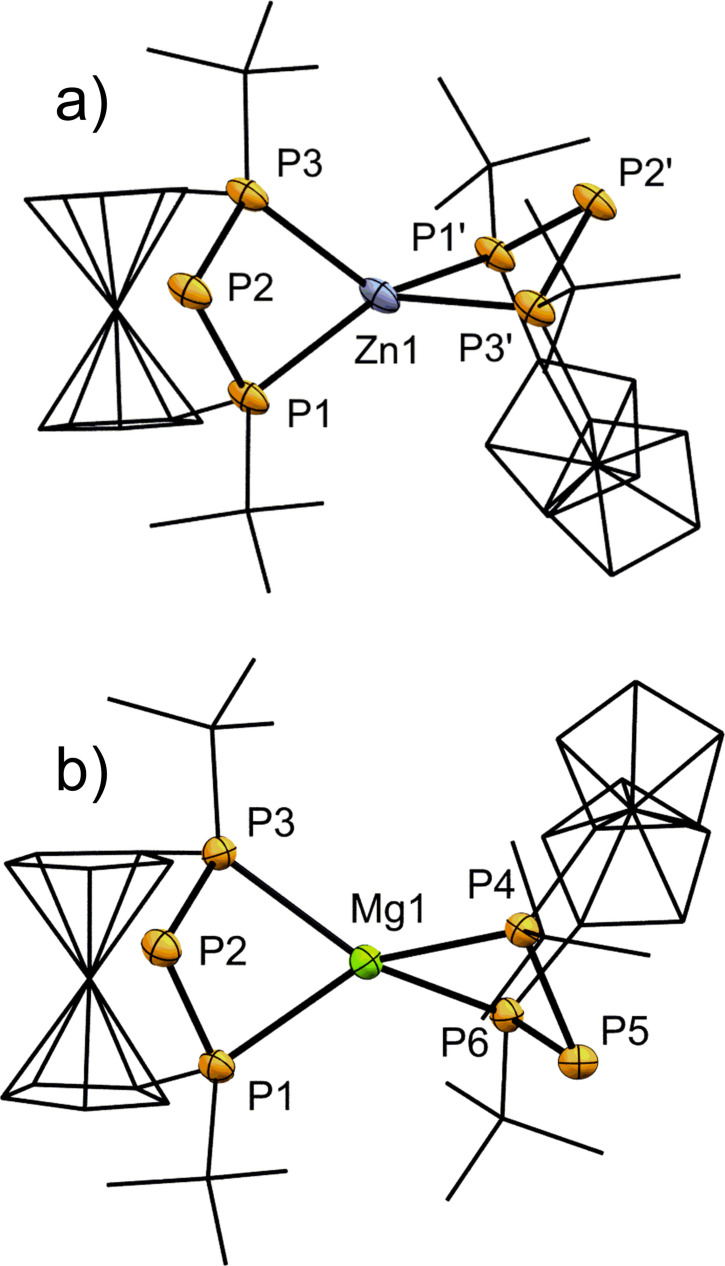
ORTEP‐depictions of a) **2‐Zn** and b) **2‐Mg**. Ferrocenylene‐units and *t*Bu‐groups are shown in wireframe‐style. Hydrogen atoms are omitted for clarity. The thermal ellipsoids are represented at a 30 % probability level. a) One co‐crystallising lattice benzene molecule is omitted. Selected bond lengths [Å] and angles [°]: P1−Zn1 2.4490(8), P2−Zn1 3.3372(8), P3−Zn1 2.4174(10), P1−P2 2.1510(13), P2−P3 2.1556(12), P1−P2−P3 83.93(5). b) Half a co‐crystallising lattice cyclopentane molecule is omitted. Selected bond lengths [Å] and angles [°]: P1−Mg1 2.5341(12), P2−Mg1 3.2050(13), P3−Mg1 2.5283(13), P4−Mg1 2.5379(13), P5−Mg1 3.2597(13), P6−Mg1 2.5322(13), P1−P2 2.1608(11), P2−P3 2.1558(11), P1−P2−P3 86.92(4), P4−P5−P6 85.90(4).

All the above‐mentioned phosphanides have been investigated in solid state by single crystal X‐ray diffraction. Selected bond lengths and angles are listed in Table 2. For **2‐Mg** and **2‐Zn** exclusive coordination at the terminal phosphorus atoms of the phosphanide ion with distorted pseudotetrahedral coordination of the metal ion is observed (Figure 3). The P−Zn bond lengths in **2‐Zn** range between 2.4174(10) and 2.4490(8) Å for the terminal phosphorus atoms which is comparable to similar systems.[^43^, ^45^, ^46^, ^47^] By contrast, the central phosphorus atom shows a P2–Zn1 distance (3.3372(8) Å) which is longer than a chemical bond yet shorter than the sum of the respective van der Waals‐radii (∑_vdW_=429 pm).^[48]^ The P−P bond lengths of **2‐Zn** are shortened compared to its protonated congener **1‐H** (2.203(1)–2.206(1) Å) and the P−P−P angles more acute (91.68° for **1‐H**).^[24]^ This also holds true for the alkaline earth metal phosphanides described herein, however, to a lesser degree, and has been observed for similar systems as well.[^13^, ^14^, ^15^] In a related P−N−P system, Kemp *et al*. observed a similar contraction which they attributed to partial delocalization, with the anionic charge being delocalized reminiscent of an allylic system.^[45]^ A reasonable alternative explanation involves an interaction of the phosphanide atom's lone pairs with unoccupied σ*‐orbitals of the adjacent phosphorus atoms (negative hyperconjugation).

**Table 2:** Selected distances [Å] and bond angles [°] of **2‐Mg**, **2‐Mg(THF)**, **2‐Ca(THF)**, **2‐Sr**, **2‐Ba**, and **2‐Zn**. As both P1−P2 and P2−P3 and P4−P5 and P5−P6 are usually of same length, latter values are omitted, respectively.

	**2‐Mg**	**2‐Mg(THF)**	**2‐Ca(THF)** ^[a]^	**2‐Sr**	**2‐Ba**	**2‐Zn** ^[b]^
P1−M1	2.5341(12)	2.686(2)	2.8752–2.9093(11)	3.0171(15)	3.4467(10)	2.4490(8)
P2−M1	3.2050(13)	3.615(2)	3.8552–3.9127(11)	3.3281(15)	3.3073(10)^[c]^	3.3372(8)
P3−M1	2.5283(13)	2.724(2)	2.9566–2.9644(11)	3.0385(15)	3.3105(10)	2.4174(10)
P4−M1	2.5379(13)	2.707(2)	2.9030–2.9233(11)	3.1789(15) ^[c]^	3.1327(10)	
P5−M1	3.2597(13)	3.579(2)	3.0632–3.0932(11)	3.1753(15)	3.3992(10)	
P6−M1	2.5322(13)	2.677(2)	2.8344–2.8364(10)	3.3070(15) ^[c]^	3.1720(10)	
P1−P2	2.1608(11)	2.1563(19)	2.1673–2.1704(13)	2.180(2)	2.1880(14)	2.1510(13)
P4−P5	2.1684(11)	2.169(2)	2.180–2.181(2)	2.193(2)	2.1739(14)	/
P1−P2−P3	86.92(4)	85.07(7)	86.45–87.22(4)	89.77(7)	89.66(5)	83.93(5)
P4−P5−P6	85.90(4)	84.82(7)	87.23–89.13(4)	90.20(7)	89.93(5)	/

[a] Two molecules with similar bonding parameters were found in the asymmetric unit. Therefore, a range is given for bond lengths. One THF molecule is bound to the metal atom. [b] The Zn1‐atom is an inversion center hence one half of the molecule is symmetry‐generated. c) The noted distance corresponds to P#’‐M1.

**Figure 4:**
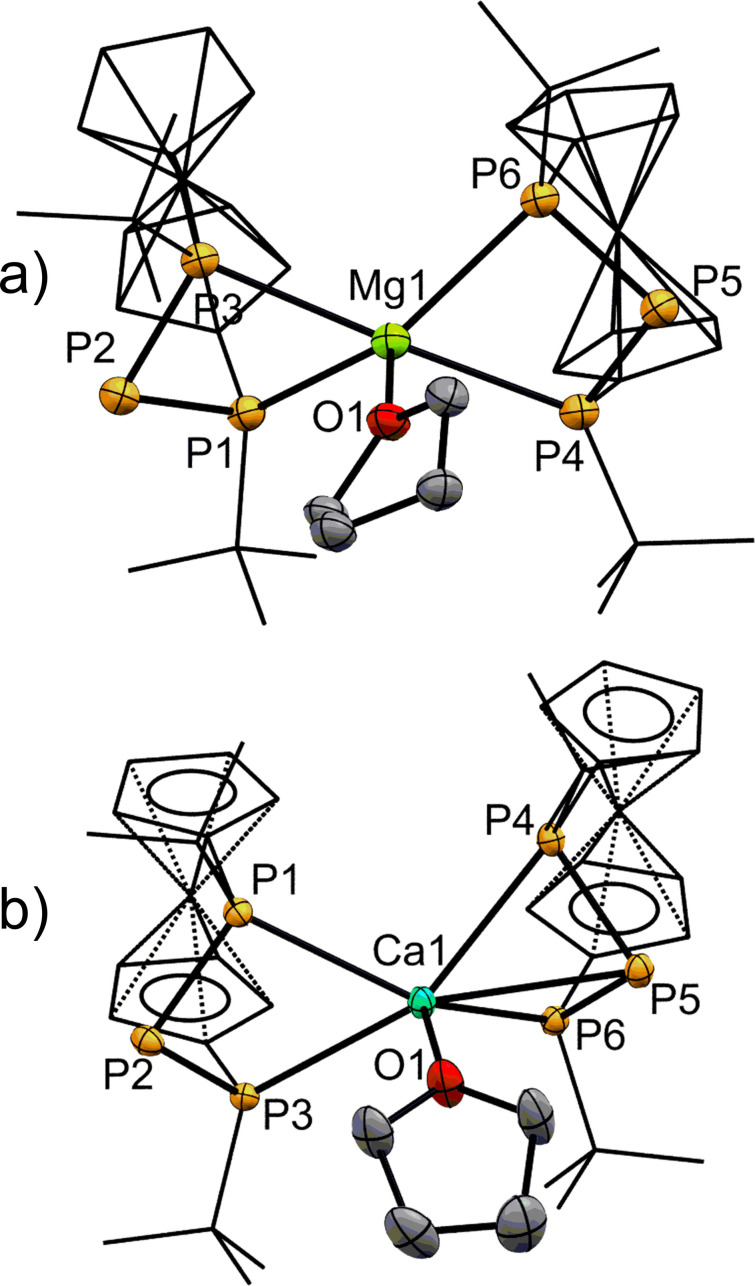
ORTEP‐depiction of a) **2‐Mg(THF)** and b) **2‐Ca(THF)**. Ferrocenylene‐units and *t*Bu‐groups are shown in wireframe‐style. The thermal ellipsoids are represented at a 30 % probability level. a) One co‐crystallising lattice THF molecule is omitted. Selected bond lengths [Å] and angles [°]: P1−Mg1 2.686(2), P2−Mg1 3.3372(8), P3−Mg1 2.724(2), P1−P2 2.1510(13), P2−P3 2.1556(12), P1−P2−P3 83.93(5). b) A second molecule with similar bonding parameters and hydrogen atoms are omitted for clarity. Selected bond lengths [Å] and angles [°]: P1−Ca1 2.8752–2.9093(11), P2−Ca1 3.8552–3.9127(11), P3−Ca1 2.9566–2.9644(11), P4−Ca1 2.9030–2.9233(11), P5−Ca1 3.0632–3.0932(11), P6−Ca1 2.8344–2.8364(10), P1−P2 2.1673–2.1704(13), P2−P3 2.1684–2.1733(10), P1−P2−P3 86.45–87.22(4), P4−P5−P6 87.23–89.13(4).

For **2‐Mg** the same structural motif as in **2‐Zn** is observed, when **2‐Mg** is recrystallized from apolar cyclopentane (Figure 3b). The central P‐atoms have only slightly different distances to the central Mg^2+^‐ion (P2–Mg1 3.2050(13) and P5–Mg1 3.2597(13)) both of which are below the sum of the respective van der Waals‐radii (∑_vdW_=441 pm),^[48]^ but do not connote chemical bonding. A recrystallization of **2‐Mg** from THF entails incorporation of the donor solvent in the coordination sphere of the Mg^2+^‐ion in addition to the phosphanide, resulting in a distorted square pyramidal geometry with pentacoordination at the central atom for **2‐Mg(THF)** (Figure 4a). Again, none of the formal phosphanide centers are involved in bonding to the central atom. When comparing **2‐Mg** with **2‐Mg(THF)**, it is notable that P−Mg bond lengths are on average 20 Å longer in the THF‐adduct owing to the higher coordination number.[^38^, ^49^] Nevertheless, all P−P and P−Mg bond lengths are in expected ranges.[^31^, ^38^]

For the next heavier Ae‐metal, calcium, the solid‐state structure confirms coordination of THF in addition to the two triphosphanide anions to the central calcium ion in **2‐Ca(THF)**, consistent with the previously discussed findings in solution based on NMR. In contrast to the structurally related **2‐Mg(THF)**, in **2‐Ca(THF)** the terminal P‐atoms form slightly asymmetric contacts to the Ca^2+^‐ion. The P−Ca bond lengths are ranging from 2.8344–2.9644(11) Å and are in line with other calcium phosphan(id)es.[^35^, ^41^, ^50^, ^51^] A more interesting feature is the P5−Ca1 contact, which is significantly shorter than the P2−Ca1 distance of the other triphosphanyl‐moiety (see Figure 4b). While both distances are below the sum of the van der Waals‐radii of calcium and phosphorus (∑_vdW_=452 pm),^[48]^ the latter distance may only point to a weak electrostatic or van der Waals‐interactions.^[48]^ Hence the calcium ion is sixfold coordinated, as it is in most of its complexes, however, in a quite uncommon geometry which has to be attributed to the predefined sterics of the [3]‐ferrocenophane scaffold. As a result of the additional P5−Ca1 bond, both the P4−P5−P6 angle and the P4−Ca1−P6 angle of 63.18–64.18(3)° slightly widen compared to the opposing, two‐times coordinating bisphosphanylphosphanido‐moiety (P1−Ca1−P3 60.94–61.58(3)°).

**Figure 5:**
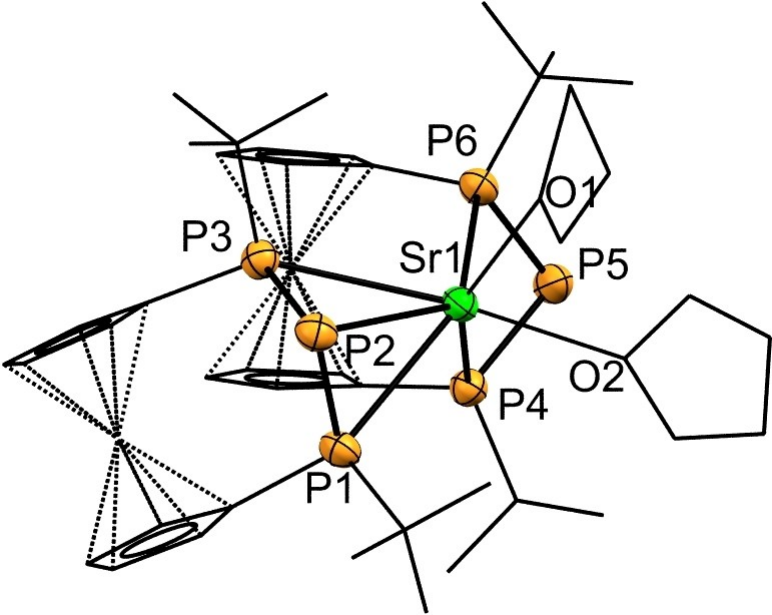
ORTEP‐depiction of **2‐Sr(THF)_2_** with hydrogen atoms omitted for clarity. Ferrocenylene‐units, *t*Bu‐groups and THF‐ligands are shown in wireframe‐style. The thermal ellipsoids are represented at a 30 % probability level. Selected bond lengths [Å] and angles [°]: a) P1−Sr1 3.058(2), P2−Sr1 3.332(2), P3−Sr1 3.139(2), P4−Sr1 3.148(2), P5−Sr1 4.039(2), P6−Sr1 3.133(2), P1−P2 2.170(3), P4−P5 2.171(3), P1−P2−P3 90.00(11), P4−P5−P6 87.12(11).

**Figure 6:**
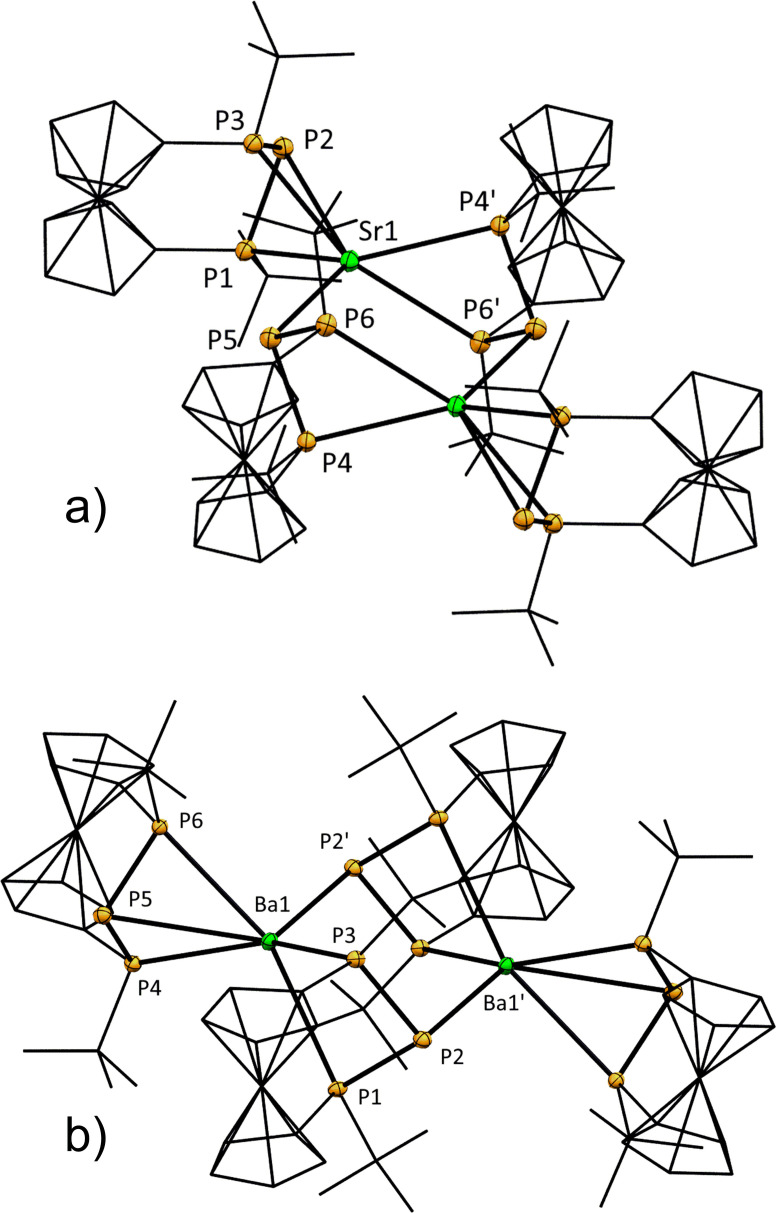
ORTEP‐depiction of a) **2‐Sr** and b) **2‐Ba**. Hydrogen atoms are omitted for clarity. Ferrocenylene‐units and *t*Bu‐groups are shown in wireframe‐style. The thermal ellipsoids are represented at a 30 % probability level. Selected bond lengths [Å] and angles [°]: a) P1−Sr1 3.0171(15), P2−Sr1 3.3281(15), P3−Sr1 3.0385(15), P4’−Sr1 3.1789(15), P5−Sr1 3.1753(15), P6’−Sr1 3.3070(15), P1−P2 2.180(2), P1−P2−P3 89.77(7). b) P1−Ba1 3.4467(10), P2’−Ba1 3.3073(10), P3−Ba1 3.3105(10), P4−Ba1 3.1327(10), P5−Ba1 3.3992(10), P6−Ba1 3.1720(10), P1−P2 2.1880(14), P1−P2−P3 89.66(5).

As outlined before, formation of **2‐Sr** according to the route shown in Scheme 1 required the presence of coordinating solvents such as THF. In the above section devoted to the NMR spectral characterization of the isolated product, the ^1^H NMR spectrum in toluene‐*d*_8_ solution indicates the composition **2‐Sr(THF)** containing one THF molecule per metal ion. However, our attempts to obtain single crystals of **2‐Sr(THF)** suitable for X‐ray diffraction were only successful using THF as solvent. Interestingly, for this crop of crystals the molecular structure in the solid state indicates a composition **2‐Sr(THF)_2_** with two THF molecules per strontium ion (Figure 5). Despite obvious similarities of **2‐Sr(THF)_2_** with previously discussed **2‐Ca(THF)**, the Sr^2+^‐ion is sevenfold coordinated by two THF molecules and five P‐atoms. In the solid state, the two triphosphanide‐moieties form asymmetric contacts to the metal ion. While the Sr1−P5 distance of 4.039(2) Å is closely below the sum of the van der Waals radii (∑_vdW_=474 pm),^[48]^ it does not connote a chemical bond unlike the opposing Sr1−P2 distance (3.332(2) Å) of the other triphosphanide‐moiety. According to the CSD,^[52]^ P−Sr distances cover a range between 2.974–3.278 Å depending on the coordination number which is six in most cases.[^31^, ^35^, ^36^, ^37^, ^41^] In consequence, the Sr1−P2 bond of **2‐Sr(THF)_2_** features the longest Sr−P bond so far reported. In analogy to **Sr(THF)_2_**, we obtained single crystals of the corresponding **2‐Ba(THF)_2_** from THF solution. In **2‐Ba(THF)_2_** the Ba^2+^‐ion has a coordination number of eight featuring tridentate coordination of both triphosphanide ligands. Owing to the poor crystal quality further details of the SCXRD of **2‐Ba(THF)_2_** will not be discussed here but have been made available in the Supporting Information (Figure S40b). Although we were unsuccessful in obtaining **2‐Sr** in donor‐free non‐polar solvents on a macroscopic scale, it was possible to attain single crystals of **2‐Sr** in trace amounts from toluene. Regardless of the different ionic radii of strontium and barium ions, compounds **2‐Sr** and **2‐Ba** are isomorphous and isostructural in the solid state, as observed before elsewhere.^[38]^ They crystallize as dimers with an inversion center on the virtual M−M bond axis. Both metal atoms are hexacoordinated with one P_3_‐unit coordinating in a tridentate, a second one in a bidentate and a third, neighbouring unit binding in a monodentate manner (see Figure 6a, b). In consequence, the geometry at the metal atoms is quite unusual and cannot be described by common coordination polyhedra. Selected bond lengths are presented in Table 2. Compound **2‐Sr** features bond lengths over a wide range with the Sr1−P2 bond constituting another example of an exceptionally long Sr–P bond. When comparing the bond lengths of **2‐Sr** and **2‐Sr(THF)_2_**, it is notable that the increased coordination number leads to a slight lengthening of the P−Sr bonds of about 0.1 Å which is consistent with literature.[^38^, ^49^]

Similarly, in compound **2‐Ba** (Figure 6b) the Ba−P bond lengths cover a wide range with the P1−Ba1 contact of 3.4467(10) Å being the longest so far reported for five‐ and sixfold coordinated barium phosphanides. Only a single sevenfold coordinated complex had been reported in which this value is exceeded, albeit with limited quality of structure refinement.^[37]^ It is quite common, that closely related strontium and barium complexes are isostructural with completion of the larger coordination sphere being achieved via dimerization.[^35^, ^36^, ^38^, ^49^] Besides the increasing size and softness of the heavier cations, dispersive interaction may contribute to the dimer formation as has been demonstrated for a *t*Bu‐substituted secondary bisphosphanylferrocene very recently.^[53]^

The heavier Ae‐metal phosphanides demonstrate that all P‐atoms of the P_3_‐unit can act as donor sites, in contrast to **2‐Mg**, **2‐Zn** and **2‐Mg(THF)** where this is not observed. Interestingly, *μ*_2_‐bridging of the formal central phosphanide atoms, which has served especially for coordinative saturation of the heavier alkaline earth metal ions,[^31^, ^32^, ^36^] cannot be found for any of the here presented compounds. This may be attributed to the low basicity of the formal phosphanide center^[54]^ or steric constraints imposed by the ferrocenophane‐scaffold, as chain‐structured coordination polymers were primarily observed for smaller phosphanide ligands.^[41]^

### Coinage Metal Phosphanides

**Scheme 2:**
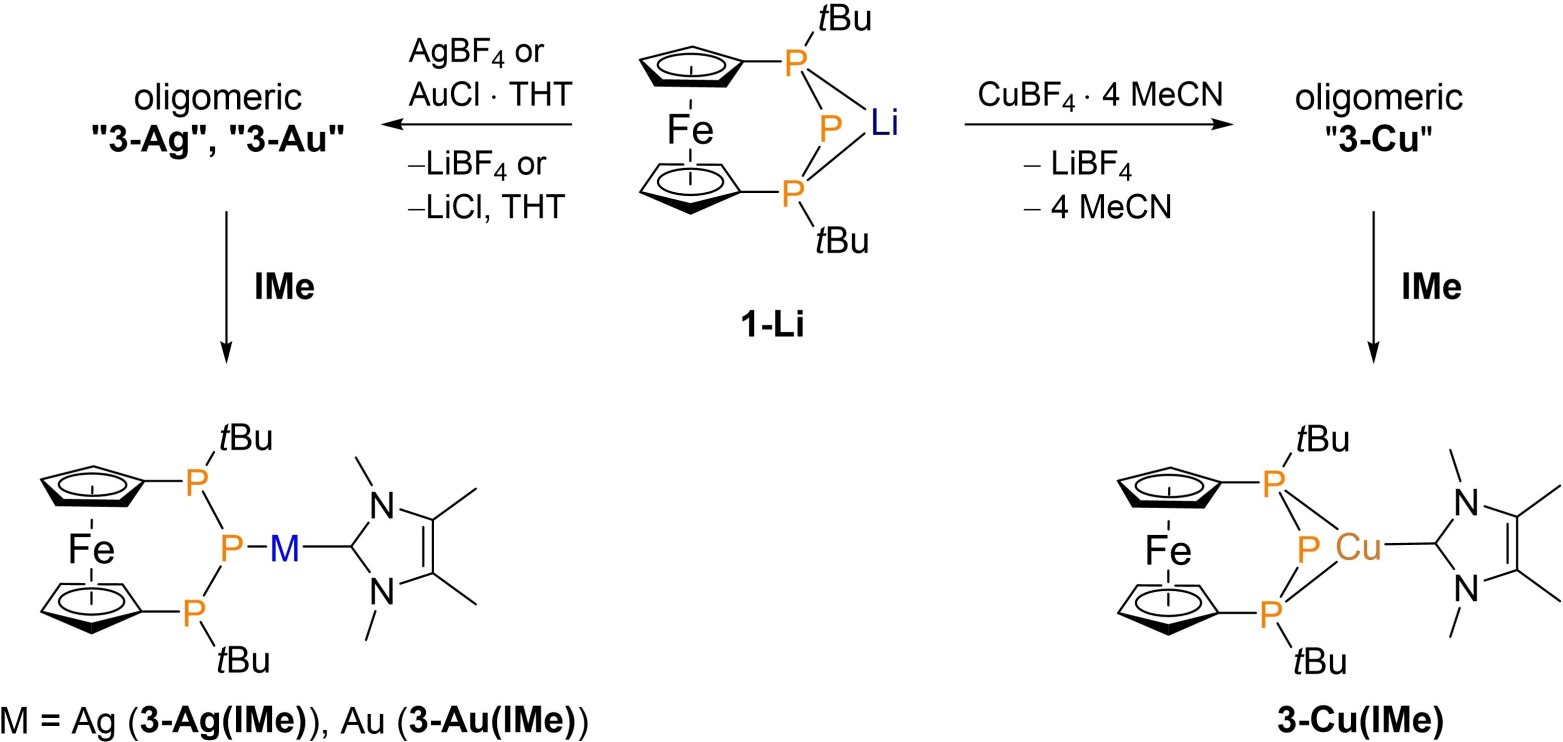
Synthetic access to **3‐Cu(IMe)**, **3‐Ag(IMe)** and **3‐Au(IMe)** via metathesis reaction of **1‐Li** and a suitable metal salt and subsequent disaggregation by **IMe**.

Based on our results obtained for the heavier Ae‐metal phosphanides **2‐Sr(THF)** and **2‐Ba**, we were wondering how metal cations with a more pronounced soft Lewis acidic character such as the monovalent coinage metal ions would behave.[^55^, ^56^, ^57^] To investigate this matter, **1‐Li** was reacted with CuBF_4_ ⋅ 4 MeCN, AgBF_4_ and AuCl ⋅ THT in ethereal solvents, respectively (see Scheme 2). In all three cases, precipitation occurred. The solid proved to be hardly soluble in any solvent, giving rise to very broad NMR signals (see Figure S31). Based on these observations and examples from the literature,^[58]^ the formation of several oligomeric and polymeric species can be assumed. In one instance, crystallisation of a trace amount of the dimer **(3‐Au)_2_** was possible (see Figure S40), thereby corroborating this hypothesis. As there is precedence that such oligomers can be transformed to monomeric species upon donor addition,^[59]^ we employed the NHC 1,3,4,5‐tetramethylimidazol‐2–ylidene (**IMe**) as a strong, unhindered σ‐donor for this purpose. Gratifyingly, this approach turned out to be successful for our complexes **3‐M** (M=Cu, Ag, Au) and best results were obtained using less than one equivalent of **IMe** avoiding separation issues with unreacted NHC. It must be noted that the resulting compounds **3‐M(IMe)** are prone to hydrolysis and sensitive to air and reactive solvents such as dichloromethane, unlike related examples.[^15^, ^60^] Furthermore, **3‐Ag(IMe)** slowly decomposes under inert condition and in the presence of light under extrusion of elemental silver along with the previously published bis‐[3]ferrocenophane containing a central P_6_‐unit which has been identified via its characteristic ^31^P NMR pattern.^[61]^ Somewhat related to this, we observed formation of **1‐H** in our attempts to investigate the electrochemistry of the here reported triphosphanide complexes. **1‐H** features a redox potential consistent with the previously published value and has been identified via ^31^P NMR spectroscopy after cyclic voltammetry measurements as well. In previous work, the occurrence of **1‐H** has been observed upon electrochemical oxidation of the above mentioned P_6_‐bis‐[3]ferrocenophane,^[61]^ but alternatively may have formed via hydrolysis in our case.

Each of the above mentioned coinage metal complexes was investigated by ^1^H, ^13^C and ^31^P NMR spectroscopy. Selected NMR data of **3‐Cu(IMe)**, **3‐Ag(IMe)** and **3‐Au(IMe)** are displayed in Table 3. Notably, **3‐Cu(IMe)** shows the largest difference in chemical shifts of the terminal and the central P‐atoms |Δ(δP_central_‐δP_terminal_)| as well as the largest value of the ^1^ *J*_PP_ coupling constant. Both characteristics are similar to those of the alkali(ne earth) metal phosphanides (see Table 1).^[20]^ In previous work, the value of the ^1^ *J*_PP_ coupling constant has proven being a good indicator for whether the metal is coordinated in mono‐ or bidentate fashion by a bisphosphanylphosphanido‐ligand.[^18^, ^21^] The drastic difference in the value of the ^1^ *J*_PP_ coupling constant for **3‐Cu(IMe)** versus **3‐Ag(IMe)** and **3‐Au(IMe)** indicates a different coordination mode of the bisphosphanylphosphanido‐ligand at the metal. It can be anticipated that in this series of coinage metal complexes the copper complex **3‐Cu(IMe)** features the most polarized M−P bonds,^[62]^ comparable to the strongly polarized situation in the alkaline (earth) metal phosphanides. As discussed above in the context of **2‐Zn** and the alkali metal analogues, negative hyperconjugation within the P−P−P‐scaffold entails slightly shortened P−P bond lengths (*vide infra*) concomitant with an increase in the value of the ^1^ *J*_PP_ coupling constant. By contrast, the Au−P bond is more covalent, owing to the small difference in electronegativity,^[63]^ resulting in a different coordination mode along with a significantly reduced value of the ^1^ *J*_PP_ coupling constant. The silver complex behaves like the gold complex, in terms of coordination mode and ^1^ *J*_PP_ coupling.

**Table 3:** Important parameters derived from the NMR‐spectroscopic analysis of **3‐Cu(IMe)**, **3‐Ag(IMe)** and **3‐Au(IMe)**.

	**3‐Cu(IMe)**	**3‐Ag(IMe)**	**3‐Au(IMe)**
δ^31^P(central) [ppm]	−132.5	−118.8	−92.3
δ^31^P(terminal) [ppm]	25.1	12.0	9.0
|^1^ *J*_PP_| [Hz]	270	165	148
|Δ(δP_central_‐δP_terminal_)| [ppm]	157.6	130.8	101.3
δ of carbene‐C [ppm]	184	185	194

Coordination of **IMe** to the metal atom causes the signal of the carbene C‐atom to shift to higher field in the ^13^C NMR spectrum. The observed shifts are comparable to the ones of other NHC‐substituted metal phosphanides.^[64]^ Moreover, a shielding of the ^13^C NMR resonance of the carbene carbon atom has been correlated with the strength of the donor interaction in closely related systems.[^65^, ^66^]

**Figure 7:**
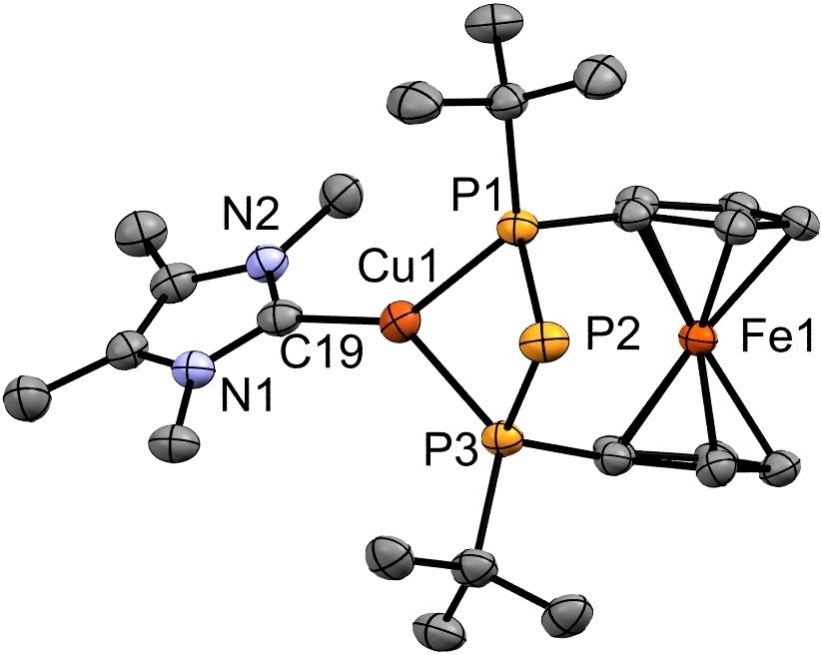
ORTEP‐depiction of **3‐Cu(IMe)**. Hydrogen atoms are omitted for clarity. The thermal ellipsoids are represented at a 30 % probability level. Selected bond lengths [Å] and angles [°]: P1−Cu1 2.3056(10), P2−Cu1 3.2848(12), P3−Cu1 2.2912(11), C19−Cu1 1.937(4), P1−P2 2.1688(15), P2−P3 2.1700(14), P1−P2−P3 80.82(5), P1−Cu1−P3 75.45(4).

In addition, the above‐mentioned phosphanides **3‐Cu(IMe)**, **3‐Ag(IMe)** and **3‐Au(IMe)** have been investigated in solid state by single crystal X‐ray diffraction. Selected bond lengths and angles are listed in Table 4. The central atom of compound **3‐Cu(IMe)** is coordinated in a distorted trigonal planar fashion, with the copper atom bonded in a bidentate chelating mode to the terminal phosphorus atoms of the triphosphanide (Figure 7). The P2−Cu1 distance is 3.2848(12) Å, exceeding a chemical bond (∑_vdW_ =462 pm).^[48]^

**Table 4:** Selected distances [Å] and bond angles [°] of **3‐Cu(IMe)**, **3‐Ag(IMe)** and **3‐Au(IMe)**. As both P1−P2 and P2−P3 bonds are of similar length, latter values are omitted.

	**3‐Cu(IMe)**	**3‐Ag(IMe)**	**3‐Au(IMe)**
P1−M1	2.3056(10)	3.4390(15)	3.412(3)
P2−M1	3.2848(12)	2.3681(15)	2.316(3))
P3−M1	2.2912(11)	3.5004(16)	3.535(3)
M1−C19	1.937(4)	2.113(6)	2.100(12)
P1−P2	2.1688(15)	2.2140(19)	2.230(4)
P1−P2−P3	80.82(5)	88.21(7)	88.64(15)

Trigonal planar coordination of copper(I) with bridging phosphanide centers is known from the literature, although linear coordination is favoured in copper(I) phosphanides with sterically demanding NHCs.[^59^, ^67^] Given the multitude of bisphosphane‐copper(I) complexes, the number of structurally characterized tricoordinate NHC adducts is surprisingly low.^[68]^ The Cu−P bond lengths are with 2.2912(11)‐2.3056(10) Å in a normal range for these compounds,[^15^, ^59^, ^67^, ^68^] with linearly coordinated copper phosphanides showing slightly shorter contacts.[^59^, ^64^] The small P−P−P bond angle of 80.82(5)° is a consequence of the short P−Cu bond lengths in comparison to the reported phosphanides (see Table 2) and the lack of interaction between the atoms P2 and Cu1. No indications for intermolecular cuprophilic interactions were found.^[69]^

**Figure 8:**
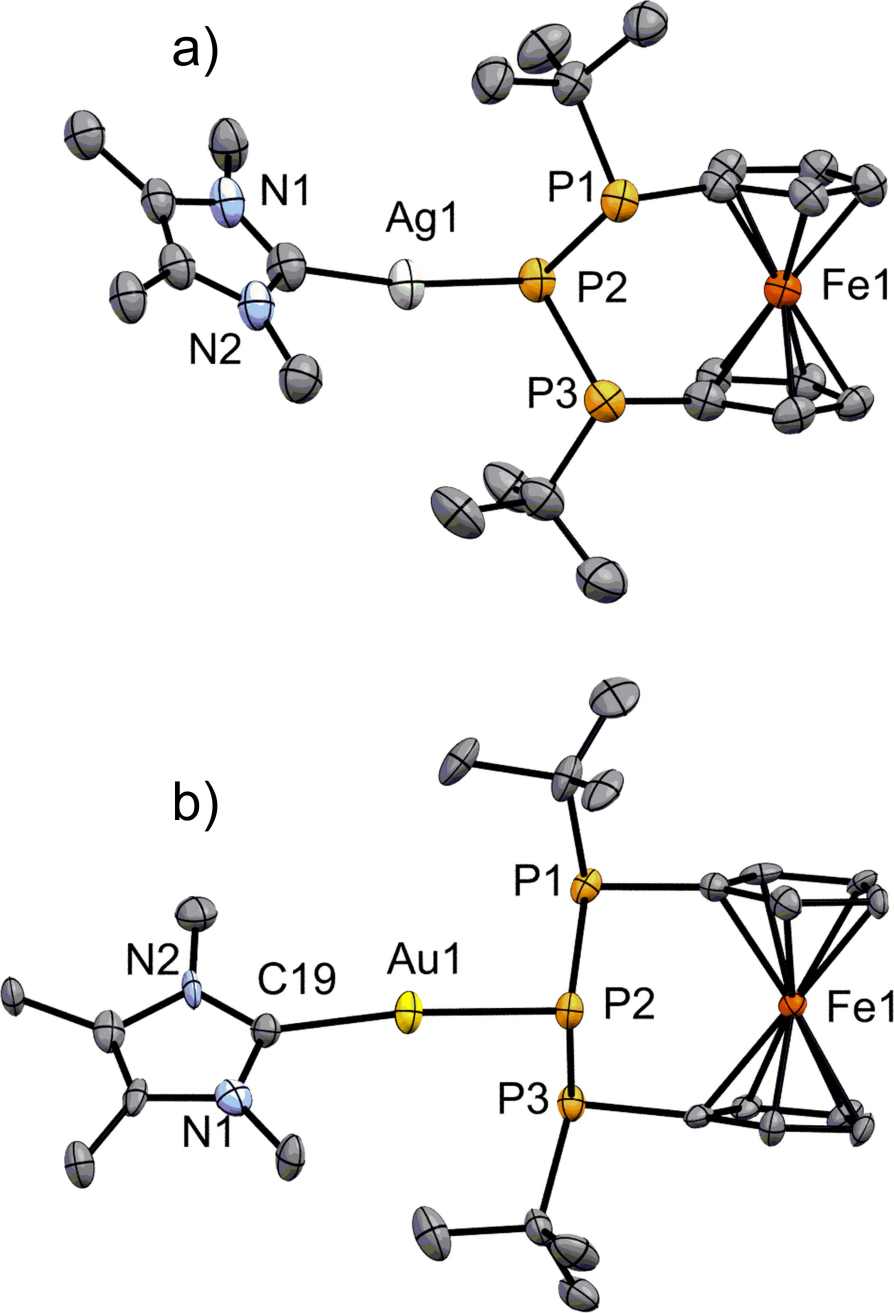
ORTEP‐depiction of a) **3‐Ag(IMe)** and b) **3‐Au(IMe)**. Hydrogen atoms are omitted for clarity. The thermal ellipsoids are represented at a 30 % probability level. Selected bond lengths [Å] and angles [°]: a) P2−Ag1 2.3681(15), C19−Ag1 2.113(6), P1−P2 2.2140(19), P2−P3 2.210(2), P1−P2−P3 88.21(7), C19−Ag1−P2 170.83(19). b) P1−Au1 3.412(3), P2−Au1 2.316(3), P3−Au1 3.535(3), C19−Au1 2.100(12), P1−P2 2.230(4), P2−P3 2.218(4), P1−P2−P3 88.64(15), C19−Au1−P2 172.0(4).

The molecular structures of **3‐Ag(IMe)** and **3‐Au(IMe)** in the solid state show a different coordination geometry than **3‐Cu(IMe)** consistent with the findings in solution based on ^31^P NMR spectra. In **3‐Ag(IMe)** and **3‐Au(IMe)** the P_3_‐ligand features monodentate coordination of the metal via the central P‐atom (Figure 8a, b). Both metal atoms are coordinated in an almost linear fashion by the NHC and the central phosphanide atom in contrast to all previously discussed complexes. While the preference of gold(I) centers to attain a linear geometry is well‐known,^[56]^ silver(I) centers prefer three‐ and fourfold coordination, with linear geometries representing a minority.[^56^, ^70^, ^71^] In fact, only very few cases with such a geometry were found featuring bisphosphane ligands, with the silver atoms then being involved in argentophilic interactions.[^72^, ^73^] No such interactions can be found for **3‐Ag(IMe)**,^[74]^ and with the average distance of the terminal P‐atoms (~3.45 Å) being only slightly under the combined van der Waals‐radii of these elements (∑_vdW_=443 pm),^[48]^ the non‐chelating coordination of the bisphosphanylphosphanido‐ligand seems quite exceptional. In line with the low‐coordination number of the silver(I) center, the P2−Ag1 bond length (2.3681(15) Å) is short, but not extraordinarily so.[^15^, ^64^, ^75^, ^76^] As a consequence of the lanthanide contraction and relativistic effects,^[77]^ gold(I) atoms are smaller than silver(I) atoms.[^56^, ^78^, ^79^] This is reflected in the shorter P2−Au1 bond length (2.316(3) Å) of **3‐Au(IMe)** which is comparable to that of other NHC‐substituted gold phosphanides.[^63^, ^80^] Despite the low steric bulk of **IMe**, no aurophilic interactions are noticeable.^[81]^ Based on their respective coordination modes, the P−P bond lengths are longer for **3‐Ag(IMe)** and **3‐Au(IMe)** than for **3‐Cu(IMe)**. In related cases, this has been attributed to the more polar Cu−P bonds,^[62]^ similar to the alkaline (earth) metal phosphanides. Interestingly, the P−P−P angles of **3‐Ag(IMe)** and **3‐Au(IMe)** are more akin to the larger alkaline earth metal phosphanides. With the presented variety of structurally characterized bisphosphanylphosphanides, it emerges that the P−P−P angle is proportional to the P−M bond length until a limit of about 89°.

With the linear Au(I) complex in hand, the steric properties of the phosphanide ligand could be determined using the *SambVca 2* web tool.[^82^, ^83^] As depicted in the steric map (see Figure S42), steric congestion is primarily imposed by the *tert*‐butyl groups, leading to a buried volume of *%V_bur_* =37.3 %. This value is relatively large compared to other phosphine ligands and falls between the values of P(C_6_F_5_)_3_ and P(*o‐*Tol)_3_ which, however, were determined using only 2.28 Å as P−Au bond length.^[84]^ A further interesting feature of **3‐Au(IMe)** is the sum of angles at the phosphanide P‐atom (289.0(3)°) which is significantly less than observed in many other gold phosphanides.[^15^, ^63^, ^64^, ^80^] The value indicates a highly pyramidal geometry and a large s‐orbital character of the remaining lone pair. The same holds true for **3‐Ag(IMe)**.

## Conclusions

The versatile coordination behaviour of the bisphosphanylphosphanido‐ligand has been explored by structural and spectroscopic means. By reacting hydrophosphane **1‐H** with alkaline earth metal bases (amide or organometallic) in a 2 : 1 ratio, the corresponding metal phosphanides **2‐Mg**, **2‐Ca(THF)**, **2‐Sr**, **2‐Sr(THF)**, **2‐Sr(THF)_2_**, **2‐Ba** and **2‐Ba(THF)_2_** are formed. The ^1^H‐DOSY NMR spectra suggest that no dimerization occurs in non‐polar solvents. If the reaction was conducted in THF, one or two THF molecules are included into the coordination sphere at the respective metal phosphanides. For comparison, zinc, and coinage metal phosphanides were prepared by a salt‐metathesis reaction of **1‐Li** and the corresponding metal salts. For the latter, disaggregation of the initially formed presumably oligo‐ or polymeric coinage metal phosphanides has been achieved with the NHC **IMe**, resulting in rare, mononuclear coinage metal phosphanide complexes **3‐Cu(IMe)**, **3‐Ag(IMe)** and **3‐Au(IMe)**. The value of the ^1^ *J*_PP_ coupling constant of the obtained complexes in solution was found to be a good probe for tracking the bonding situation, with larger values indicating chelating coordination of the metal by the terminal P‐atoms as in for example 3**‐Cu(IMe)** and smaller ones a coordination by the central phosphanide atom as in **3‐Ag(IMe)** and **3‐Au(IMe)**. Despite the bisphosphanylphosphanido‐scaffold's flexibility in adopting variable ligation modi, depending on the metal's softness, ion potential, presence of coordinating solvents or steric bulk of additional ligands, *μ*_2_‐bridging of the phosphanide center was never observed in our investigation.

## Supporting Information

The authors have cited additional references within the Supporting Information (Ref. [85–93]) alongside deposition numbers for supplementary crystallographic data (Ref. [94]).

## Conflict of Interests

The authors declare no conflict of interest.

1

## Supporting information

As a service to our authors and readers, this journal provides supporting information supplied by the authors. Such materials are peer reviewed and may be re‐organized for online delivery, but are not copy‐edited or typeset. Technical support issues arising from supporting information (other than missing files) should be addressed to the authors.

Supporting Information

## Data Availability

The data that support the findings of this study are available in the supplementary material of this article.
