# Supplementary material for: Adaptive Coordination Behavior of Bisphosphanylphosphanido‐Ligands Toward Group 2, 11 and 12 Metal Ions
Source: Chemistry. 2025 Mar 11;31(21):e202500406. doi: 10.1002/chem.202500406 (PMC11979690; doi:10.1002/chem.202500406)
Supplement: Supplementary file 1 — Supporting Information [file CHEM-31-e202500406-s001.pdf]

Chemistry–A European Journal

Supporting Information

Adaptive Coordination Behavior of Bisphosphanylphosphanido-Ligands Toward Group 2, 11 and 12 Metal Ions

Dennis Langgut, Clemens Bruhn, and Rudolf Pietschnig*

Supporting Information

Table of contents

1. Syntheses and NMR spectra 2
1.1. General considerations 2
1.2. Synthetic protocols and characterization data 2
1.2.1. Synthesis of compound **2-Mg** 2
1.2.2. Synthesis of compound **2-Ca(THF)** 5
1.2.3. Synthesis of compound **2-Sr(THF)** 8
1.2.4. Synthesis of compound **2-Ba** 11
1.2.5. Synthesis of compound **2-Ba(THF)$_2$** 14
1.2.6. Synthesis of compound **2-Zn** 17
1.2.7. Synthesis of compound **3-Cu(IMe)** 20
1.2.8. Synthesis of compound **3-Ag(IMe)** 23
1.2.9. Synthesis of compound **3-Au(IMe)** 26
2. Crystal data and refinement details 29
3. Determination of the Buried Volume 33
4. Literature 34

1. Syntheses and NMR spectra

1.1. General considerations

All reactions were carried out by means of standard Schlenk or glovebox techniques under inert gas atmosphere (argon). Solvents were dried over Na/K alloy before use and were freshly distilled under inert gas. Deuterated solvents for NMR-spectroscopy were dried and stored over molecular sieves. $Mg(CH_2SiMe_3)_2$,[1] $Ca[N(SiMe_3)_2]_2 \cdot 2THF$,[2] $ZnCl_2 \cdot TMEDA$,[3] $Sr[N(SiMe_3)_2]_2$,[2] $Ba[N(SiMe_3)_2]_2$[2] and IMe[4] were prepared according to literature procedures while other reagents were used as received without further purification. ^{1}H, ^{13}C and ^{31}P NMR-data were recorded on Jeol JNM-ECZL500 or Varian VNMRS-500 MHz spectrometers at 25°C. Chemical shifts were referenced to residual protic impurities in the solvent (^{1}H) or the deuterated solvent (^{13}C) and reported relative to external $SiMe_4$ (^{1}H, ^{13}C). NMR Spectra of heteronuclei were referenced using the Ξ-scale following IUPAC recommendations with H_3PO_4 (85%) (^{31}P) as secondary reference.[5] Elemental analyses were performed with a HEKAtech Euro EA CHNS elemental analyser. Samples were prepared in a Sn cup and analysed with added V_2O_5.

1.2. Synthetic protocols and characterization data

1.2.1. Synthesis of compound **2-Mg**

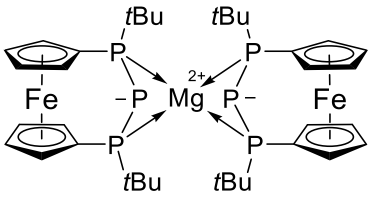

Chemical Formula: $C_{36}H_{52}Fe_2MgP_6$
Molecular Weight: 806.65

2-Mg

100 mg (0.26 mmol) of **1-H** were mixed with 26 mg (0.14 mmol, 0.5 eq.) of $Mg(CH_2SiMe_3)_2$ in 8 ml *n*-pentane. The reaction mixture was stirred for 3 days at room temperature. Afterwards, the orange suspension was centrifuged, and the solution was removed. The crude was washed by centrifugation with 5 ml *n*-pentane and then dried in vacuum. The product was obtained as orange powder. Single crystals were received by slowly evaporating a concentrated solution in THF or by cooling down a concentrated solution in cyclopentane to –35 °C.

Yield: 65 mg ≙ 62%

NMR-data [ppm]:

^{1}H NMR (500 MHz, toluene-*d*$_8$): δ 4.72 (m, 8H, Cp), 4.16 (m, 4H, Cp), 3.95 (m, 4H, Cp), 1.49-1.43 (m, 36 H, *t*Bu).

$^{13}C\{^{1}H\}$ NMR (126 MHz, toluene-*d*$_8$): δ 85.8 (d, $^{1}J_{CP}$ = 4 Hz, *ipso*-Cp), 76.7 (m, Cp), 72.5 (s, Cp), 72.1 (s, Cp), 72.1 (s, Cp), 69.1 (m, Cp), 32.7 (m, *t*Bu C_q), 30.3 (m, *t*Bu).

$^{31}P\{^{1}H\}$ NMR (202 MHz, toluene-*d*$_8$): δ 25.8 (pd, $^{1}J_{PP}$ = 290 Hz), –115.2 (pt, $^{1}J_{PP}$ = 290 Hz).

Elemental analysis [%]: Calculated: C 53.60, H 6.50; found: C 53.78, H 6.98.

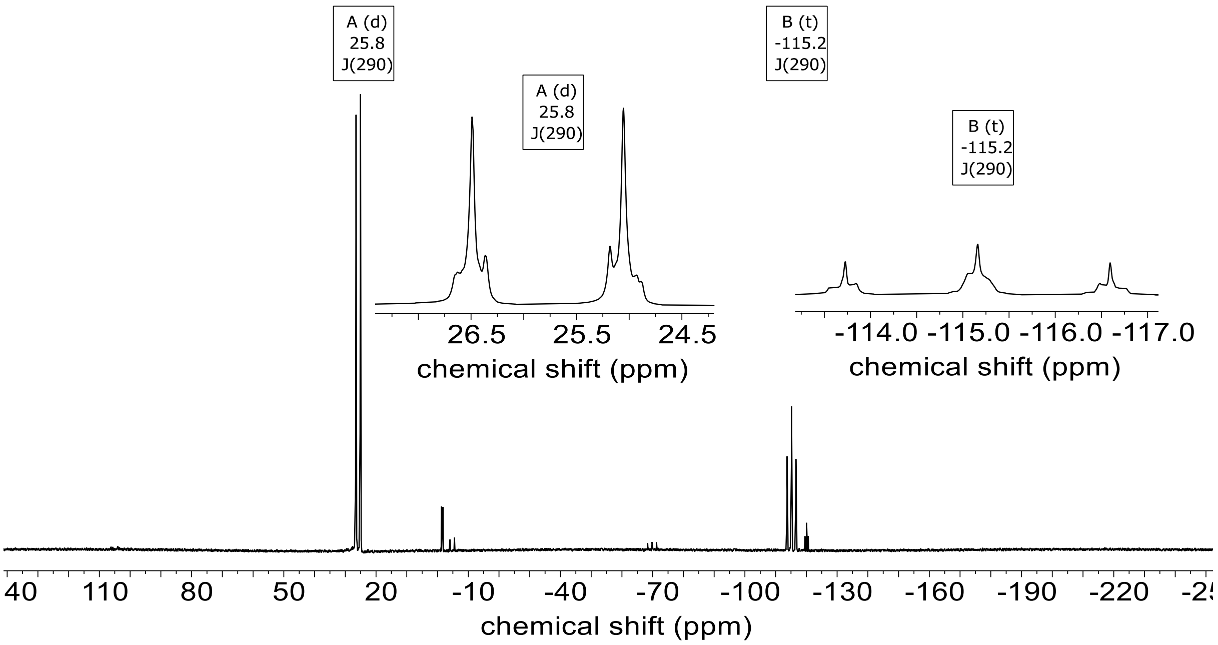

Figure S1: $^{31}P\{^1H\}$-NMR-spectrum of compound **2-Mg** recorded in toluene-d_8 at 202 MHz with the corresponding multiplets being highlighted.

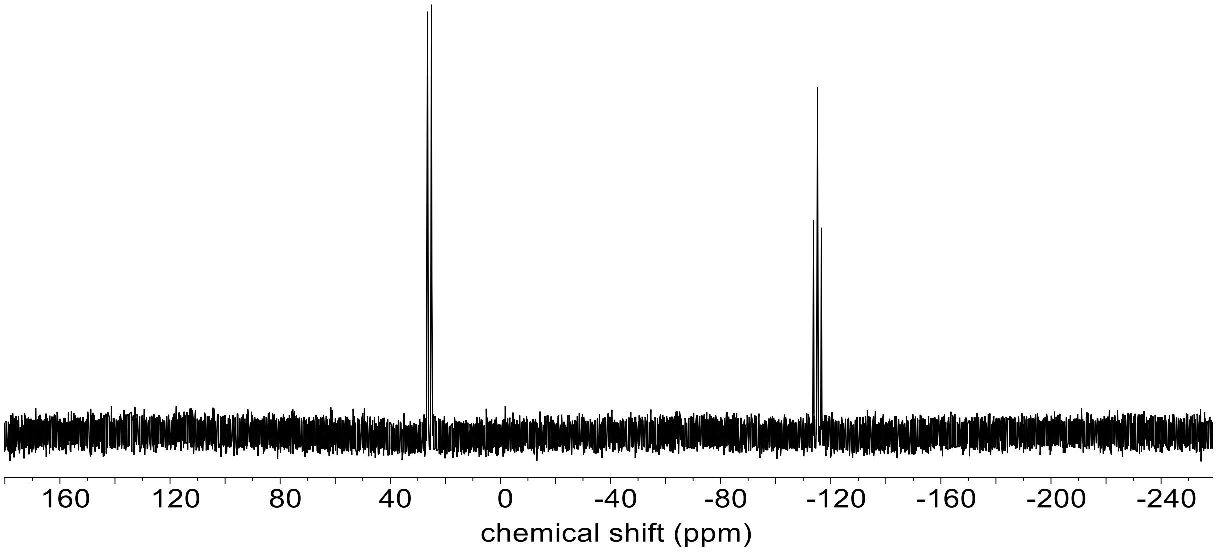

Figure S2: ^{31}P-NMR-spectrum of compound **2-Mg** recorded in toluene-d_8 at 202 MHz.

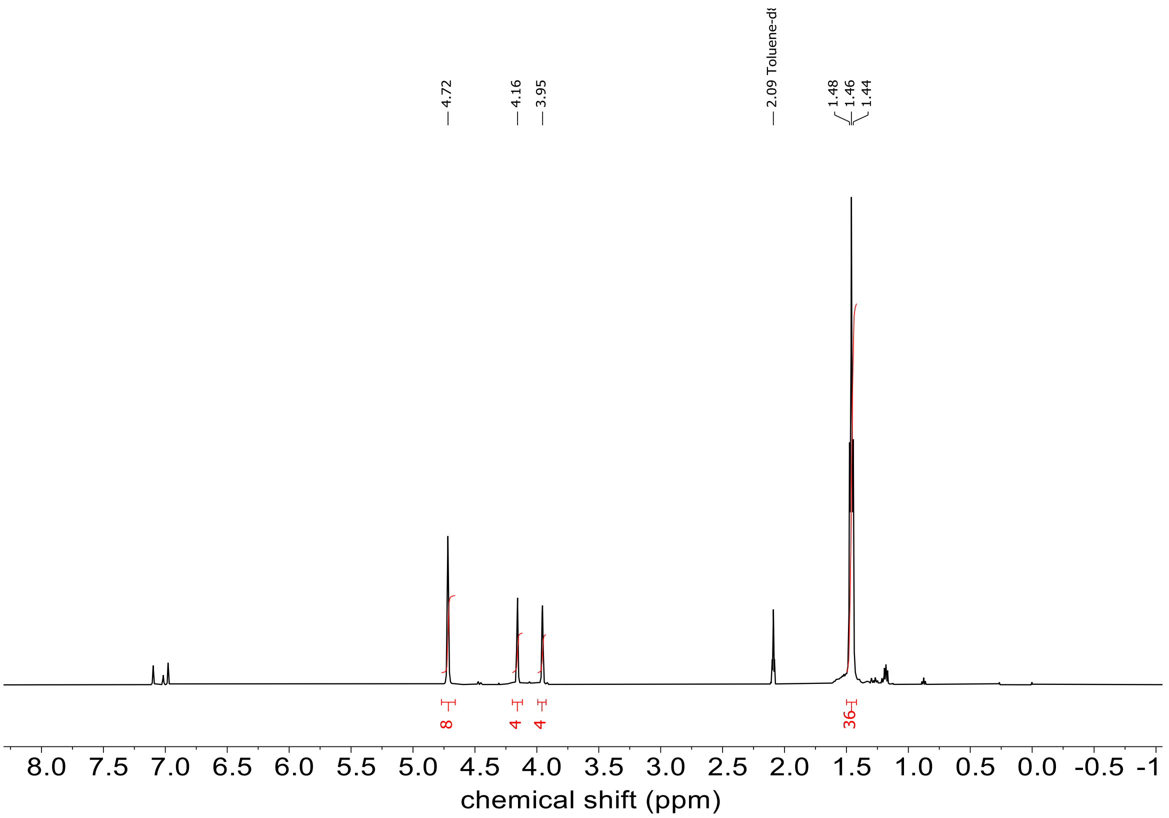

Figure S3: ^{1}H-NMR-spectrum of compound **2-Mg** recorded in toluene-d_8 at 500 MHz.

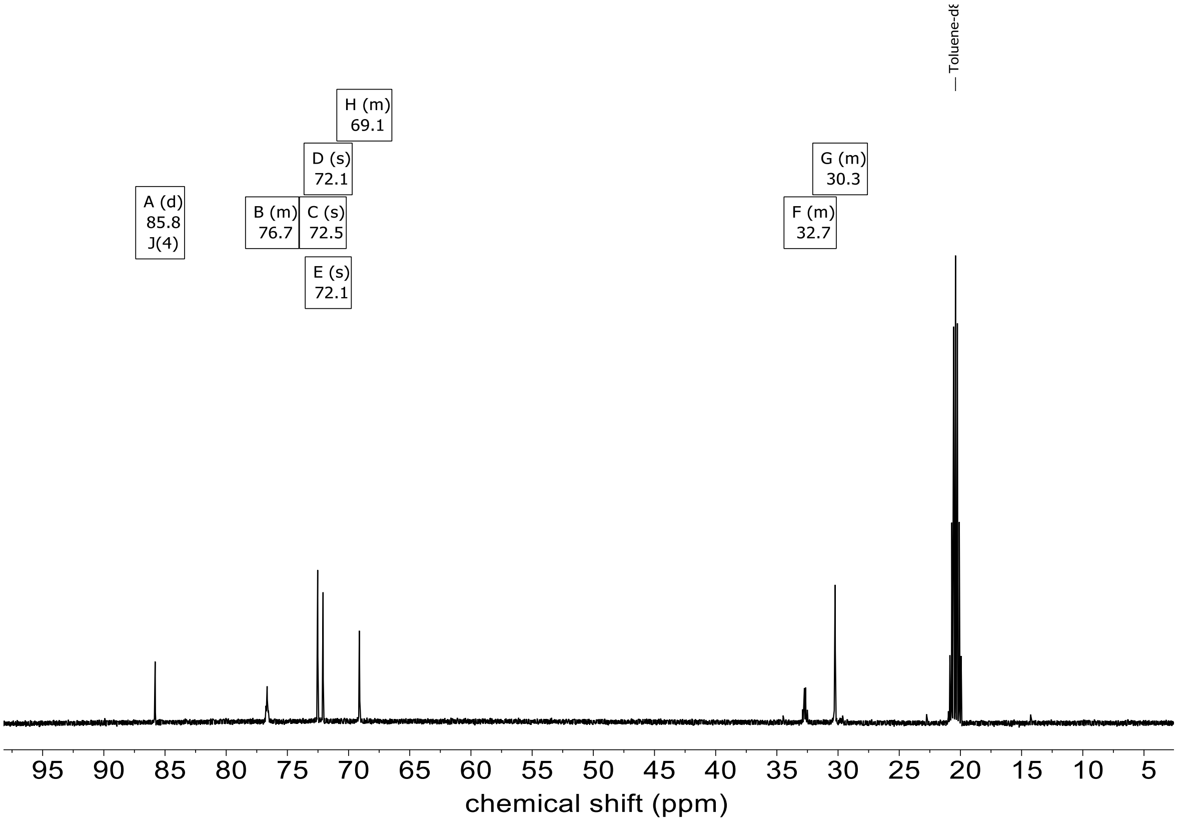

Figure S4: ^{13}C{^{1}H}-NMR-spectrum of compound **2-Mg** recorded in toluene-d_8 at 126 MHz. The remaining signals of toluene-d_8 are omitted.

1.2.2. Synthesis of compound **2-Ca(THF)**

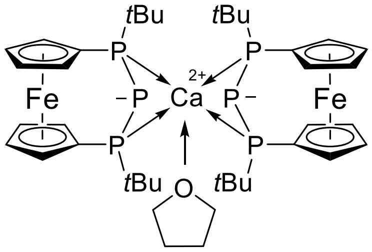

Chemical Formula: $C_{40}H_{60}CaOFe_2P_6$
Molecular Weight: 894.53

2-Ca(THF)

A Schlenk flask was charged with 100 mg (25 mmol) of **1-H**, 68 mg (13 mmol, 0.53 eq.) of $Ca[N(SiMe_3)_2]_2 \cdot 2THF$ and 7 ml *n*-pentane. The resulting suspension was stirred for 48 hours and then filtered. The filter cake was washed with 2 ml *n*-pentane and dried in vacuum afterwards. The product was obtained as an orange powder. Single crystals were received upon evaporation of a concentrated solution in toluene.

Yield: 45 mg ≙ 39%

NMR-data [ppm]:

^{1}H NMR (500 MHz, toluene-d_8): δ 5.06 (m, 4H, Cp), 4.85 (m, 4H, Cp), 4.19 (m, 4H, Cp), 4.16-4.10 (m, 4H, THF), 4.06 (m, 4H, Cp), 1.46-1.41 (m, 36 H, *t*Bu), 1.36-1.32 (m, 4H, THF).

^{13}C{^{1}H} NMR (101 MHz, toluene-d_8): δ 87.0 (m, *ipso*-Cp), 77.8 (m, Cp), 72.1 (s, Cp), 71.5 (s, Cp), 71.4 (s, Cp), 68.7 (m, THF), 31.7 (m, *t*Bu C_q), 30.4 (m, *t*Bu), 25.4 (s, THF).

^{31}P{^{1}H} NMR (202 MHz, toluene-d_8): δ 20.8 (pd, $^{1}J_{PP}$ = 270 Hz), –122.0 (pt, $^{1}J_{PP}$ = 270 Hz).

Elemental analysis [%]: Calculated: C 53.71, H 6.76; found: C 54.50, H 6.92.

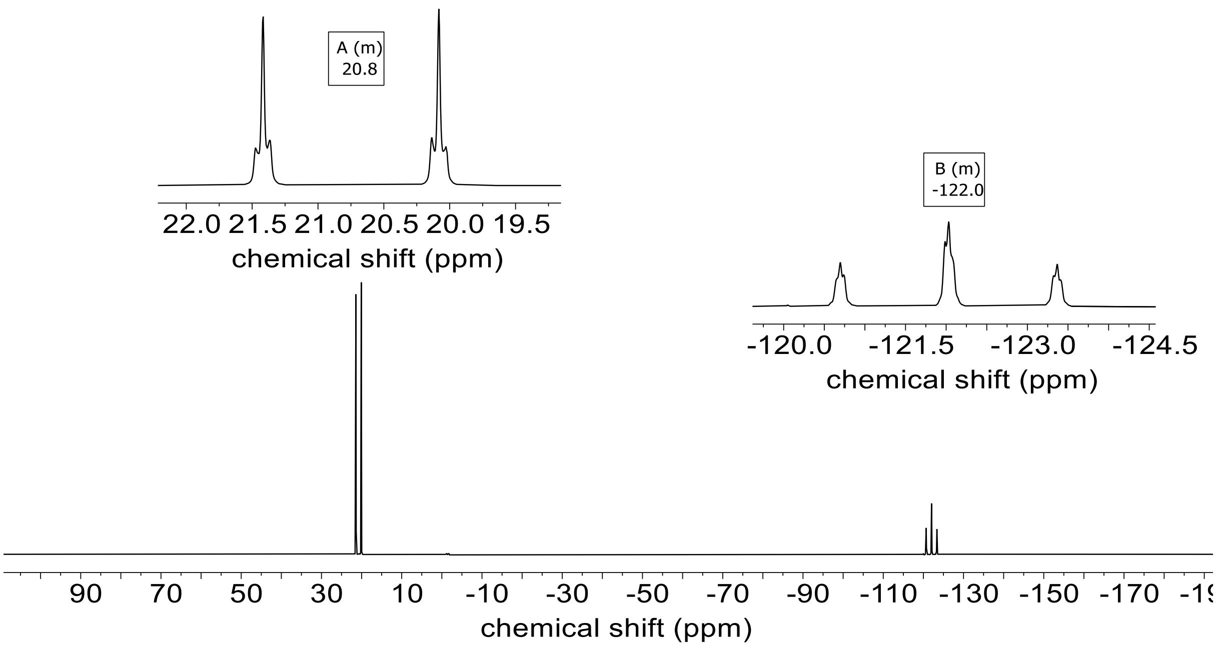

Figure S5: $^{31}P\{^{1}H\}$-NMR-spectrum of compound **2-Ca(THF)**recorded in toluene-d_8 at 202 MHz with the corresponding multiplets being highlighted.

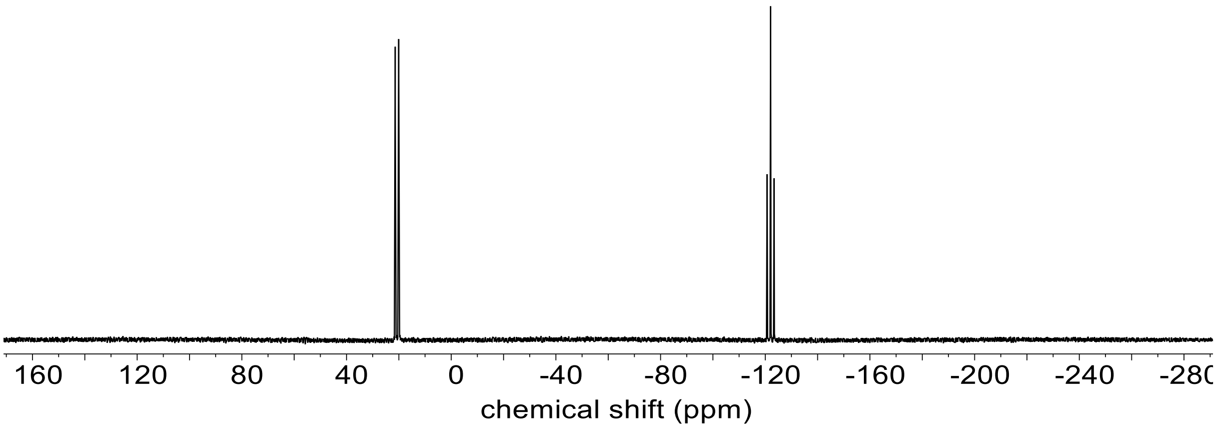

Figure S6: ^{31}P-NMR-spectrum of compound **2-Ca(THF)** recorded in toluene-d_8 at 202 MHz.

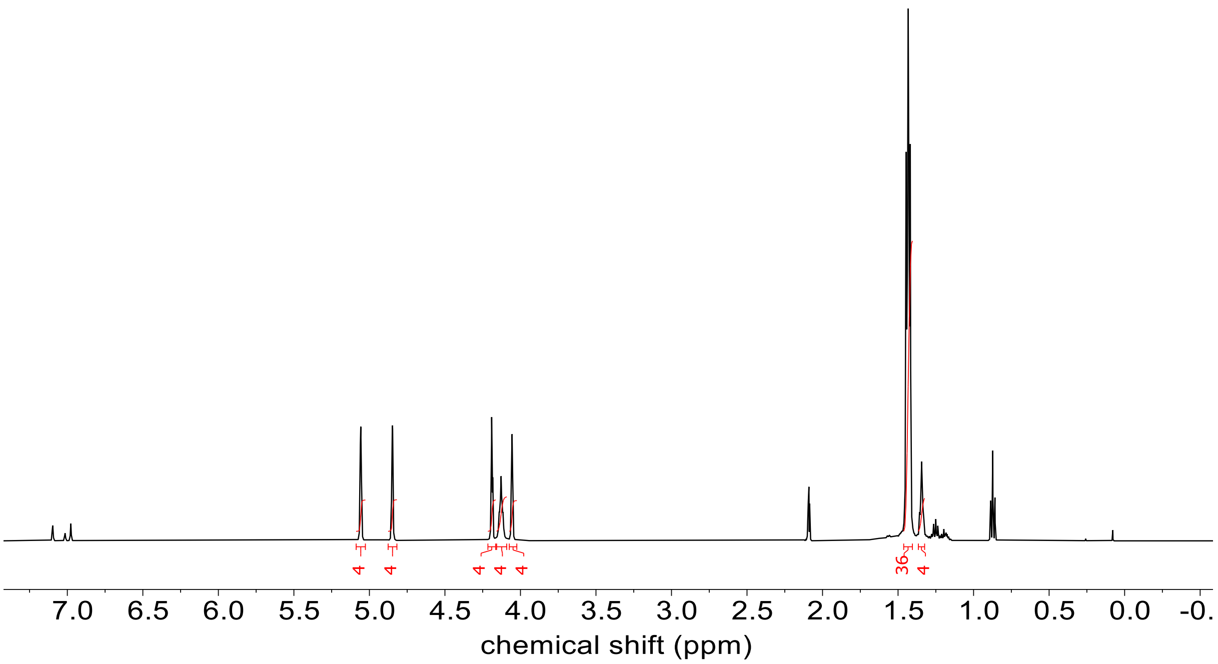

Figure S7: ^{1}H-NMR-spectrum of compound **2-Ca(THF)** recorded in toluene-d_8 at 500 MHz.

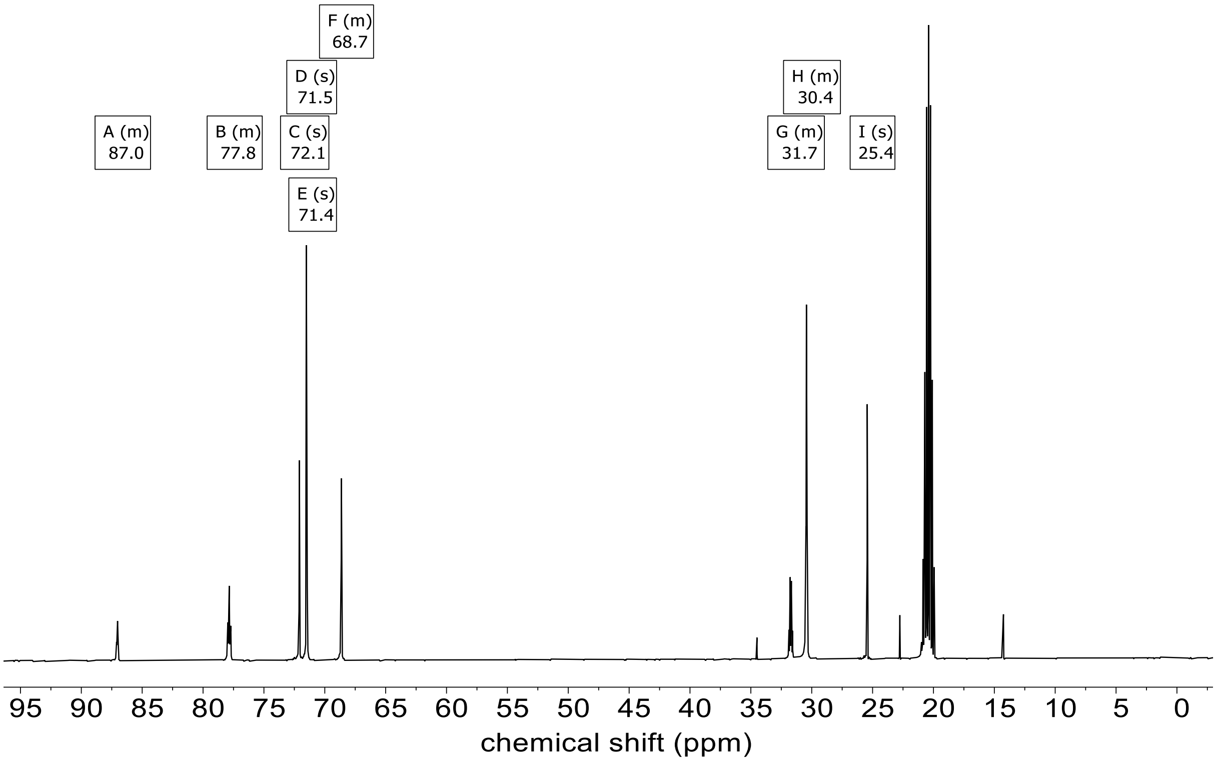

Figure S8: $^{13}C\{^1H\}$-NMR-spectrum of compound **2-Ca(THF)** recorded in toluene-d_8 at 126 MHz. The remaining signals of toluene-d_8 are omitted.

1.2.3. Synthesis of compound **2-Sr(THF)**

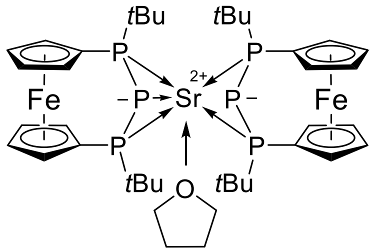

Chemical Formula: $C_{40}H_{60}SrOFe_2P_6$
Molecular Weight: 942.07

2-Sr(THF)

A Schlenk flask was charged with 81 mg (0.21 mmol) **1-H**, 43 mg (0.11 mmol, 0.51eq.) $Sr[N(SiMe_3)_2]_2$ and 5 ml THF. The reaction mixture was stirred for 4 h at room temperature and then, all volatile compounds were removed in vacuum. Subsequently, the crude was washed with 3 ml of *n*-pentane and then dried in vacuum again. The product was obtained as an orange powder. Crystallisation was achieved by overlaying a concentrated solution in THF with toluene and *n*-pentane.

Yield: 54 mg ≙ 56%

NMR-data [ppm]:

^{1}H NMR (500 MHz, toluene-d_8): δ 4.85 (m, 8H, Cp), 4.17 (m, 4H, Cp), 4.01 (m, 4H, Cp), 3.87 (m, THF), 1.45 (m, 36 H, *t*Bu), 1.36 (m, THF).

$^{13}C\{^{1}H\}$ NMR (126 MHz, toluene-d_8): δ 86.4 (m, *ipso*-Cp), 77.9 (m, Cp), 72.3 (s, Cp), 71.5 (s, Cp), 69.8 (s, Cp), 68.7 (m, THF), 31.6 (m, *t*Bu C_q), 30.4 (m, *t*Bu), 25.4 (s, THF).

$^{31}P\{^{1}H\}$ NMR (202 MHz, toluene-d_8): δ 25.1 (pd, $^{1}J_{PP}$ = 268 Hz), –121.0 (pt, $^{1}J_{PP}$ = 268 Hz).

Elemental analysis [%]: Calculated: C 51.00, H 6.42; found: C 51.28, H 6.78.

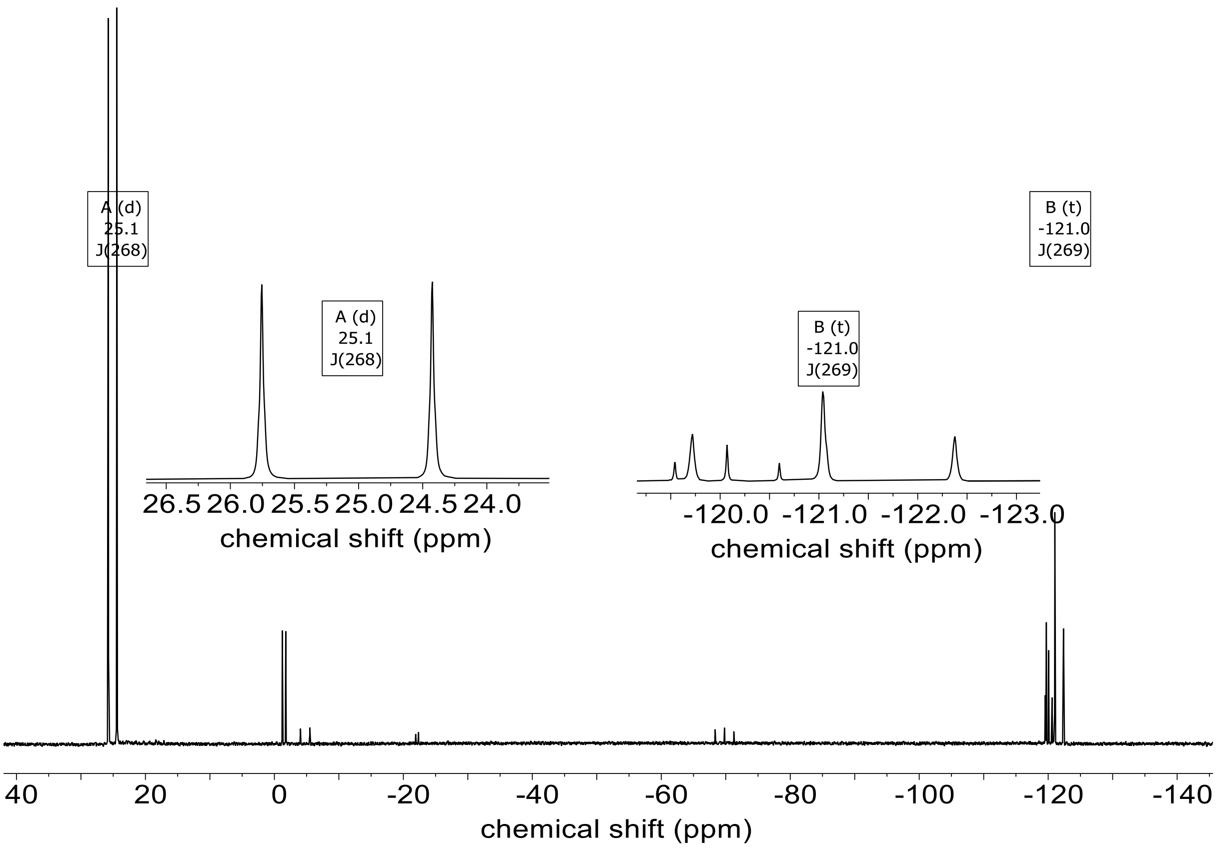

Figure S9: $^{31}P\{^{1}H\}$-NMR-spectrum of compound **2-Sr(THF)**recorded in toluene-d_8 at 202 MHz with the corresponding pseudo-doublets being highlighted.

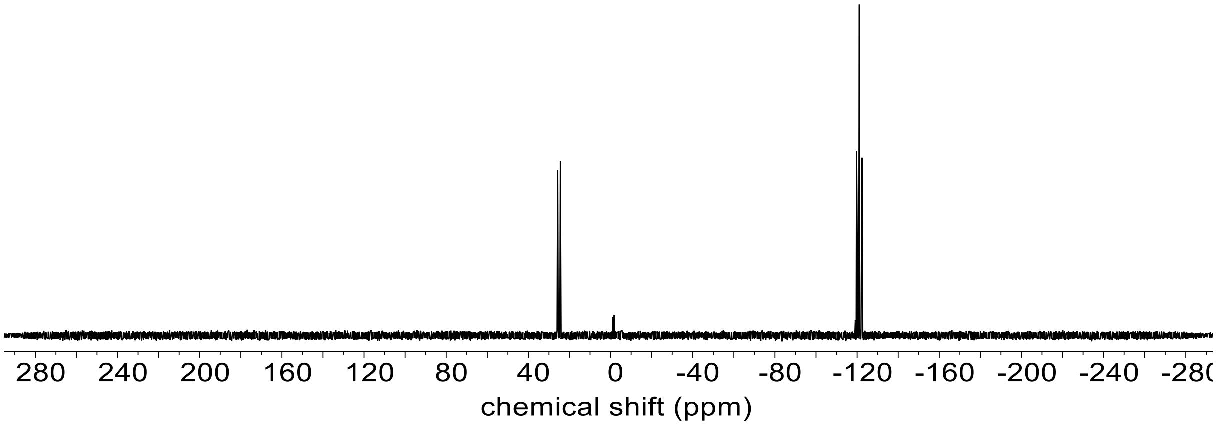

Figure S10: ^{31}P-NMR-spectrum of compound **2-Sr(THF)** recorded in toluene-d_8 at 202 MHz.

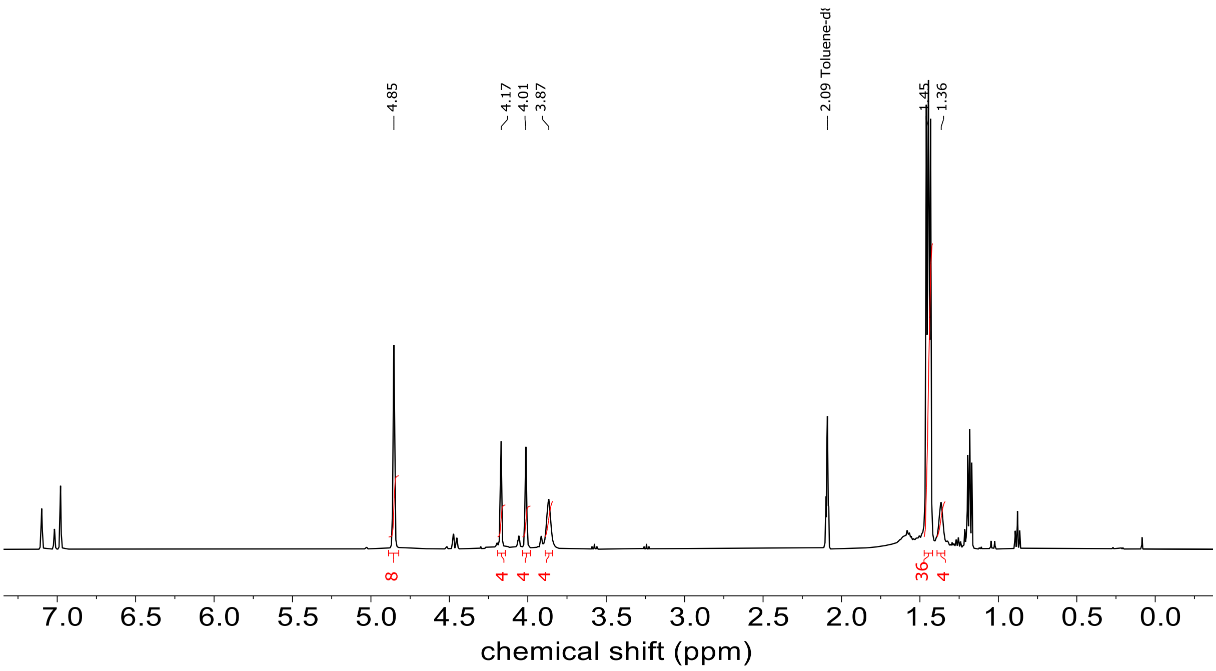

Figure S11: ^{1}H-NMR-spectrum of compound **2-Sr(THF)** recorded in toluene-d_8 at 500 MHz.

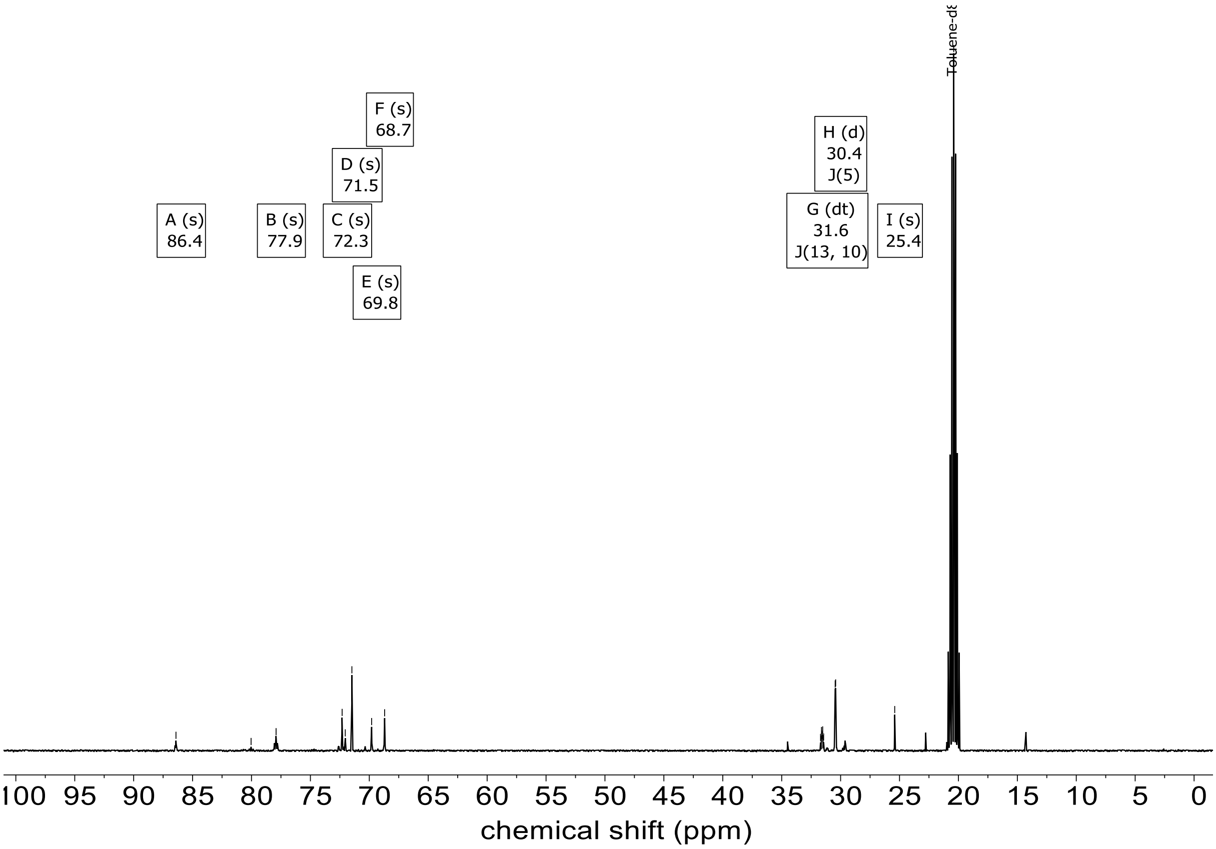

Figure S12: ^{13}C{^{1}H}-NMR-spectrum of compound **2-Sr(THF)** recorded in toluene-d_8 at 126 MHz. The remaining signals of toluene-d_8 are omitted.

1.2.4. Synthesis of compound **2-Ba**

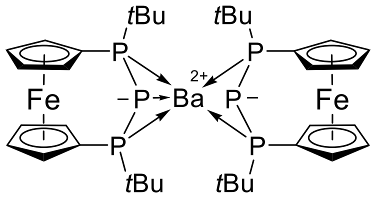

Chemical Formula: $C_{36}H_{52}BaFe_2P_6$
Molecular Weight: 919.67
2-Ba

An NMR tube was charged with 30 mg (0.07 mmol) **1-H**, 18 mg (0.04 mmol, 0.51 eq) $Ba[N(SiMe_3)_2]_2$ and 1 ml toluene. The resulting suspension was treated with ultrasonics for 5 minutes and then allowed to stand for three days. During this time, a partially crystalline precipitate formed. Subsequently, the solvent was discarded and the solid was washed with 1 ml of *n*-pentane twice. After drying in vacuum, the product was obtained as orange powder.

Yield: 22 mg ≙ 61%

NMR-data [ppm]:

^{1}H NMR (500 MHz, toluene-*d*$_8$): δ 4.82 (m, 8H, Cp), 4.14 (m, 4H, Cp), 4.01 (m, 4H, Cp), 1.37 (m, 36H, *t*Bu).

^{13}C{^{1}H} NMR (126 MHz, toluene-*d*$_8$): δ 85.4 (m, *ipso*-Cp), 78.2 (m, Cp), 72.9 (m, Cp), 71.4 (s, Cp), 68.7 (t, $^2J_{CP}$ = 4 Hz, Cp), 31.8 (m, *t*Bu C$_q$), 30.7 (q, $^2J_{CP}$ = 5 Hz, *t*Bu).

^{31}P{^{1}H} NMR (202 MHz, toluene-*d*$_8$): δ 27.5 (d, $^1J_{PP}$ = 274 Hz), –121.5 (t, $^1J_{PP}$ = 274 Hz).

Elemental analysis [%]: Calculated: C 47.02, H 5.70; found: C 46.91, H 5.65.

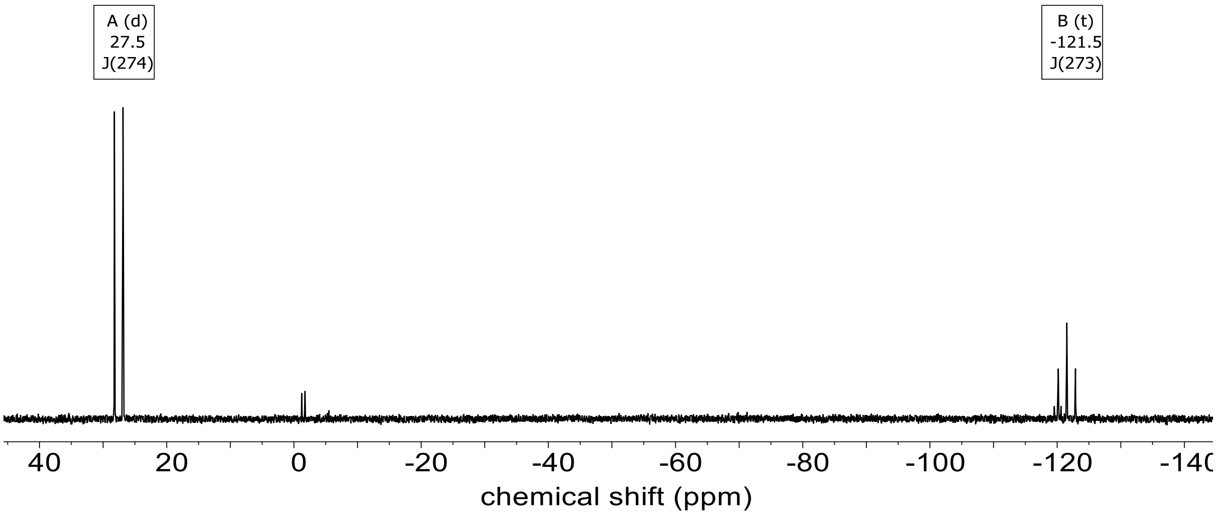

Figure S13: ^{31}P{^{1}H}-NMR-spectrum of compound **2-Ba** recorded in toluene-*d*$_8$ at 202 MHz.

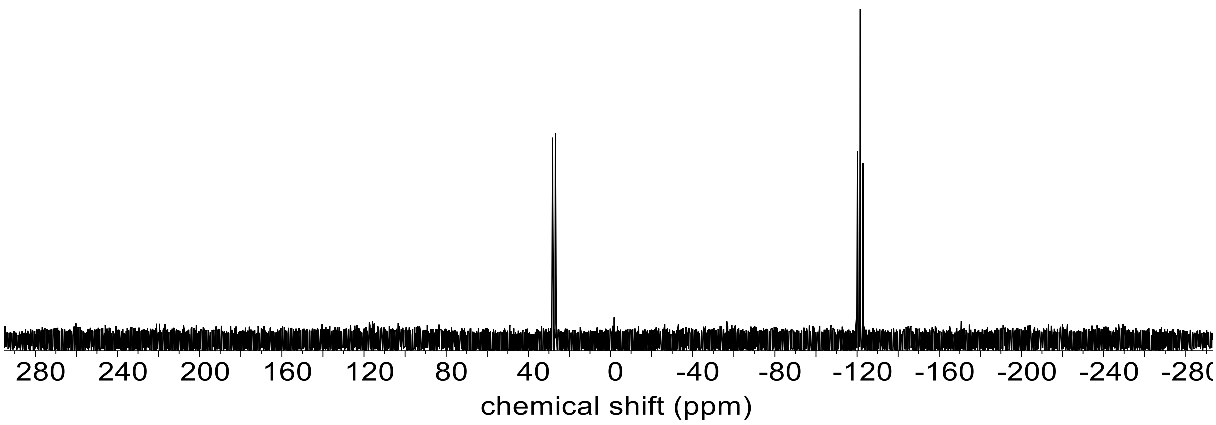

Figure S14: ^{31}P-NMR-spectrum of compound **2-Ba** recorded in toluene-d_8 at 202 MHz.

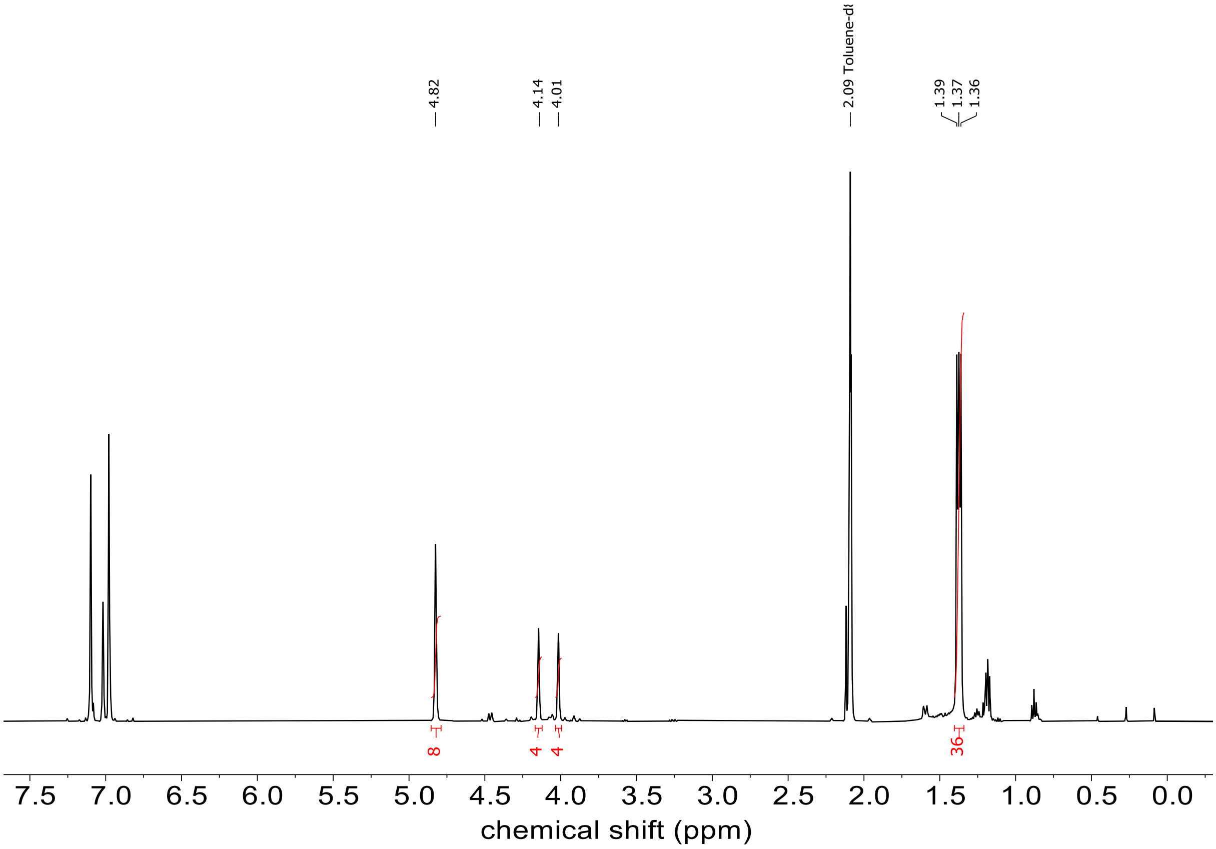

Figure S15: ^{1}H-NMR-spectrum of compound **2-Ba** recorded in toluene-d_8 at 500 MHz.

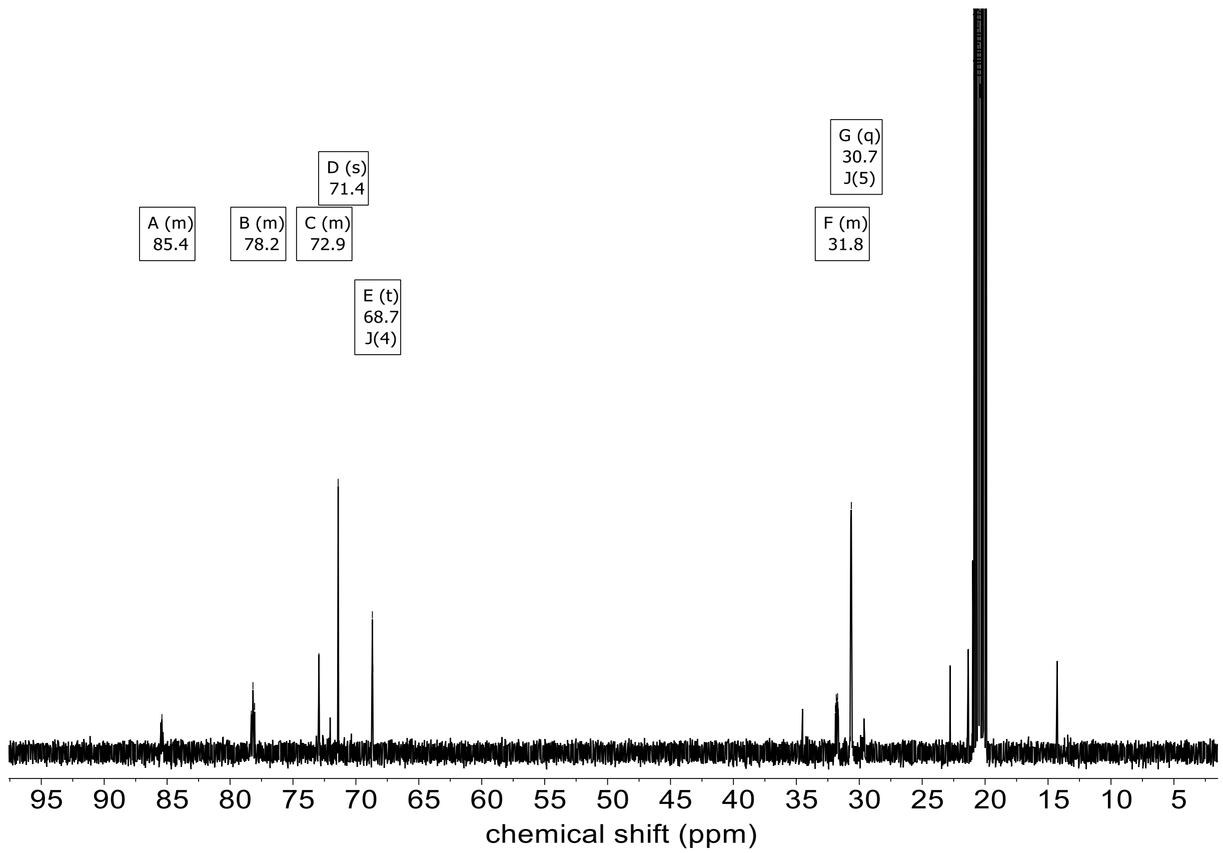

Figure S16: $^{13}C\{^{1}H\}$-NMR-spectrum of compound **2-Ba** recorded in in toluene-d_8 at 126 MHz. The remaining signals of toluene-d_8 are omitted.

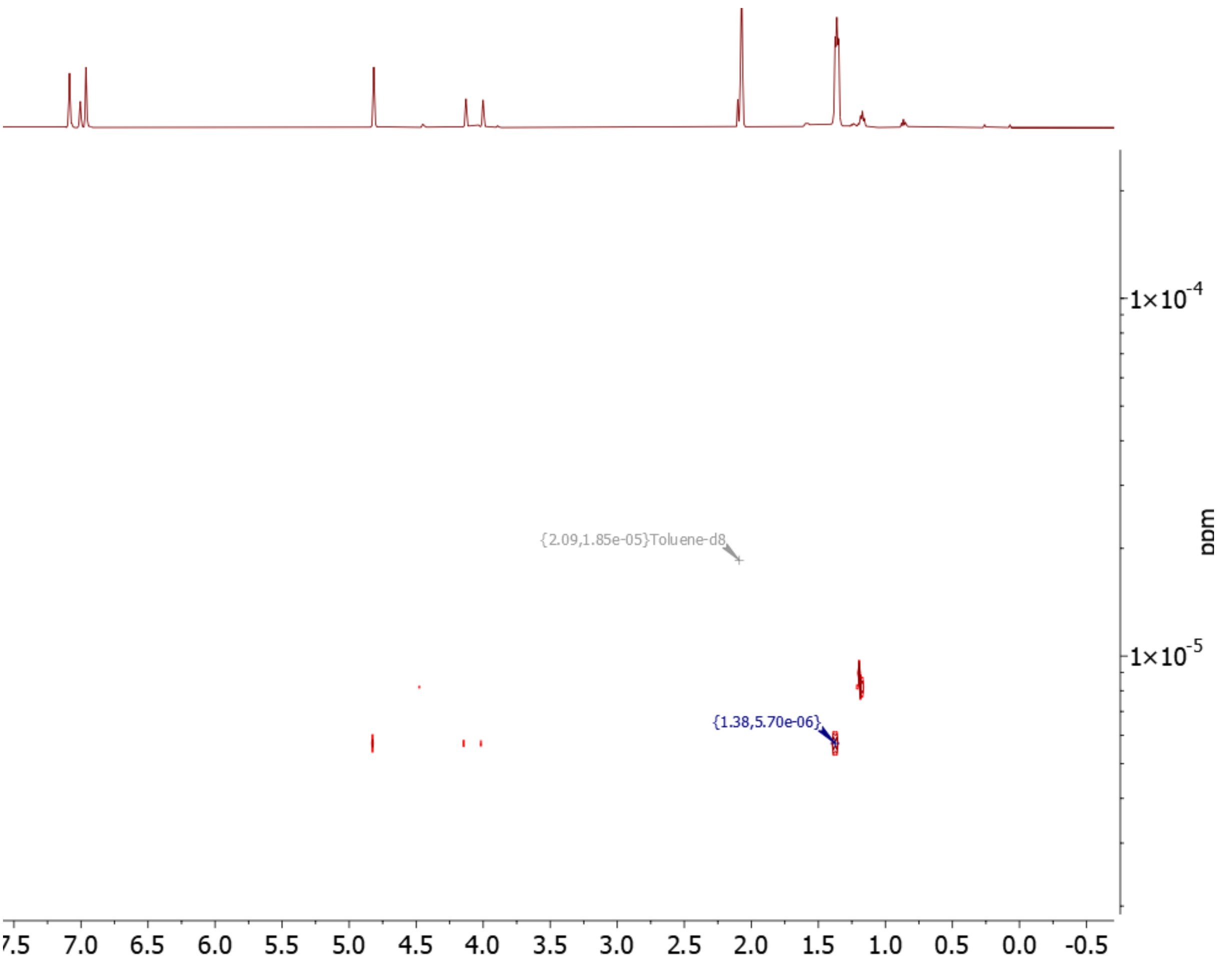

Figure S17: ^{1}H-DOSY-NMR-spectrum of compound **2-Ba** recorded in toluene-d_8 at 126 MHz. The obtained data were evaluated according to Stalke *et al*. and gave an estimated molar weight of 867 g/mol, which is 6% off its actual molar weight, when using the compact sphere model and the toluene solvent signal as reference.[6]

1.2.5. Synthesis of compound **2-Ba(THF)$_2$**

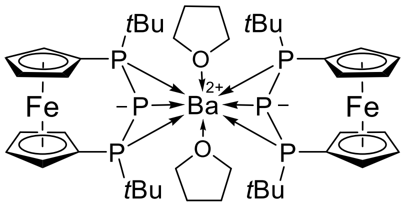

Chemical Formula: $C_{44}H_{68}BaFe_2O_2P_6$
Molecular Weight: 1063.89

2-Ba(THF)$_2$

An NMR tube was charged with 30 mg (0.07 mmol) **1-H**, 18 mg (0.04 mmol, 0.51 eq) Ba[N(SiMe$_3$)$_2$]$_2$ and 1 ml THF. The resulting solution was allowed to react for 16 h. Afterwards, the solution was stored at –36 °C for 16 h, resulting in crystallisation of the product as red needles. Finally, the mother liquor was discarded and the solid dried in vacuum.

Yield: 54 mg ≙ 73 %

NMR-data [ppm]:

^{1}H NMR (500 MHz, toluene-d_8): δ 4.87 (m, 4H, Cp), 4.67 (m, 4H, Cp), 4.16 (m, 4H, Cp), 4.00 (m, 4H, Cp) 3.69 (m, 8H, THF), 1.46-1.40 (m, 44H, *t*Bu+THF).

^{13}C{^{1}H} NMR (126 MHz, toluene-d_8): δ 85.7 (m, *ipso*-Cp), 78.1 (m, Cp), 73.2 (m, Cp), 71.4 (s, Cp), 68.6 (m, THF+Cp), 31.9 (m, *t*Bu C_q), 30.6 (m, *t*Bu), 25.6 (s, THF).

^{31}P{^{1}H} NMR (202 MHz, toluene-d_8): δ 23.8 (d, $^{1}J_{PP}$ = 272 Hz), –125.8 (t, $^{1}J_{PP}$ = 272 Hz).

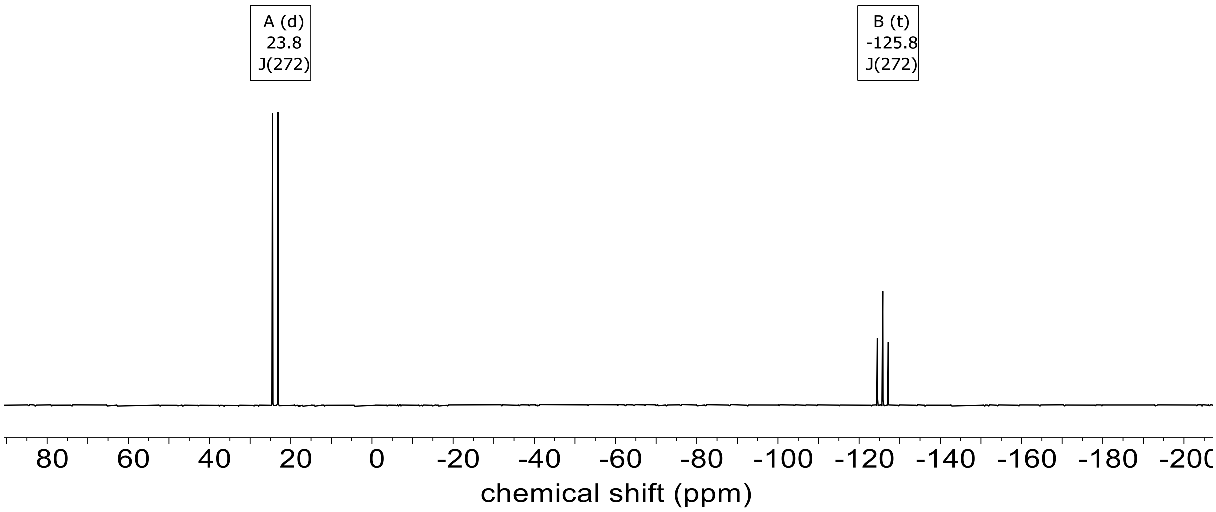

Figure S18: ^{31}P{^{1}H}-NMR-spectrum of compound **2-Ba(THF)₂** recorded in toluene-d_8 at 202 MHz.

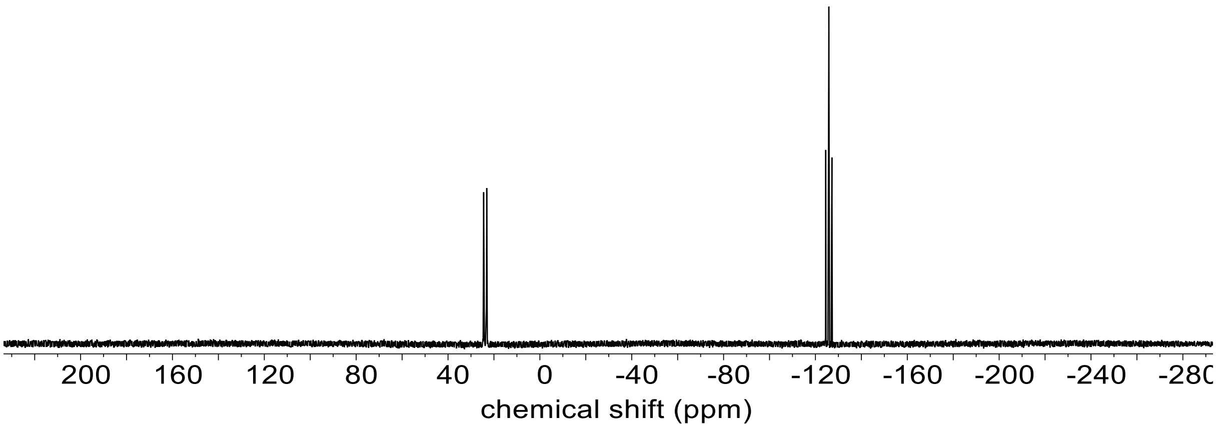

Figure S19: ^{31}P-NMR-spectrum of compound **2-Ba(THF)$_2$** recorded in toluene-d_8 at 202 MHz.

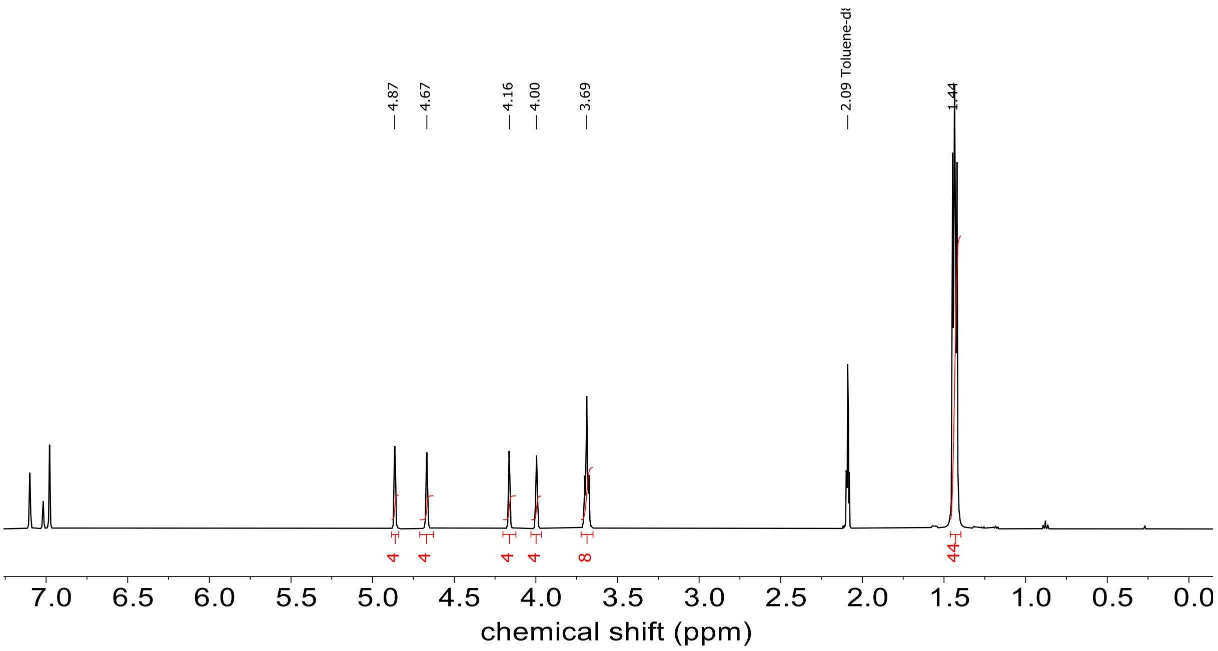

Figure S20: ^{1}H-NMR-spectrum of compound **2-Ba(THF)$_2$** recorded in toluene-d_8 at 500 MHz.

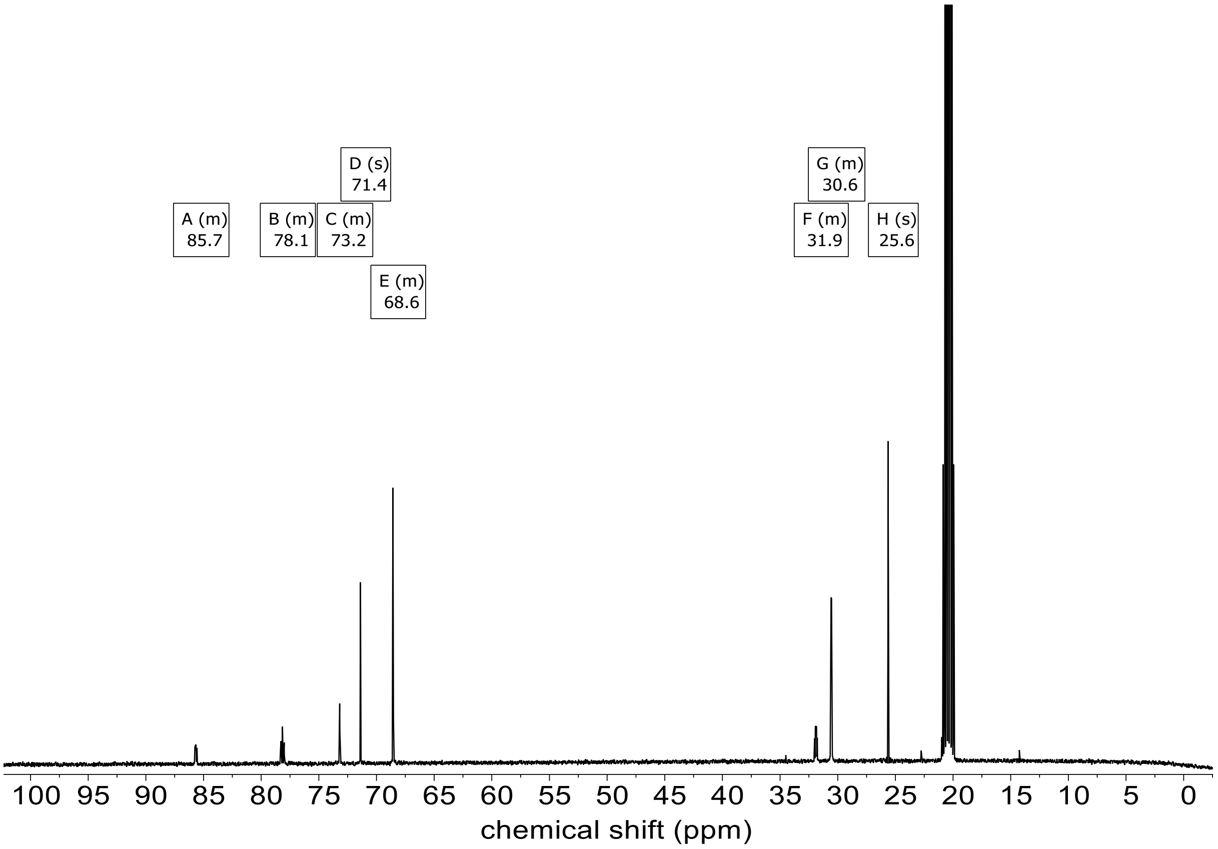

Figure S21: $^{13}C\{^{1}H\}$-NMR-spectrum of compound **2-Ba(THF)$_2$** recorded in toluene-d_8 at 126 MHz. The remaining signals of toluene-d_8 are omitted.

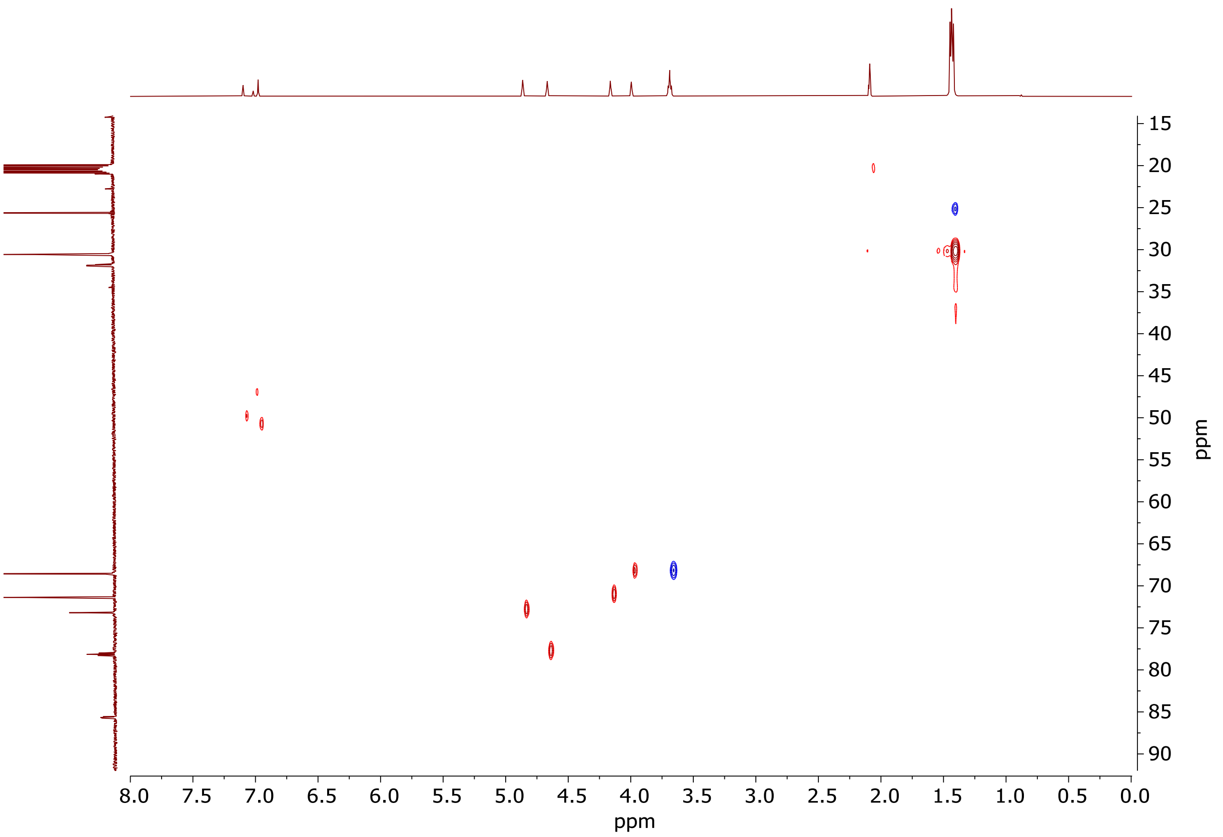

Figure S22: ^{1}H-^{13}C-HSQC NMR-spectrum of compound **2-Ba(THF)₂** recorded in toluene-d_8 at 126 MHz. The remaining signals of toluene-d_8 are omitted.

1.2.6. Synthesis of compound **2-Zn**

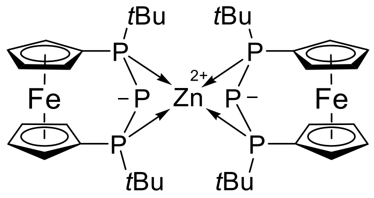

Chemical Formula: $C_{36}H_{52}Fe_2P_6Zn$
Molecular Weight: 847.73

2-Zn

120 mg (0.30 mmol, 2.1 eq.) **1-Li** and 36 mg $ZnCl_2$·TMEDA (0.14 mmol, 0.5 eq.) were suspended in 10 ml toluene and stirred for 16 h at room temperature. Afterwards, the volatile compounds were removed in vacuum and the crude was suspended in 5 ml *n*-pentane. The suspension was centrifugated and the solution removed. Subsequently, the pellet was dried in vacuum. The product was obtained as orange powder. Single crystals were received upon cooling a concentrated solution in toluene to –30 °C.

Yield: 70 mg ≙ 58%

NMR-data [ppm]:

^{1}H NMR (500 MHz, C_6D_6): δ 4.97 (m, Cp, 4H), 4.88 (m, Cp, 4H), 4.20 (m, Cp, 4H), 1.50 (m, *t*Bu, 36 H).

$^{13}C\{^1H\}$ NMR (126 MHz, C_6D_6): δ 85.6 (s, *ipso*-Cp), 77.1 (m, *ipso*-Cp), 74.0 (d, $^2J_{CP}$ = 2.4 Hz, Cp), 73.0 (s, Cp), 69.4 (s, Cp), 34.5 (m, *t*Bu, Cp C_q), 30.2 (m, *t*Bu).

$^{31}P\{^1H\}$ NMR (202 MHz, C_6D_6): δ 38.9 (bd, $^1J_{PP}$ = 322 Hz), –114.9 (t, $^1J_{PP}$ = 322 Hz).

Elemental analysis [%]: Calculated: C 51.01, H 6.18; found: C 51.36, H 6.24.

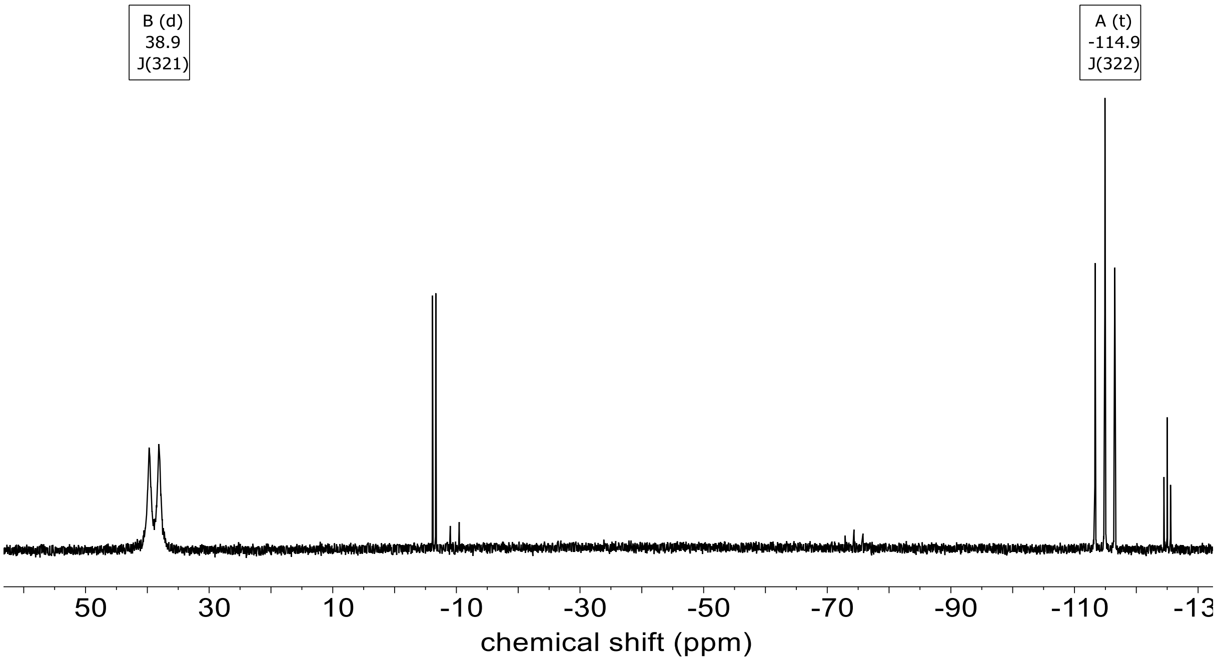

Figure S23: $^{31}P\{^1H\}$-NMR-spectrum of compound **2-Zn** recorded in C_6D_6 at 202 MHz.

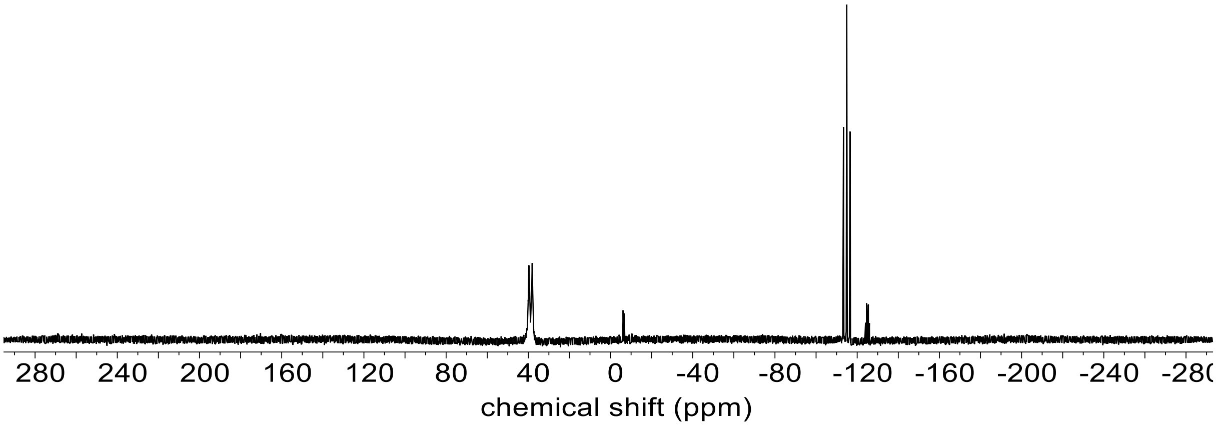

Figure S24: ^{31}P-NMR-spectrum of compound **2-Zn** recorded in C_6D_6 at 202 MHz.

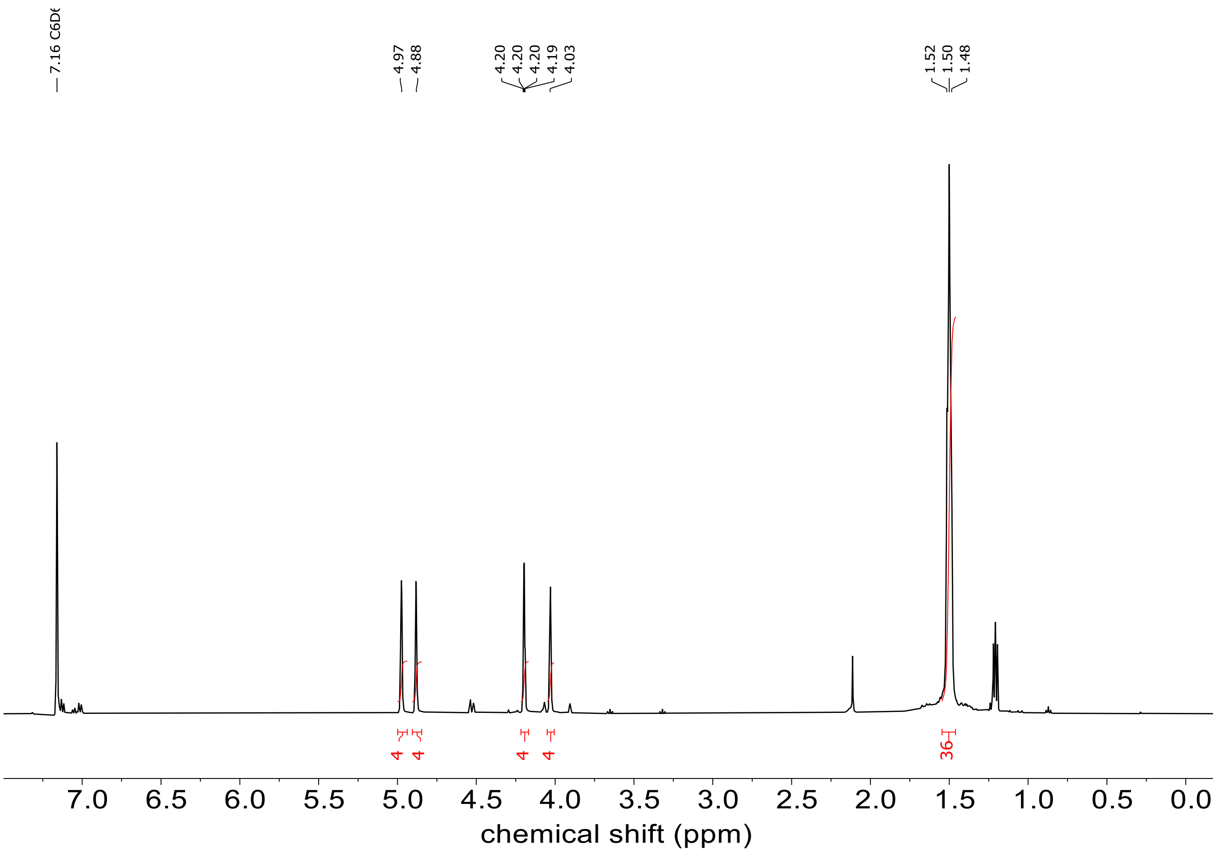

Figure S25: ^{1}H-NMR-spectrum of compound **2-Zn** recorded in C_6D_6 at 500 MHz.

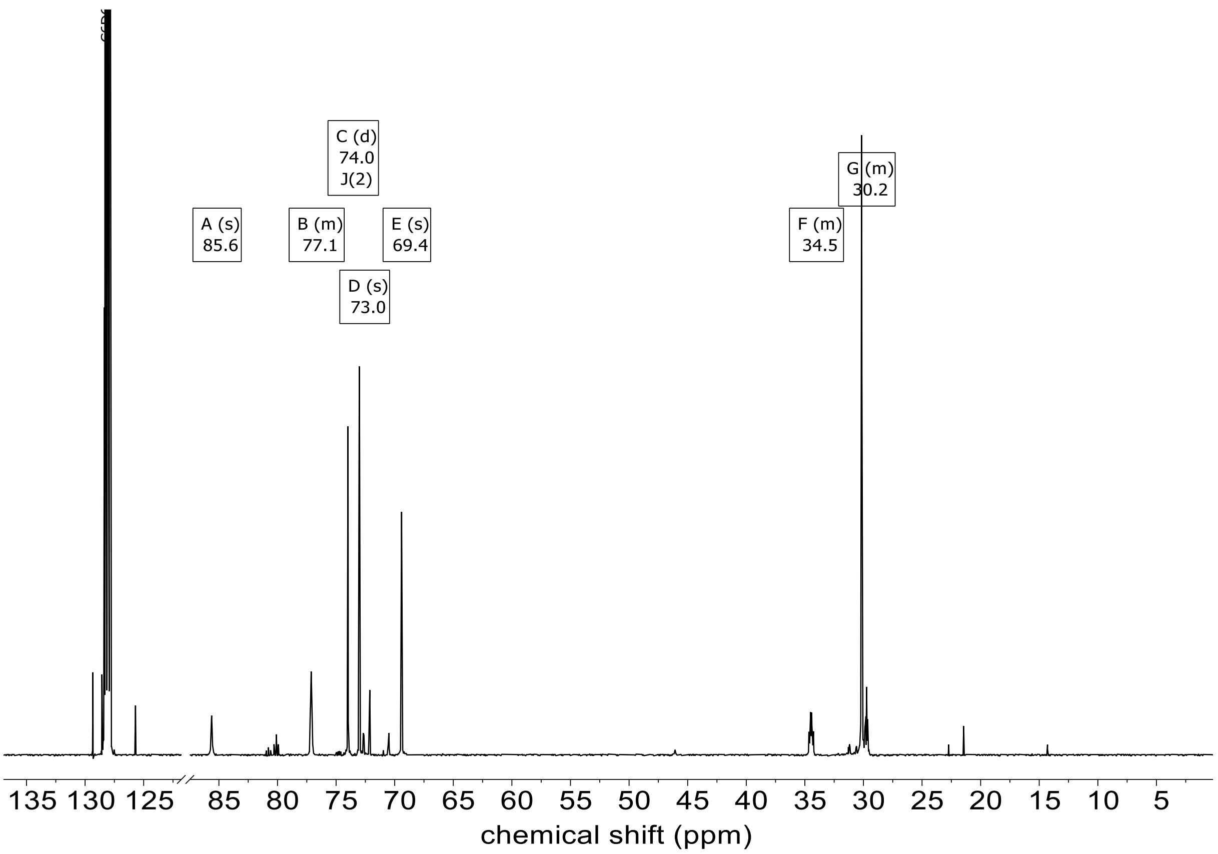

Figure S26: $^{13}C\{^{1}H\}$-NMR-spectrum of compound **2-Zn** recorded in C_6D_6 at 126 MHz.

1.2.7. Synthesis of compound **3-Cu(IMe)**

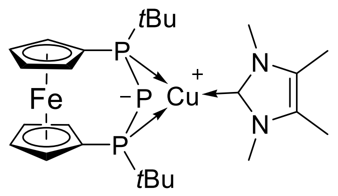

Chemical Formula: $C_{25}H_{38}CuFeN_2P_3$
Molecular Weight: 578.90

3-Cu(IMe)

40 mg (0.10 mmol) **1-Li** and 32 mg (0.10 mmol) $Cu(MeCN)_4BF_4$ were mixed in a Schlenk flask with 5 ml THF. The resulting suspension was treated with ultrasonics for 2 h and then stirred for 16 h at room temperature. Afterwards, volatile compounds were removed in vacuum. The crude was suspended in 7 ml toluene, 13 mg (0.10 mmol) IMe were added, and the resulting suspension was stirred for 16 h at room temperature. Afterwards, the reaction mixture was filtered, and volatile compounds of the filtrate were removed in vacuum. The product was obtained as brown powder. Single crystals were obtained by slow evaporation of a concentrated toluene solution.

Yield: 34 mg ≙ 59%

NMR-data [ppm]:

^{1}H NMR (400 MHz, C_6D_6): δ 5.19 (m, 2H, Cp), 4.73 (m, 2H, Cp), 4.34 (m, 2H, Cp), 4.13 (m, 2H, Cp), 3.34 (s, 6H, NMe), 1.56 – 1.49 (m, 18H, *t*Bu), 1.35 (s, 6H, C=CMe).

$^{13}C\{^{1}H\}$ NMR (101 MHz, C_6D_6): δ 184.3 (m, N*C*N), 123.8 (s, C=C), 89.3 (m, *ipso*-Cp), 77.7 (m, Cp), 73.8 (s, Cp), 71.8 (s, Cp), 68.7 (pt, $^{2}J_{CP}$ = 3 Hz , Cp), 35.3 (s, N*C*H₃), 31.7 (m, *t*Bu), 30.1 (q, $^{2}J_{CP}$ = 4 Hz, *t*Bu C_q), 8.4 (s, C=C*C*H₃).

$^{31}P\{^{1}H\}$ NMR (202 MHz, C_6D_6): δ 25.1 (d, $^{1}J_{PP}$ = 270 Hz, *t*Bu*P*), –132.5 (bt, $^{1}J_{PP}$ = 270 Hz P*P*P).

Elemental analysis [%]: Calculated: C 51.78, H 6.78, N 4.83; found: C 51.87, H 6.97 N 4.66.

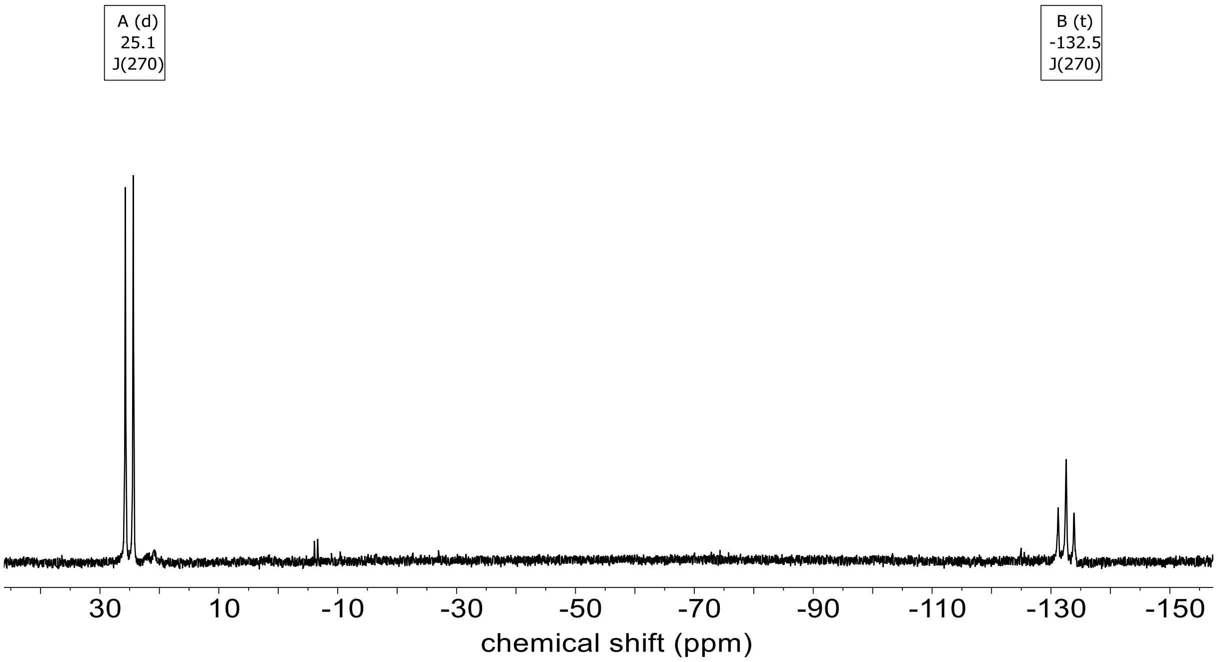

Figure S27: $^{31}P\{^{1}H\}$-NMR-spectrum of compound **3-Cu(IMe)** recorded in C_6D_6 at 202 MHz.

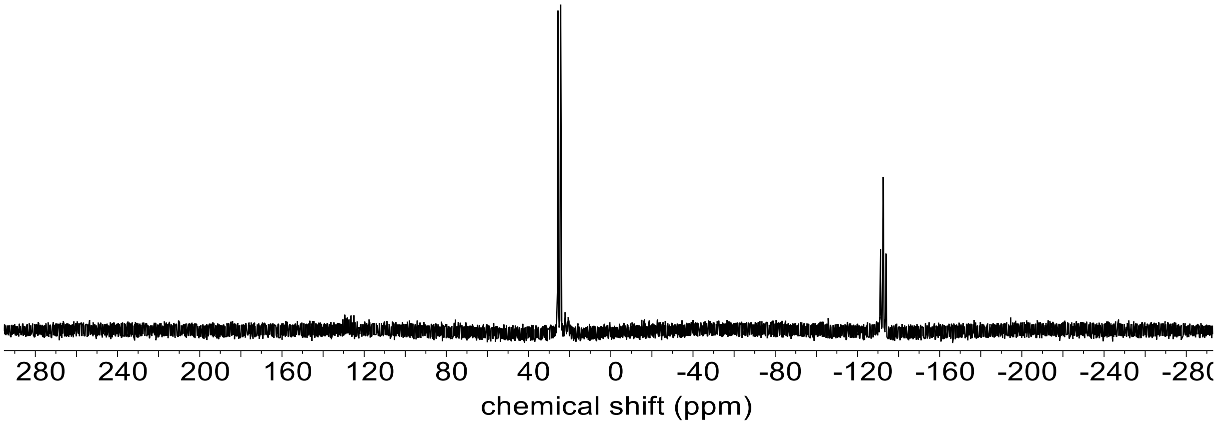

Figure S28: ^{31}P-NMR-spectrum of compound **3-Cu(IMe)** recorded in C_6D_6 at 202 MHz.

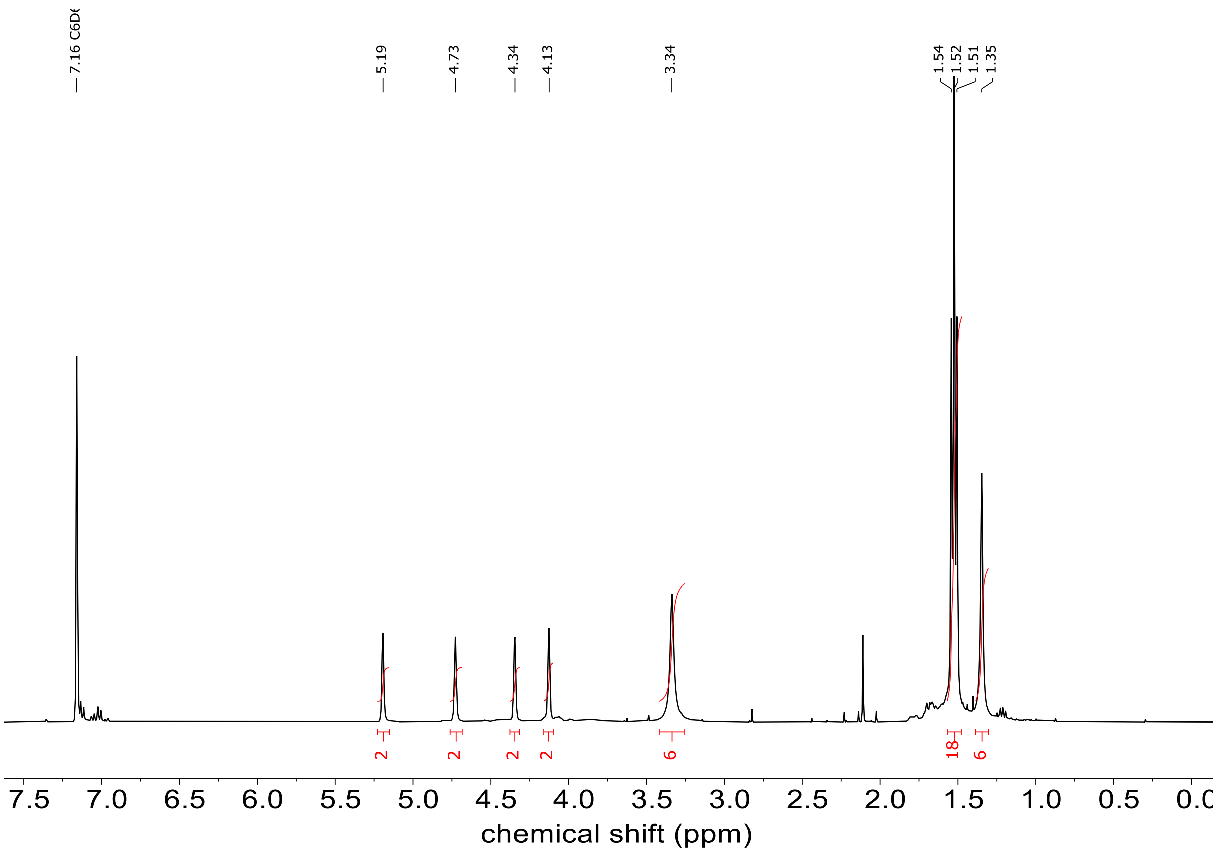

Figure S29: ^{1}H-NMR-spectrum of compound **3-Cu(IMe)** recorded in C_6D_6 at 202 MHz.

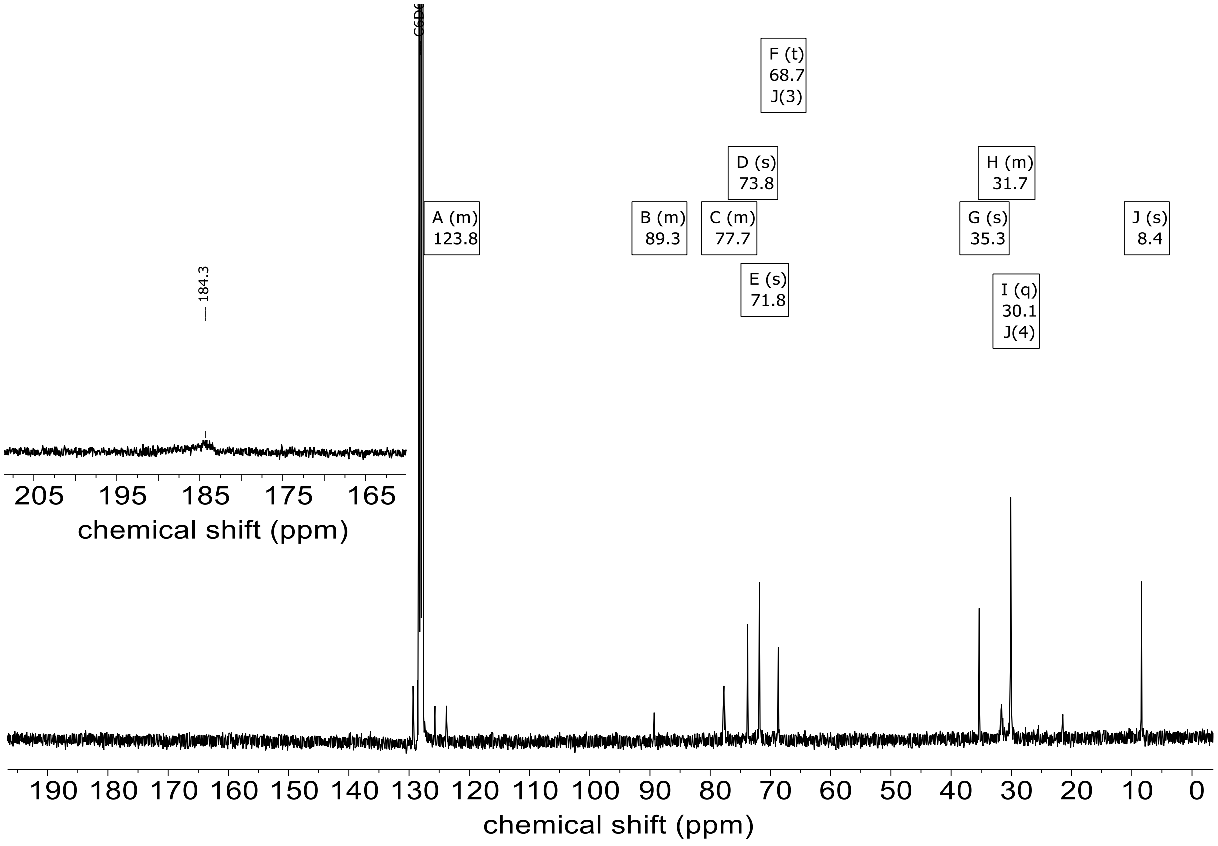

Figure S30: $^{13}C\{^{1}H\}$-NMR-spectrum of compound **3-Cu(IMe)** recorded in C_6D_6 at 126 MHz.

1.2.8. Synthesis of compound **3-Ag(IMe)**

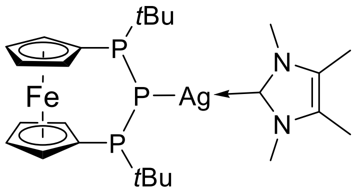

Chemical Formula:
$C_{25}H_{38}AgFeN_2P_3$
Molecular Weight: 623.23
3-Ag·IMe

103 mg (0.26 mmol) **1-Li** and 46 mg (0.24 mmol, 0.91 eq.) $AgBF_4$ were mixed in a Schlenk flask with 10 ml THF. The reaction mixture was stirred for 1.5 h at room temperature and volatile compounds were completely removed in vacuum afterwards. The crude was washed with 10 ml *n*-pentane once and dried in vacuum again. Subsequently, 10 ml toluene were added, and the resulting suspension was filtered after treatment with ultrasonics. 19 mg (0.15 mmol, 0.59 eq) of IMe were added to the solution which was then stirred for two days at room temperature and filtered. Afterwards, volatile compounds of the filtrate were removed in vacuum. The product was obtained as light brown powder. Single crystals were obtained by slow diffusion of *n*-pentane in a saturated toluene solution.

Yield: 55 mg ≙ 34%

NMR-data [ppm]:

^{1}H NMR (500 MHz, C_6D_6): δ 5.12 (m, 2H, Cp), 4.81 (m, 2H, Cp), 4.30 (m, 2H, Cp), 4.07 (m, 2H, Cp), 2.97 (s, 6H, NMe), 1.73-1.64 (m, 18H, *t*Bu), 1.19 (s, 6H, C=CMe).

^{13}C{^{1}H} NMR (126 MHz, C_6D_6): δ 185.6 – 184.2 (m, NCN), 124.7 – 124.4 (m, C=C), 87.7 (m, *ipso*-Cp), 79.1 (pt, $^2J_{CP}$ = 20 Hz, Cp), 72.1 (m, Cp), 71.1 (m, Cp), 68.7 (m, Cp), 35.4 (s, *t*Bu C_q), 31.6 (pq, $^2J_{CP}$ = 6.5 Hz, *t*Bu), 30.3 (m, N*C*H_3), 8.5 (s, C=C*C*H_3).

^{31}P{^{1}H} NMR (202 MHz, C_6D_6): δ 12.1 (bd, $^1J_{PP}$ = 165 Hz, *t*Bu*P*), –118.7 (m, P*P*P).

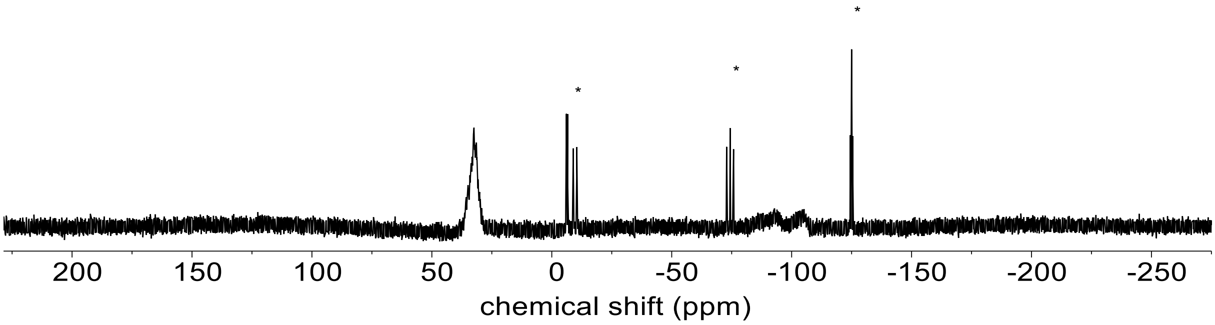

Figure S31: ^{31}P{^{1}H}-NMR-spectrum of the poly-/oligomeric compounds **3-Ag** recorded in non-deuterated solvent at 202 MHz. Asterisks (*) mark the resonances corresponding to the product of hydrolysis, **1-H**.

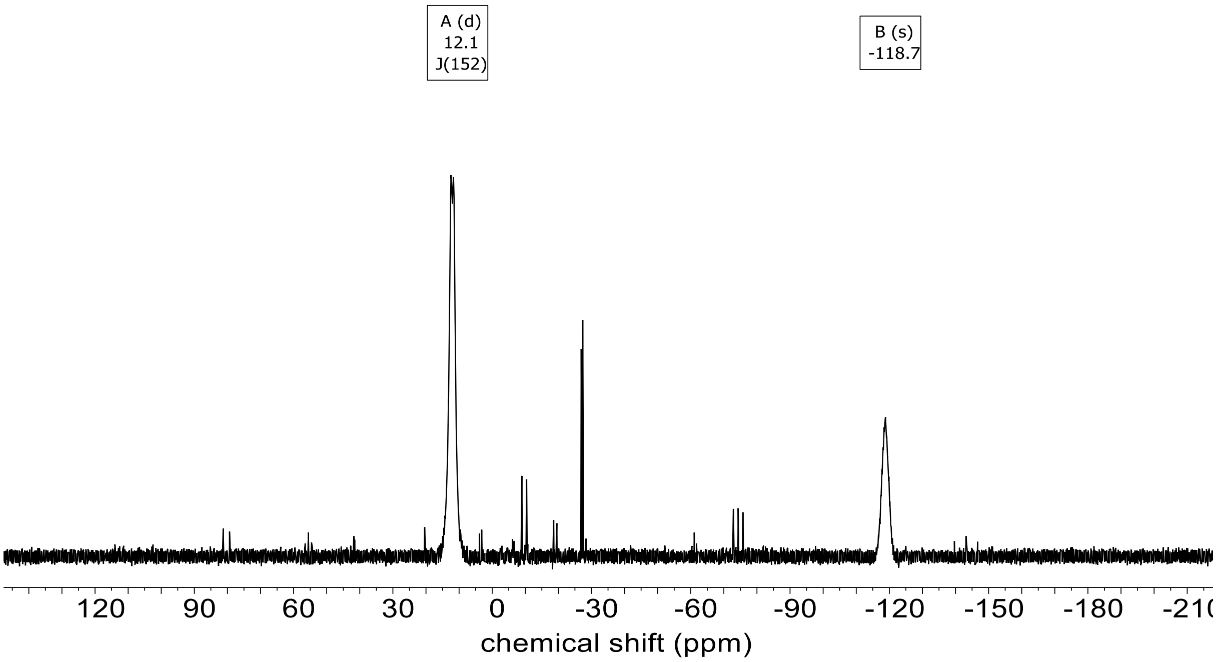

Figure S32: $^{31}P\{^{1}H\}$-NMR-spectrum of compound **3-Ag(IMe)** recorded in C_6D_6 at 202 MHz.

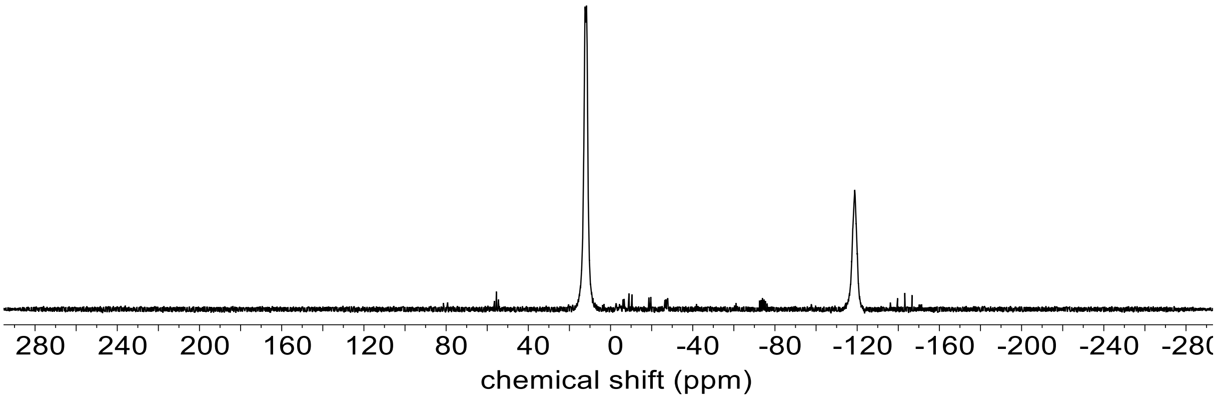

Figure S33: ^{31}P-NMR-spectrum of compound **3-Ag(IMe)** recorded in C_6D_6 at 202 MHz.

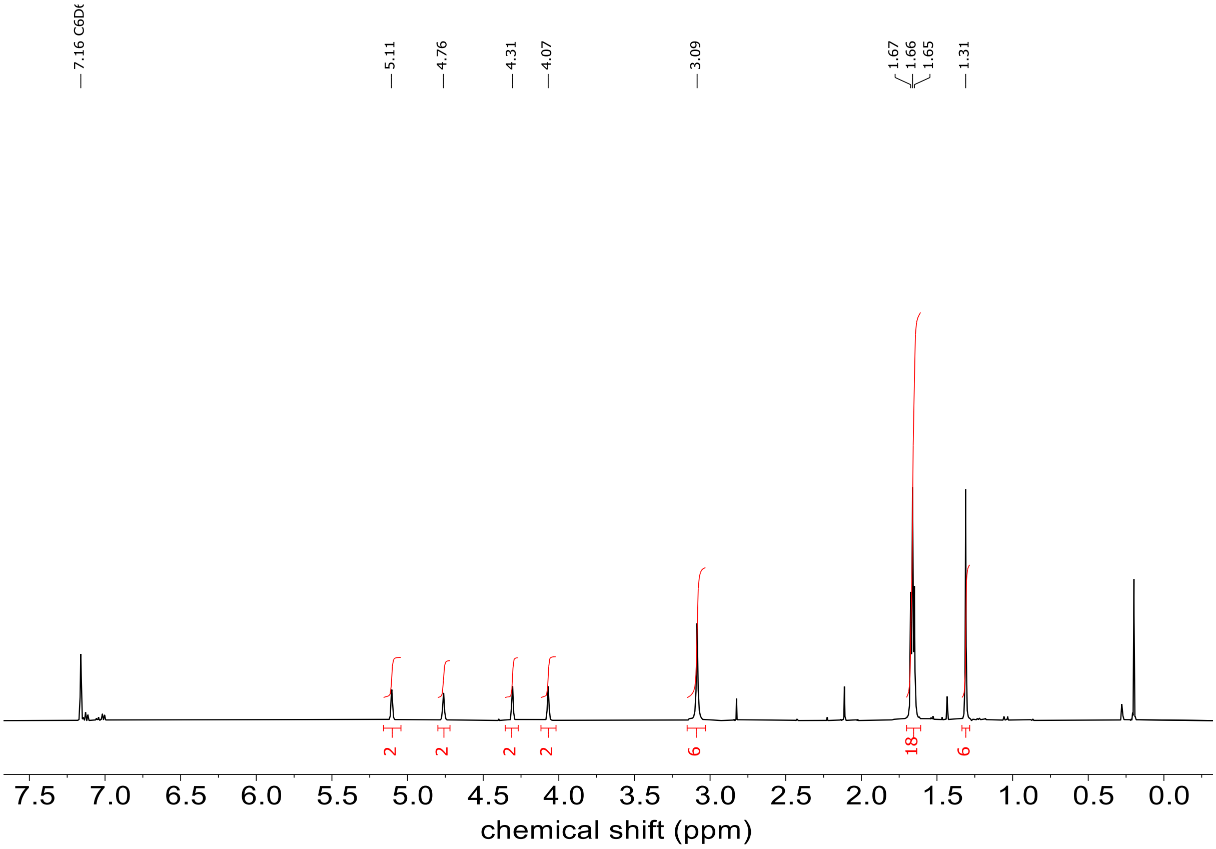

Figure S34: ^{1}H-NMR-spectrum of compound **3-Ag(IMe)** recorded in C_6D_6 at 202 MHz.

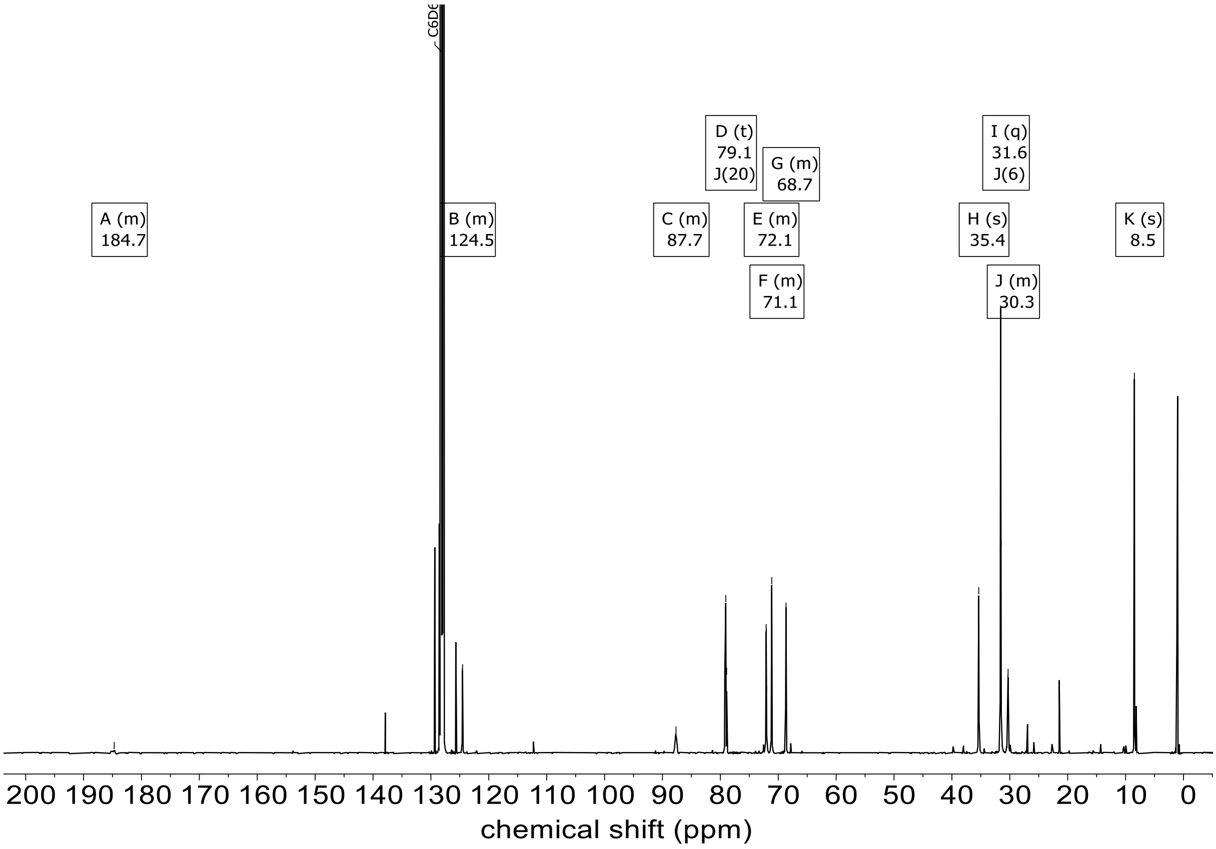

Figure S35: $^{13}C\{^{1}H\}$-NMR-spectrum of compound **3-Ag(IMe)** recorded in C_6D_6 at 126 MHz.

1.2.9. Synthesis of compound **3-Au(IMe)**

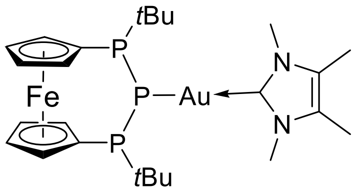

Chemical Formula: $C_{25}H_{38}AuFeN_2P_3$
Molecular Weight: 712.33
3-Au(IMe)

40 mg (0.10 mmol) **1-Li** and 32 mg (0.10 mmol) AuCl·THT were mixed in a Schlenk flask with 5 ml Et_2O. The reaction mixture was stirred for 1 h at room temperature and afterwards, volatile compounds were removed in vacuum. Subsequently, 5 ml toluene and 9 mg (0.07 mmol, 0.7 eq) of IMe were added to the crude which was then stirred for 16 h at room temperature and filtered. Afterwards, volatile compounds of the filtrate were removed in vacuum. The product was obtained as yellow powder. Single crystals were obtained by slow evaporation of a concentrated toluene solution.

Yield: 30 mg ≙ 42%

NMR-data [ppm]:

^{1}H NMR (500 MHz, C_6D_6): δ 5.09 (m, 2H, Cp), 4.74 (m, 2H, Cp), 4.29 (m, 2H, Cp), 4.07 (m, 2H, Cp), 2.95 (s, 6H, NMe), 1.77 – 1.72 (m, 18H, *t*Bu), 1.12 (s, 6H, C=CMe).

^{13}C{^{1}H} NMR (126 MHz, C_6D_6): δ 193.9 (d, $^1J_{CP}$ = 40 Hz, N*C*N), 124.2 (s, C=C), 85.1 (m, *ipso*-Cp), 79.3 (m, Cp), 72.1 (m, Cp), 71.4 (m, Cp), 69.2 (m, Cp), 34.1 (s, N*C*H_3), 31.6 (m, *t*Bu), 30.0 (s, *t*Bu C_q), 8.3 (s, C=C*C*H_3).

^{31}P{^{1}H} NMR (202 MHz, C_6D_6): δ 9.0 (d, $^1J_{PP}$ = 145 Hz, *t*Bu*P*), –118.7 (bt, $^1J_{PP}$ = 145 Hz P*P*P).

Elemental analysis [%]: Calculated: C 42.09, H 5.51, N 3.93; found: C 42.48, H 5.39, N 4.02.

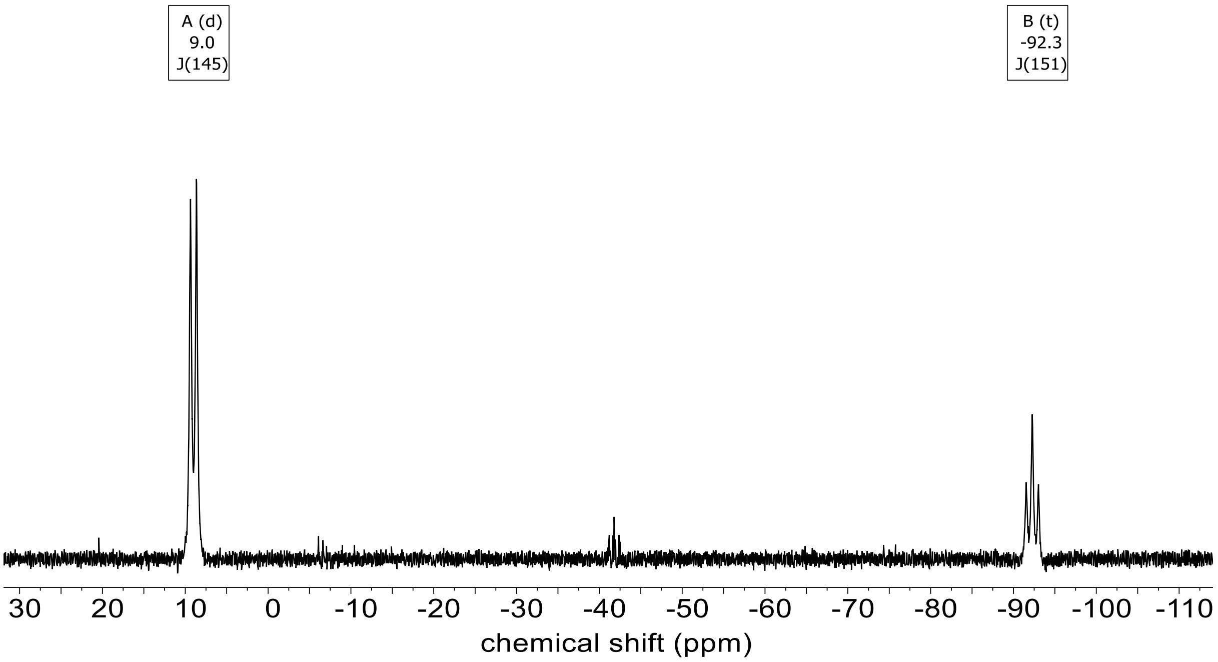

Figure S36: $^{31}P\{^{1}H\}$-NMR-spectrum of compound **3-Au(IMe)** recorded in C_6D_6 at 202 MHz.

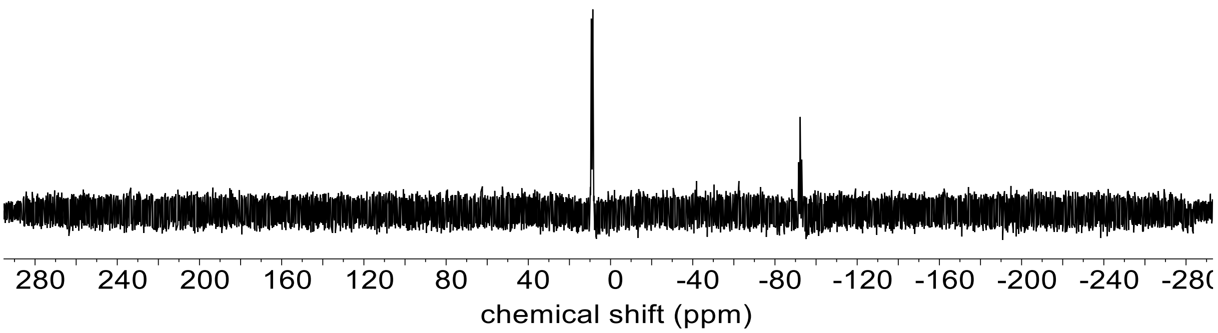

Figure S37: ^{31}P-NMR-spectrum of compound **3-Au(IMe)** recorded in C_6D_6 at 202 MHz.

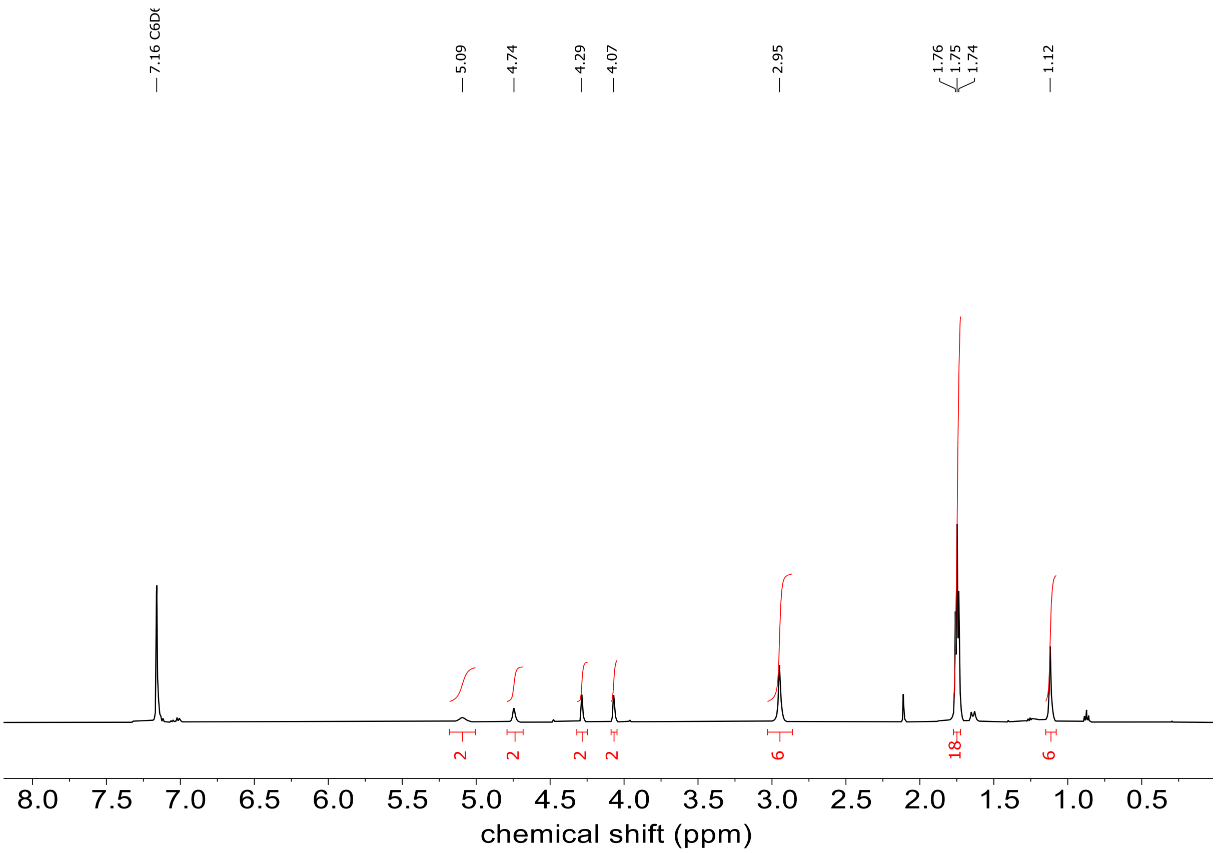

Figure S38: ^{1}H-NMR-spectrum of compound **3-Au(IMe)** recorded in C_6D_6 at 202 MHz.

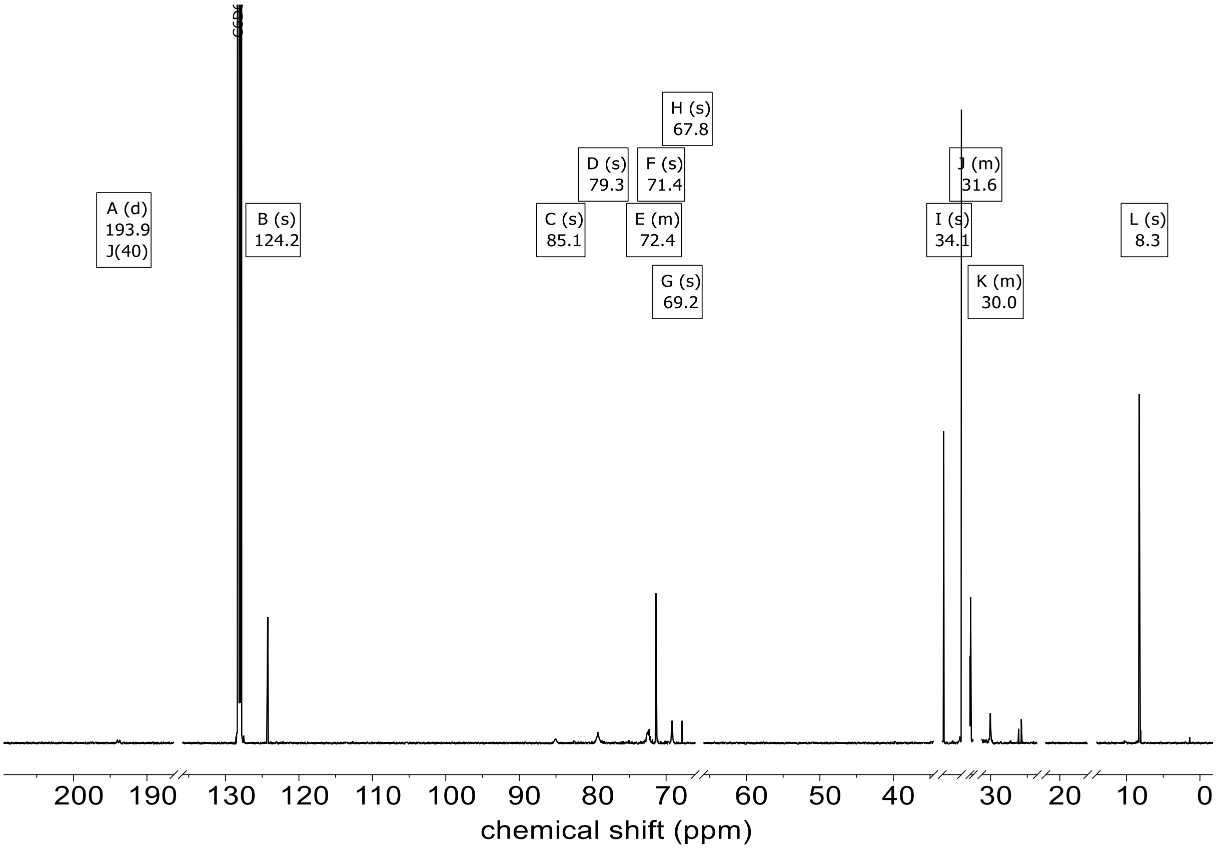

Figure S39: ^{13}C{^{1}H}-NMR-spectrum of compound **3-Au(IMe)** recorded in C_6D_6 at 126 MHz.

2. Crystal data and refinement details

Crystallographic measurements were carried out on a *Stoe* IPDS2 or a *Stoe* StadiVari diffractometer with a STOE image plate detector and a Mo-Kα (λ = 0.71073 Å) monochromator or a *Stoe* StadiVari diffractometer with a Pilatus 200K image plate detector and Cu-Kα (λ = 1.54186 Å) radiation. Direct methods were used to solve the measurements and refined by "least-square" cycles (SHELXL-2017).[7] All non-hydrogen atoms were anisotropically refined without restriction. The evaluation of the data sets, as well as the graphical preparation of the structures was carried out using Olex2[8] and Mercury.[9] Details of the structure determinations and refinement are summarized in Table S1, Table S2, Table S3 and Table S4. Crystallographic data for the molecular structures reported in this paper have been deposited at the CCDC under the deposition numbers 2420580-2420590 and 2426776 and can be obtained from https://www.ccdc.cam.ac.uk/structures.

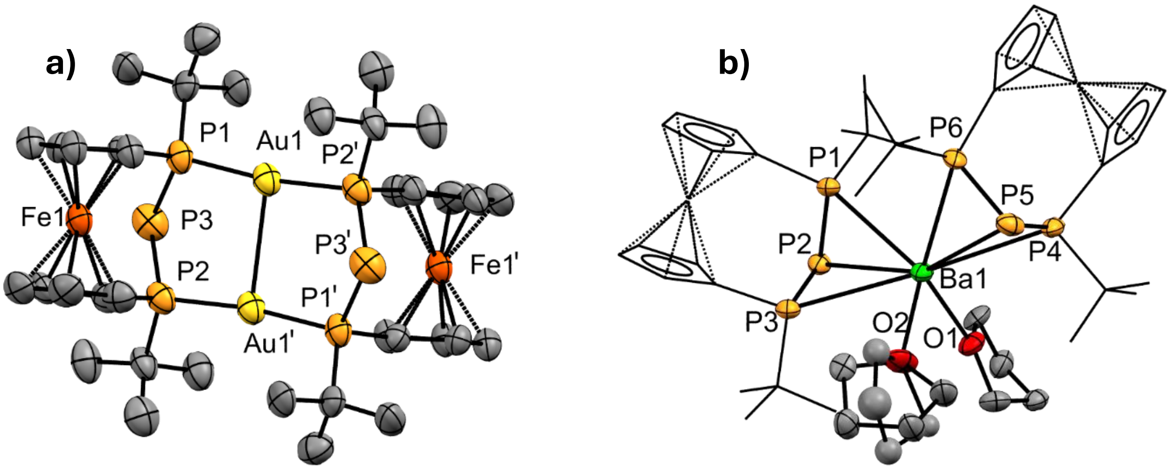

Figure S40: ORTEP-depiction of a) dimeric **(3-Au)₂** and b) **2-Ba(THF)₂**. Hydrogen atoms are omitted for clarity. The thermal ellipsoids are represented at a 30% probability level. For **2-Ba(THF)₂**, a second molecule with similar binding parameters as well as a non-coordinating THF lattice molecule were omitted. Selected bond lengths [Å] and angles [°]: a) P1–Au1 2.298(7), P2'–Au1 2.294(7), Au1–Au1' 2.955(2), P1–P3 2.220(10), P2–P3 2.196(12), P1–P3–P2 90.4(5), P1–Au1–P2' 172.7(3). b) P1–Ba1 3.206(5), P2–Ba1 3.473(5), P3–Ba1 3.310(4), P4–Ba1 3.288 (4), P5–Ba1 3.507(4), P6–Ba1 3.253(4), P1–P2 2.167(6), P1–P2–P3 88.4(3).

Table S1: Crystal data and structure refinement for compounds **2-Mg(THF)**, **2-Mg** and **2-Ca(THF)**.

**Compound**	**2-Mg(THF)**	**2-Mg**	**2-Ca(THF)**
CCDC code	2420580	2420581	2420582
Identification code	i3583	i3686	sv1576
Empirical formula	C44H68Fe2MgO2P6	$C_{38.5}H_{57}Fe_2MgP_6$	$C_{40}H_{60}CaFe_2OP_6$
Formula weight	950.81	841.67	894.48
Temperature/K	100	100	100
Crystal system	monoclinic	monoclinic	monoclinic
Space group	$P2_1/n$	C2/c	$P2_1$

a/Å	10.7693(7)	38.844(3)	10.7115(3)
b/Å	22.3563(19)	16.1269(6)	36.1279(9)
c/Å	19.3369(12)	13.7747(9)	11.8135(3)
α/°	90	90	90
β/°	96.403(5)	91.346(6)	106.719(2)
γ/°	90	90	90
Volume/Å^3	4626.5(6)	8626.6(9)	4378.4(2)
Z	4	8	4
ρ_{calc} g/cm^3	1.365	1.296	1.357
μ/mm^{-1}	0.883	0.935	1.029
F(000)	2008.0	3536.0	1880.0
Crystal size/mm^3	0.19 × 0.157 × 0.12	0.32 × 0.213 × 0.15	0.21 × 0.133 × 0.05
Radiation	Mo Kα (λ = 0.71073)	Mo Kα (λ = 0.71073)	Mo Kα (λ = 0.71073)
2Θ range for data collection/°	2.794 to 54.07	2.098 to 53.976	4.248 to 63.274
Index ranges	-13 ≤ h ≤ 13, -28 ≤ k ≤ 28, -24 ≤ l ≤ 24	-48 ≤ h ≤ 49, -20 ≤ k ≤ 20, -15 ≤ l ≤ 17	-15 ≤ h ≤ 15, -49 ≤ k ≤ 52, -12 ≤ l ≤ 17
Reflections collected	32027	21866	75429
Independent reflections	10001 [R_{int} = 0.0771, R_{sigma} = 0.0696]	9272 [R_{int} = 0.0557, R_{sigma} = 0.0550]	25679 [R_{int} = 0.0265, R_{sigma} = 0.0429]
Data/restraints/parameters	10001/0/508	9272/0/445	25679/1/925
Goodness-of-fit on F^2	1.051	1.108	1.026
Final R indexes [I>=2σ (I)]	R_1 = 0.0729, wR_2 = 0.1916	R_1 = 0.0510, wR_2 = 0.1412	R_1 = 0.0349, wR_2 = 0.0712
Final R indexes [all data]	R_1 = 0.1149, wR_2 = 0.2281	R_1 = 0.0669, wR_2 = 0.1624	R_1 = 0.0480, wR_2 = 0.0752
Largest diff. peak/hole / e Å^{-3}	1.06/-0.96	0.84/-0.54	0.61/-0.27

Table S2: Crystal data and structure refinement for compounds **2-Sr**, **2-Ba** and **2-Zn**.

**Compound**	**2-Sr**	**2-Ba**	**2-Zn**
CCDC code	2420583	2420585	2420586
Identification code	i3587	i3649	i3566
Empirical formula	$C_{36}H_{52}Fe_2P_6Sr$	$C_{72}H_{104}Ba_2Fe_4P_{12}$	$C_{36}H_{52}Fe_2P_6Zn$
Formula weight	869.91	1839.27	847.66
Temperature/K	100	100	100
Crystal system	monoclinic	monoclinic	monoclinic
Space group	$P2_1/n$	$P2_1/n$	C2/c
a/Å	13.1509(7)	13.2501(7)	23.8382(15)
b/Å	18.4145(13)	18.3514(14)	15.1802(6)
c/Å	17.1570(9)	17.5118(11)	12.3582(6)
α/°	90	90	90
β/°	97.034(4)	97.932(5)	107.490(5)

γ/°	90	90	90
Volume/Å^3	4123.6(4)	4217.4(5)	4265.3(4)
Z	4	2	4
ρ_{calc} g/cm^3	1.401	1.448	1.320
μ/mm^{-1}	2.239	1.853	1.477
F(000)	1792.0	1864.0	1760.0
Crystal size/mm^3	0.29 × 0.193 × 0.12	0.22 × 0.127 × 0.08	0.33 × 0.23 × 0.1
Radiation	Mo Kα (λ = 0.71073)	Mo Kα (λ = 0.71073)	Mo Kα (λ = 0.71073)
2Θ range for data collection/°	3.258 to 53.996	3.23 to 54.046	3.226 to 54.066
Index ranges	-14 ≤ h ≤ 16, -23 ≤ k ≤ 23, -21 ≤ l ≤ 21	-16 ≤ h ≤ 13, -23 ≤ k ≤ 23, -22 ≤ l ≤ 22	-30 ≤ h ≤ 27, -19 ≤ k ≤ 18, -15 ≤ l ≤ 15
Reflections collected	25883	27876	14764
Independent reflections	8867 [R_{int} = 0.0586, R_{sigma} = 0.0499]	9069 [R_{int} = 0.0414, R_{sigma} = 0.0411]	4586 [R_{int} = 0.0610, R_{sigma} = 0.0530]
Data/restraints/parameters	8867/0/418	9069/0/418	4586/0/210
Goodness-of-fit on F^2	1.088	1.069	1.054
Final R indexes [I>=2σ (I)]	R_1 = 0.0647, wR_2 = 0.1628	R_1 = 0.0403, wR_2 = 0.0996	R_1 = 0.0534, wR_2 = 0.1419
Final R indexes [all data]	R_1 = 0.0898, wR_2 = 0.1842	R_1 = 0.0546, wR_2 = 0.1062	R_1 = 0.0679, wR_2 = 0.1485
Largest diff. peak/hole / e Å^{-3}	1.62/-1.37	1.59/-1.21	0.67/-1.16

Table S3: Crystal data and structure refinement for compounds **3-Cu(IMe)**, **3-Ag(IMe)** and **3-Au(IMe)**.

Compound	3-Cu(IMe)	3-Ag(IMe)	3-Au(IMe)
CCDC code	2420587	2420588	2420589
Identification code	i3543	i3688	i3697
Empirical formula	$C_{25}H_{38}CuFeN_2P_3$	$C_{25}H_{38}AgFeN_2P_3$	$C_{25}H_{38}AuFeN_2P_3$
Formula weight	578.87	623.20	712.30
Temperature/K	100	100	100
Crystal system	monoclinic	orthorhombic	orthorhombic
Space group	P2$_1$/n	Pbcn	Pbcn
a/Å	12.660(2)	23.8327(16)	23.8077(10)
b/Å	14.4497(11)	29.2610(18)	28.9777(12)
c/Å	14.9635(18)	10.0754(5)	10.0083(3)
α/°	90	90	90
β/°	90.508(12)	90	90
γ/°	90	90	90
Volume/Å^3	2737.1(6)	7026.3(7)	6904.6(5)
Z	4	8	8
ρ_{calc} g/cm^3	1.405	1.178	1.370

μ/mm^{-1}	1.498	1.119	4.817
F(000)	1208.0	2560.0	2816.0
Crystal size/mm^3	0.24 × 0.167 × 0.08	0.14 × 0.103 × 0.07	0.11 × 0.077 × 0.04
Radiation	Mo Kα (λ = 0.71073)	Mo Kα (λ = 0.71073)	Mo Kα (λ = 0.71073)
2Θ range for data collection/°	3.918 to 54.182	2.204 to 53.936	2.214 to 54.008
Index ranges	-16 ≤ h ≤ 16, -18 ≤ k ≤ 17, -18 ≤ l ≤ 19	-27 ≤ h ≤ 30, -32 ≤ k ≤ 37, -11 ≤ l ≤ 12	-30 ≤ h ≤ 26, -36 ≤ k ≤ 31, -12 ≤ l ≤ 11
Reflections collected	18477	19952	19223
Independent reflections	5912 [R_{int} = 0.0447, R_{sigma} = 0.0438]	7528 [R_{int} = 0.0499, R_{sigma} = 0.0482]	7390 [R_{int} = 0.0777, R_{sigma} = 0.0861]
Data/restraints/parameters	5912/0/299	7528/0/299	7390/0/299
Goodness-of-fit on F^2	1.077	1.060	0.999
Final R indexes [I>=2σ (I)]	R_1 = 0.0497, wR_2 = 0.1365	R_1 = 0.0630, wR_2 = 0.1588	R_1 = 0.0689, wR_2 = 0.1701
Final R indexes [all data]	R_1 = 0.0694, wR_2 = 0.1495	R_1 = 0.0813, wR_2 = 0.1694	R_1 = 0.1193, wR_2 = 0.1983
Largest diff. peak/hole / e Å^{-3}	1.73/-1.04	1.05/-0.99	1.81/-2.38

Table S4: Crystal data and structure refinement for compounds **2-Sr(THF)**, dimeric **(3-Au)$_2$** and **2-Ba(THF)$_2$**.

Compound	2-Sr(THF)	(3-Au)$_2$	2-Ba(THF)$_2$
CCDC code	2420584	2420590	2426776
Identification code	i3701a	i3696a	i3707
Empirical formula	$C_{44}H_{68}Fe_2O_2P_6Sr$	$C_{36}H_{52}Au_2Fe_2P_6$	$C_{92}H_{144}Ba_2Fe_4O_5P_{12}$
Formula weight	1014.12	1176.23	2199.78
Temperature/K	100	100	100
Crystal system	tetragonal	triclinic	triclinic
Space group	P4$_1$	P-1	P-1
a/Å	11.4732(6)	9.5990(18)	11.1773(10)
b/Å	11.4732(6)	10.5215(17)	20.665(3)
c/Å	36.368(2)	11.169(2)	24.056(3)
α/°	90	78.074(14)	109.424(10)
β/°	90	74.195(14)	99.434(9)
γ/°	90	64.161(14)	100.158(11)
Volume/Å^3	4787.3(6)	971.8(3)	5007.4(12)
Z	4	1	2
ρ_{calc} g/cm^3	1.407	2.010	1.459
μ/mm^{-1}	1.943	8.529	1.577
F(000)	2112.0	568.0	2264.0
Crystal size/mm^3	0.18 × 0.157 × 0.12	0.19 × 0.09 × 0.02	0.32 × 0.157 × 0.05
Radiation	Mo Kα (λ = 0.71073)	Mo Kα (λ = 0.71073)	Mo Kα (λ = 0.71073)

2Θ range for data collection/°	3.55 to 53.964	3.81 to 50	2.258 to 51
Index ranges	-14 ≤ h ≤ 8, -14 ≤ k ≤ 12, -45 ≤ l ≤ 45	-11 ≤ h ≤ 10, -12 ≤ k ≤ 12, -13 ≤ l ≤ 13	-12 ≤ h ≤ 13, -25 ≤ k ≤ 25, -29 ≤ l ≤ 29
Reflections collected	15024	6327	33771
Independent reflections	10126 [R_{int} = 0.0425, R_{sigma} = 0.0334]	3409 [R_{int} = 0.1051, R_{sigma} = 0.1281]	18464 [R_{int} = 0.0981, R_{sigma} = 0.1059]
Data/restraints/parameters	10126/1/508	3409/254/215	18464/41/1085
Goodness-of-fit on F^2	1.014	1.113	1.164
Final R indexes [I>=2σ (I)]	R_1 = 0.0449, wR_2 = 0.1292	R_1 = 0.0938, wR_2 = 0.2049	R_1 = 0.1156, wR_2 = 0.2794
Final R indexes [all data]	R_1 = 0.0553, wR_2 = 0.1426	R_1 = 0.1650, wR_2 = 0.2556	R_1 = 0.1674, wR_2 = 0.3227
Largest diff. peak/hole / e Å^{-3}	0.65/-0.53	3.28/-3.01	3.19/-2.63
Flack parameter	-0.018(9)		

3. Determination of the Buried Volume

The steric properties of the phosphanide ligand could be determined using the *SambVca 2* web tool.[10,11] A buried volume of $\%V_{bur}$ = 37.3% was found.

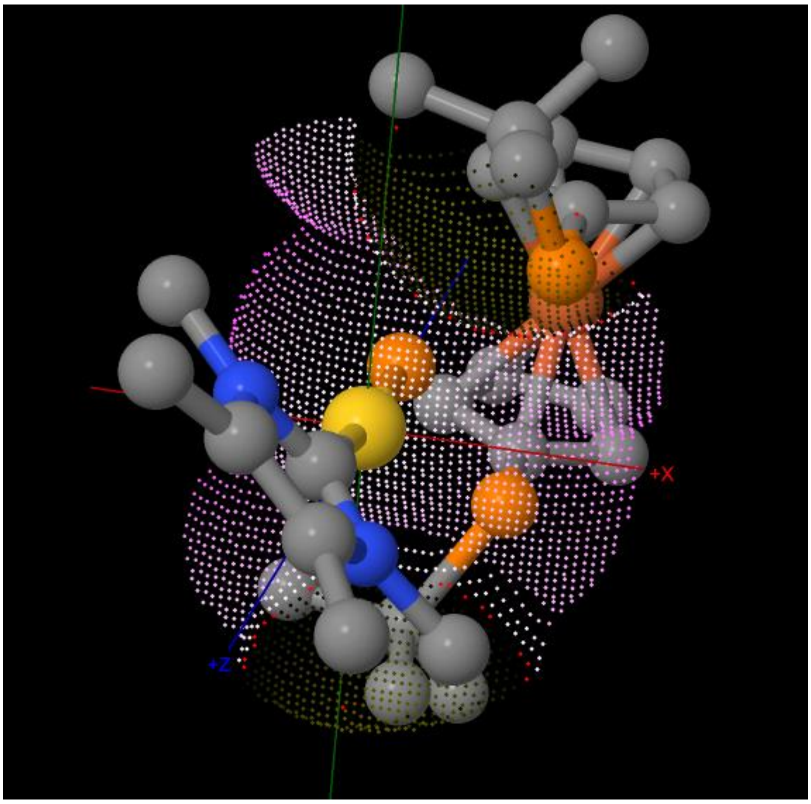

Figure S41: Graphical representation of the steric congestion imposed by the triphospha [3]-ferrocenophane-ligand on the coordinated Au-atom in **3-Au(IMe)**.

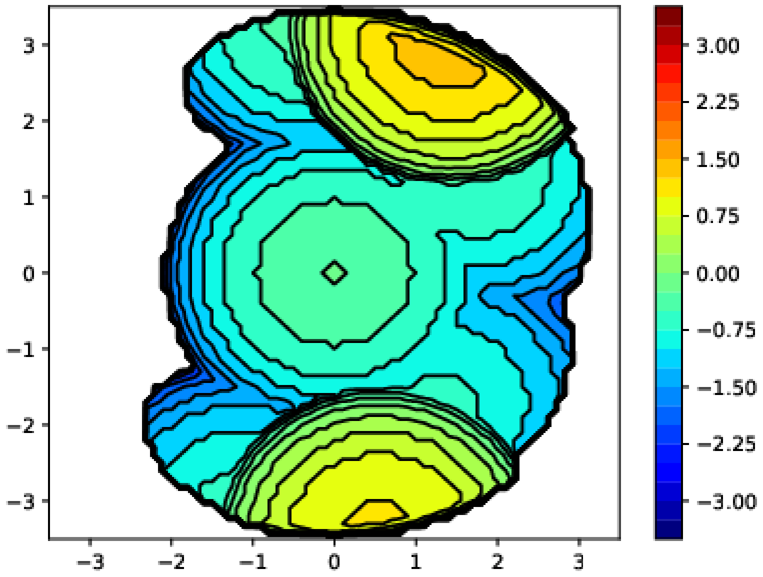

Figure S42: Steric map of the triphospha [3]-ferrocenophane-ligand in compound **3-Au(IMe)** as seen from the coordinated Au-atom. For calculations, an Au–P bond length of 2.32 Å was used.

4. Literature

[1] H. Tang, H. G. Richey, *Organometallics* **2001**, *20*, 1569–1574.

[2] M. Westerhausen, *Inorg. Chem.* **1991**, *30*, 96–101.

[3] K. Snégaroff, S. Komagawa, F. Chevallier, P. C. Gros, S. Golhen, T. Roisnel, M. Uchiyama, F. Mongin, *Chem. – A Eur. J.* **2010**, *16*, 8191–8201.

[4] F. Hanasaka, K. Fujita, R. Yamaguchi, *Organometallics* **2005**, *24*, 3422–3433.

[5] R. K. Harris, E. D. Becker, S. M. C. de Menezes, R. Goodfellow, P. Granger, *Pure Appl. Chem.* **2001**, *73*, 1795–1818.

[6] S. Bachmann, B. Gernert, D. Stalke, *Chem. Commun.* **2016**, *52*, 12861–12864.

[7] G. M. Sheldrick, *Acta Cryst. C* **2015**, *71*, 3–8.

[8] O. V. Dolomanov, L. J. Bourhis, R. J. Gildea, J. A. K. Howard, H. Puschmann, *J. Appl. Crystallogr.* **2009**, *42*, 339–341.

[9] C. F. MacRae, I. Sovago, S. J. Cottrell, P. T. A. Galek, P. McCabe, E. Pidcock, M. Platings, G. P. Shields, J. S. Stevens, M. Towler, P. A. Wood, *J. Appl. Crystallogr.* **2020**, *53*, 226–235.

[10] A. Poater, B. Cosenza, A. Correa, S. Giudice, F. Ragone, V. Scarano, L. Cavallo, *Eur. J. Inorg. Chem.* **2009**, *2009*, 1759–1766.

[11] L. Falivene, R. Credendino, A. Poater, A. Petta, L. Serra, R. Oliva, V. Scarano, L. Cavallo, *Organometallics* **2016**, *35*, 2286–2293.
